# Comparison of Ambulatory Health Care Costs and Use Associated With Roux-en-Y Gastric Bypass vs Sleeve Gastrectomy

**DOI:** 10.1001/jamanetworkopen.2022.9661

**Published:** 2022-05-02

**Authors:** Kristina H. Lewis, Stephanie Argetsinger, David E. Arterburn, Jenna Clemenzi, Fang Zhang, Ronald Kamusiime, Adolfo Fernandez, Dennis Ross-Degnan, James F. Wharam

**Affiliations:** 1 Department of Epidemiology & Prevention, Department of Implementation Science, Division of Public Health Sciences, Wake Forest University Health Sciences, Winston-Salem, North Carolina; 2 Division of Health Policy & Insurance Research, Department of Population Medicine, Harvard Pilgrim Healthcare Institute, Harvard Medical School, Boston, Massachusetts; 3 Kaiser Permanente Washington Health Research Institute, Seattle; 4 Department of Surgery, Wake Forest University Health Sciences, Winston-Salem, North Carolina; 5 Department of Medicine, Duke University, Durham, North Carolina; 6 Duke-Margolis Center for Health Policy, Durham, North Carolina

## Abstract

**Importance:**

Studies comparing contemporary bariatric surgical types could facilitate procedure selection for patients interested in reducing their frequency of health care visits and reliance on prescription drugs.

**Objective:**

To compare the association of sleeve gastrectomy (SG) and Roux-en-Y gastric bypass (RYGB) with ambulatory health care costs and use for as long as 4 years after surgery.

**Design, Setting, and Participants:**

This comparative effectiveness study, which included patients undergoing bariatric surgery who were aged 18 to 64 years with at least 24 months of enrollment data before surgery and 12 months of enrollment data after surgery, used a retrospective interrupted time series with a comparison group. Data represent insurance claims dated January 2006 to June 2017, with analyses completed in September 2021. Data were collected from US commercial and Medicare Advantage claims database. Cohorts were matched on characteristics including baseline body mass index category, diabetes status, baseline ambulatory care costs, region of the United States, and year of surgery.

**Exposures:**

SG or RYGB, based on procedure codes.

**Main Outcomes and Measures:**

Annual ambulatory health care costs, and subtypes of cost and use including prescriptions, office visits, laboratory encounters, and radiology.

**Results:**

Matched cohorts included 3049 patients who underwent SG and 3251 patients who underwent RYGB, with a mean (SD) age of 45.2 (10.0) years; 4820 (77%) were women. Full follow-up was 37% for SG (514 patients) and 38% for RYGB (643 patients) among those eligible for 4-year follow-up. There were no significant differences between SG and RYGB in total ambulatory costs, office visit costs, or radiology costs in all follow-up years. Patients who underwent SG had significantly higher prescription costs than those who underwent RYGB bypass in year 4 ($852.8 per patient per year; 95% CI: $395.6-$1310.0 per patient per year) with more cardiometabolic medication fills in each year (eg, year 4: 42.5%; 95% CI, 13.7%-71.2%). In contrast, early after surgery, patients who underwent SG had relatively fewer specialist visits (eg, year 1: −7.2%; 95% CI, −14.3% to −0.2%) and lower laboratory costs (eg, year 1: −$118.9 per patient per year; 95% CI, −$220.2 to −$17.5 per patient per year).

**Conclusions and Relevance:**

Despite clinical studies showing greater weight loss and comorbidity improvement with RYGB vs SG, this study found no difference in total ambulatory costs for as long as 4 years after SG and RYGB. These findings may reflect the trade-off between greater improvements in cardiometabolic health and additional surgery-related care among patients undergoing RYGB. Studies with longer follow-up time could determine whether greater sustained weight loss from RYGB eventually results in lower costs compared with SG.

## Key Points

**Question:**

What are the comparative associations of sleeve gastrectomy (SG) vs Roux-en-Y gastric bypass (RYGB) with patients’ ambulatory health care use and costs?

**Findings:**

In this comparative effectiveness study that included 6300 patients, total ambulatory costs were similar for as long as 4 years following SG and RYGB. However, RYGB was associated with greater reductions in prescriptions for cardiometabolic disease, while SG was associated with fewer specialist visits and laboratory tests after surgery.

**Meaning:**

These findings suggest that lesser need for cardiometabolic medications following RYGB vs SG may be counterbalanced by a greater need for postsurgical monitoring after this more invasive procedure.

## Teaser

This comparative effectiveness study evaluates the associations of sleeve gastrectomy and Roux-en-Y gastric bypass with ambulatory health care costs and use for 4 years after surgery.

## Introduction

No treatment for severe obesity has demonstrated as profound or durable an impact as bariatric surgery,^1,2,3^ both in terms of weight loss^2,3,4,5^ and remission from comorbidities such as diabetes^4,6,7,8^ and hypertension.^6,9^ Despite these clinical improvements, most studies of total medical expenditures have not found bariatric surgery to be cost-saving in the short to medium term^10,11,12,13^—in part because of complications and acute care encounters that may offset health gains.^13^

Even if not cost-saving, bariatric surgery has been shown to be cost-effective^14^ and is a primary guideline-recommended treatment option for patients with severe obesity.^15^ Patients pursuing surgery are faced with a choice of several different procedure types, each with different associated risks and benefits. The gold standard Roux-en-Y gastric bypass (RYGB) and newer sleeve gastrectomy (SG) together comprise 78% of the approximately 250 000 bariatric operations now performed annually in the United States (61% SG and 17% RYGB as of 2018),^16^ yet there are few studies directly comparing their impacts on health care use and costs.^17,18^

We and others have identified a greater risk of operative and nonoperative interventions^19,20,21^ and acute care use associated with RYGB compared with SG.^22,23^ However, whether these surgical types differentially affect ambulatory care use, which is more likely driven by ongoing chronic disease management, preventive health care, and routine postsurgical monitoring, is not known. Such information could facilitate procedure selection for patients interested in reducing their need for regular doctors’ visits and prescription drugs through surgery.

We used nationwide commercial insurance claims data to study changes in ambulatory health care use and costs for as long as 4 years after SG vs RYGB. We hypothesized that, relative to SG, RYGB would produce larger longer-term declines in medication costs, office visits, and overall ambulatory spending, based on its demonstrated superior clinical impact on weight and comorbidities, such as type 2 diabetes (T2D).^3,4,6,7,8,24^

## Methods

### Data Source and Study Design

In this comparative effectiveness study, we conducted an interrupted time series (ITS) with comparison group study, using data from a large national commercial (and Medicare Advantage) claims database, including enrollment and demographic information as well as inpatient, outpatient, and pharmacy claims from 2000 to 2017 for all members. This study used data from 2006 to 2017. The study was approved by the Harvard Pilgrim Health Care institutional review board, with a waiver of informed consent owing to the use of preexisting and deidentified data. This article was prepared in accordance with the International Society for Pharmacoeconomics and Outcomes Research (ISPOR) guidelines for comparative effectiveness research.^25^

### Study Population

We used previously published methods^19,24,26^ to identify adults aged 18 to 64 years who underwent a primary SG or RYGB between January 2008 and June 2016. To ensure a representative baseline that excluded the high-utilization period of presurgical clearance and to allow sufficient follow-up for analyses of annualized change, we required continuous enrollment (no gaps of >90 days at any point) for at least 24 months before and at least 12 months after an index procedure. Patients were followed up after surgery for as long as 48 months.

### Outcome Measures

We extracted medical and pharmacy claims for all person-time among identified members, then excluded any nonambulatory claims (eTable 1 in the Supplement). We grouped remaining claims into 1 of 5 categories: office visits, laboratory, radiology, pharmacy, and other.

#### Primary Outcomes: Costs Associated With Ambulatory Care

Costs per category were measured in 2017 US dollars using the vendor’s claim-level standardized cost variable, which eliminates pricing variability across calendar time (inflation) and region. Nonzero costs per quarter were winsorized at the 99th percentile in each of the 5 main categories to reduce the effect of high outliers. We summed winsorized total person-level costs quarterly and annually, overall and within each outcome category.

#### Secondary Outcomes: Ambulatory Care Encounter Types

To better understand the clinical factors associated with pre-to-post–surgical differences in ambulatory cost changes, we examined subtypes of care encounters (eTable 1 in the Supplement). Using the American Hospital Formulary Service designation,^27^ prescription encounters were categorized into medication fills for cardiometabolic disease (including T2D, hypertension, and dyslipidemia) and all other indications (eg, mental illness). Office visits were divided using an established algorithm based on clinician-type variables and billing codes into primary care physician (PCP) visits and specialist visits. A clinician on the team (K.H.L.) grouped laboratory and radiology encounters into categories based on diagnostic or procedure codes. Laboratory encounters were grouped as either nutrition (eg, vitamin B12) or other (eg, hematology). Radiology encounters were divided by body site being imaged into abdomen or pelvis and other body site.

### Other Measures

Demographic measures included age, sex, and US region of residence on the date of surgery. Area-level measures based on the American Community Survey included neighborhood (census block group) racial composition,^28,29,30^ education level, and poverty level on the date of surgery.^28,31^ We used Johns Hopkins ACG software^32^ to estimate overall presurgical morbidity and specific comorbidities, except T2D, which was categorized using diagnoses then subcategorized based on presence of baseline insulin use.^24^ These measures were assessed across the 12 months immediately before surgery. Preoperative body mass index (BMI; calculated as weight in kilograms divided by height in meters squared) was categorized into groups using the last diagnosis code before the date of surgery. Where possible, specific diagnosis codes corresponding to a narrow range of BMI (*z* codes) were used, a previously validated method.^7^ Because of secular trends in technique and safety, operations were also categorized based on calendar year (2008-2011, 2012-2014, and 2015-2016).

### Matching Strategy

To create SG and RYGB groups balanced on factors associated with baseline health care use and procedure choice, we used a hybrid coarsened exact and propensity matching approach.^19,24,26,33^ We exact matched the groups on baseline BMI category, diabetes status and insulin use, total baseline ambulatory care cost quartile (calculated during months −24 to −13 before surgery to avoid costs associated with preoperative workup), US region of residence, and calendar year group. We also exact matched on tertile of a propensity score that included patient sex, age group, ACG score (<3 vs ≥3), and neighborhood demographic characteristics. This matching approach up- or down-weighted patients in the comparison (ie, RYGB) group within exact matching strata to ensure balance. We considered the groups well-matched provided that the residual postmatch standardized difference between them was smaller than an absolute value of 0.2.

### Statistical Analysis

#### ITS Plots

For all outcomes, we created ITS plots of the mean per-member-per-quarter value (dollars for cost outcomes, number of encounters for count outcomes) from 2 years prior through 4 years after surgery in our matched cohorts. Statistical significance was set at α < .05, and tests were 2-tailed.

#### Between-Procedure Differences

We used difference-in-differences (DiD) analyses to compare pre-to-post–surgical changes in annualized outcome measures between patients undergoing SG and RYGB. We compared postoperative measures with measures from a preoperative baseline period consisting of months −24 to −13 before the index surgical date (year −2). We did not select the year immediately before surgery (year −1) as the baseline because it typically includes an intense preoperative workup with atypically high costs and utilization.^12^ To ensure the validity of the DiD approach, a parallel trends test was conducted for our primary (cost) outcomes using the 4 quarters in year −2.

For all cost outcomes and most encounter outcomes, we modeled the DiD with zero-inflated negative binomial models to account for potential excess zeros.^34,35^ To account for different prevailing views on the optimal method for modeling health care cost data, we also ran cost models using a 2-part model with generalized gamma distribution (eTable 2 in the Supplement).^36^ Models included matching variables as covariates to adjust for potential differential dropout between groups over follow-up. All models used the coarsened exact matching weights, an offset term to account for partial-year enrollment beyond year 1, and accounted for clustering within patient over time. We used Stata version 16 (StataCorp) to conduct the matching and regression analyses. To estimate the potential that unmeasured confounders accounted for our results, we calculated E-values for all statistically significant findings^37,38^ (eTable 3 in the Supplement).

#### Sensitivity Analysis

Because of high rates of disenrollment over follow-up, we examined whether our primary results were affected by dropout. We did this by repeating the 6 DiD cost analyses on a rematched subcohort of patients with complete 4-year enrollment.

## Results

### Study Population

Our primary matched cohorts included 3049 patients who underwent SG and 3251 who underwent RYGB (eFigure in the Supplement). Across the cohorts, mean (SD) age was 45.2 (10.0) years, 4820 (77%) were women, and 3374 (53%) resided in majority White neighborhoods. Median (IQR) postsurgical follow-up time did not differ substantially between SG (2.5 [1.6-3.5] years) and RYGB (2.4 [1.8-3.6] years). Among patients with operations 4 or more years before the end of the data set, 1157 (38%; 514 undergoing SG, and 643 undergoing RYGB) remained enrolled 4 years postoperatively. Follow-up and percentage observed by year are detailed in eTable 4 in the Supplement. The matched SG and RYGB groups in both our primary and 4-year continuous follow up cohorts were well-balanced on measured baseline characteristics (Table 1; eTable 5 and eTable 6 in the Supplement).

**Table 1.** Presurgery Characteristics of Unmatched and Matched Cohorts of Patients With Index SG and RYGB Between 2008 and 2016

Variable^b^	Participants before matching, No. (%)		Standardized difference^c^	Participants after matching, No. (%)^a^		Standardized difference^c^
	RYGB (N = 3955)	SG (N = 3955)		RYGB (N = 3251)	SG (N = 3049)	
Year of surgery						
2008-2011	2732 (69.1)	745 (18.8)	1.2	741 (22.8)	695 (22.8)	0.00
2012-2014	1018 (25.7)	1956 (49.5)		1757 (54.1)	1648 (54.1)	
2015-2016	205 (5.2)	1254 (31.7)		753 (23.2)	706 (23.2)	
Age ≥40 y	2798 (70.8)	2770 (70.0)	−0.02	2223 (68.4)	2140 (70.2)	0.04
Sex						
Female	3037 (76.8)	2951 (74.6)	−0.05	2518 (77.4)	2302 (75.5)	−0.05
Male	918 (23.2)	1004 (25.4)		733 (22.6)	747 (24.5)	
White neighborhood, ≥75%^d^	2156 (54.5)	2065 (52.2)	−0.05	1773 (54.5)	1601 (52.5)	−0.04
Neighborhood poverty^e^						
Less poor (<10%)	1845 (46.7)	1968 (49.7)	−0.06	1566 (48.2)	1507 (49.4)	−0.03
More poor (≥10%)	2097 (53.0)	1976 (50.0)		1685 (51.9)	1542 (50.6)	
Missing	13 (0.3)	11 (0.3)				
Region of United States						
West	819 (20.7)	685 (17.3)	0.15	521 (16.0)	489 (16.0)	0.00
South	1868 (47.2)	2041 (51.6)		1836 (56.5)	1722 (56.5)	
Midwest	878 (22.2)	732 (18.5)		613 (18.9)	575 (18.9)	
Northeast	376 (9.5)	492 (12.4)		280 (8.6)	263 (8.6)	
Missing	14 (0.4)	5 (0.1)		0	0	
BMI category^f^						
30-39.9	427 (10.8)	649 (16.4)	0.38	423 (13.0)	397 (13.0)	0.00
40-49.9	2014 (50.9)	2072 (52.4)		1945 (59.8)	1824 (59.8)	
50-59.9	379 (9.6)	598 (15.1)		438 (13.5)	411 (13.5)	
≥60	77 (1.9)	141 (3.6)		37 (1.1)	35 (1.1)	
Non-specific obesity	1058 (26.8)	495 (12.5)		407 (12.5)	382 (12.5)	
ACG Score						
≥3	1409 (35.6)	1422 (36.0)	0.01	1156 (35.6)	1063 (34.9)	−0.02
Mean (SD)	3.1 (3)	3.0 (3)	−0.03	3.2 (3)	2.9 (3)	−0.07
Type 2 diabetes	1756 (44.4)	1353 (34.2)	−0.21	1010 (31.1)	947 (31.1)	0.00
Insulin use	511 (12.9)	257 (6.5)	−0.22	170 (5.2)	159 (5.2)	0.00
Hypertension	2480 (62.7)	2340 (59.2)	−0.07	1811 (55.7)	1795 (58.9)	0.06
Cardiovascular disease	506 (12.8)	462 (11.7)	−0.03	319 (9.8)	346 (11.3)	0.05
Psychiatric illness	1022 (25.8)	959 (24.2)	−0.04	831 (25.6)	738 (24.2)	−0.03
Total ambulatory medical costs in baseline year^g^						
$0-$1453.68	883 (22.5)	1078 (27.4)	0.194	911 (28.0)	854 (28.0)	0.00
$1453.69-$4227.36	916 (23.3)	1049 (26.6)		868 (26.7)	814 (26.7)	
$4227.37-$10 292.07	1034 (26.3)	938 (23.8)		744 (22.9)	698 (22.9)	
$10 292.08-$115 314.69	1095 (27.9)	874 (22.2)		728 (22.4)	683 (22.4)	

Abbreviations: ACG, Johns Hopkins System for comorbidity estimation; BMI, body mass index (calculated as weight in kilograms divided by height in meters squared); RYGB, Roux-en-Y gastric bypass; SG, sleeve gastrectomy.

^a^ Coarsened exact matching was conducted on BMI category, diabetes status and insulin use, total ambulatory care cost quartile during the presurgical year, region of the United States, and calendar period as well as tertile of a propensity score that included patient sex, age group, ACG score (<3 vs ≥3), and neighborhood demographic characteristics.

^b^ The Methods section includes complete descriptions of how baseline variables were constructed.

^c^ Standardized differences are the difference in means between intervention and control divided by the SD of the difference in means. Lower absolute values indicate greater similarity, and values less than 0.2 indicate minimal differences between groups.

^d^ White neighborhoods defined as census tracts where more than 75% of residents were Non-Hispanic White individuals.

^e^ Neighborhoods with more poverty were those where at least 10% of households were below the poverty line.

^f^ BMI based on most recent presurgery diagnosis.

^g^ Cost categories as shown represent quartiles of total ambulatory costs (summing all non–emergency department, nonhospital health care and prescription costs) across all unmatched RYGB and SG patients in year −2 prior to surgery. These quartiles were used in the coarsened exact matching to balance groups with respect to baseline outpatient medical spending. Year −2 was chosen as baseline for costs, as opposed to year −1, to avoid capturing the many costs associated with the procedures themselves as patients pursued preoperative workup and clearance. Costs are standardized by the data vendor to 2017 US dollars using a method that eliminates pricing variability across calendar time and geography. Total ambulatory medical cost quartiles were calculated among the patients without missing propensity scores: 3928 in the RYGB group and 3839 in the SG group.

### Total Ambulatory Costs

**Figure 1.**
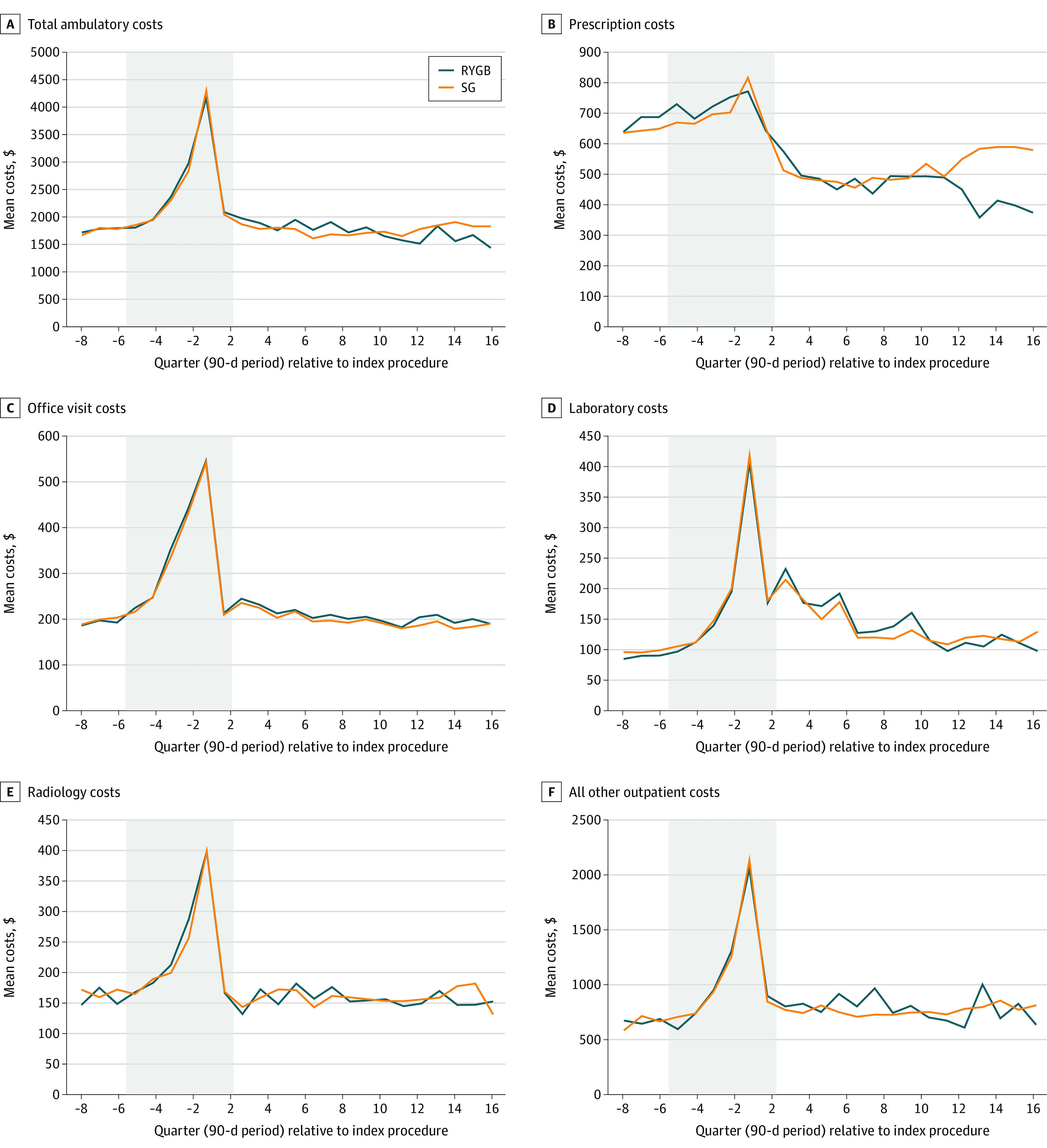
Interrupted Time Series Plots Comparing Patients Undergoing Sleeve Gastrectomy (SG) vs Roux-en-Y Gastric Bypass (RYGB) Between 2008 and 2016 Across Ambulatory Care Cost Categories Adjusted time series plots of per member per quarter outpatient costs before and after surgery. Costs per claim were measured in 2017 US dollars. Costs were winsorized at the 99th percentile at the quarterly level in the prescription, office visit, laboratory, and radiology categories to reduce the effect of high outliers. Gray boxes represent unanalyzed baseline period through index surgery but are shown so that the high-cost presurgical and perioperative periods can be represented relative to true baseline and follow-up. A, Baseline (year −2 prior to surgery), mean (SD) total ambulatory costs were $7117 ($9655) per person per year for SG and $8471 ($10 919) per person per year for RYGB. B, Baseline (year −2 prior to surgery), mean (SD) prescription costs were $2606 ($4739) per person per year for SG and $3581 ($5488) per person per year for RYGB. C, Baseline (year −2 prior to surgery), mean (SD) office visit costs were $780 ($808) per person per year for SG and $854 ($857) per person per year for RYGB. D, Baseline (year −2 prior to surgery), mean (SD) laboratory costs were $396 ($641) per person per year for SG and $374 ($608) per person per year for RYGB. E, Baseline (year −2 prior to surgery), mean (SD) radiology costs were $643 ($1202) per person per year for SG and $706 ($1289) per person per year for RYGB. F, Baseline (year −2 prior to surgery), mean (SD) all other outpatient costs were $2673 ($6273) per person per year for SG and $2956 ($6672) per person per year for RYGB.

ITS plots demonstrated a sharp rise in total ambulatory costs in the year before surgery that peaked in the perioperative period before returning to near baseline (Figure 1A). There were no statistically significant differences between SG and RYGB for pre-post total annual ambulatory cost changes during 4 years of follow-up (Table 2).

**Table 2.** Results From Multivariable Difference-in-Differences Analyses Comparing Patients Undergoing SG vs RYGB Between 2008 and 2016 Across Categories of Ambulatory Care Costs and Use^a^

Cost category	Postoperative year 1, SG (n = 3049) vs RYGB (n = 3251)		Postoperative year 2, SG (n = 1952) vs RYGB (n = 2173)		Postoperative year 3, SG (n = 1054) vs RYGB (n = 1151)		Postoperative year 4, SG (n = 514) vs RYGB (n = 643)	
	Absolute difference (95% CI), $^b^	Relative difference, % (95% CI)^c^	Absolute difference (95% CI), $^b^	Relative difference, % (95% CI)^c^	Absolute difference (95% CI), $^b^	Relative difference, % (95% CI)^c^	Absolute difference (95% CI), $^b^	Relative difference, % (95% CI)^c^
Total ambulatory care costs	−421.9 (−1334.1 to 490.4)	−5.3 (−16.1 to 5.6)	−642.9 (−1643.4 to 357.5)	−8.4 (−20.5 to 3.7)	−138.9 (−1273.2 to 995.4)	−2.0 (−17.8 to 13.9)	382.8 (−1366.0 to 2131.6)	5.1 (−19.2 to 29.5)
Prescription drug costs	36.1 (−252.0 to 324.3)	1.7 (−11.9 to 15.2)	173.3 (−80.1 to 426.7)	9.7 (−5.6 to 25.1)	140.2 (−283.0 to 563.3)	7.1 (−15.6 to 29.9)	852.8 (395.6 to 1310.0)^d^	52.4 (17.8 to 87.0)^e^
Office visit costs	−36.7 (−103.5 to 30.1)	−4.1 (−11.2 to 3.1)	−14.4 (−85.4 to 56.5)	−1.7 (−10.1 to 6.6)	−2.0 (−87.1 to 83.1)	−0.3 (−11.1 to 10.6)	−43.8 (−176.1 to 88.5)	−5.3 (−20.5 to 10.0)
Laboratory costs	−118.9 (−220.2 to −17.5)^f^	−13.9 (−24.2 to −3.6)^e^	−107.6 (−197.2 to −18.1)^f^	−15.9 (−27.4 to −4.5)^e^	−106.7 (−244.5 to 31.1)	−18.0 (−37.5 to 1.4)	−4.0 (−116.7 to 108.7)	−0.8 (−23.6 to 22.0)
Radiology costs	−4.6 (−97.7 to 88.5)	−0.7 (−15.5 to 14.1)	−63.6 (−189.7 to 62.6)	−9.2 (−26.0 to 7.6)	6.7 (−121.7 to 135.0)	1.1 (−20.3 to 22.5)	22.5 (−150.8 to 195.7)	3.4 (−23.7 to 30.6)
All other outpatient costs	−264.7 (−945.4 to 416.1)	−7.7 (−26.1 to 10.7)	−600.9 (−1432.8 to 231.0)	−16.4 (−35.7 to 3.0)	−164.6 (−1103.8 to 774.6)	−5.1 (−33.2 to 22.9)	−537.1 (−1900.9 to 826.7)	−13.9 (−45.1 to 17.2)

Abbreviations: RYGB, Roux-en-Y gastric bypass; SG, sleeve gastrectomy.

^a^ Difference-in-differences analyses adjusting for all matched variables were used to generate between-group estimates for change in each outcome, at each time period, relative to a preoperative baseline period spanning months −24 to −13 before the index surgical date. Year −2 was selected as the preoperative baseline for comparison because the year immediately before surgery represents a very high utilization time, owing to preoperative workup.

^b^ Absolute differences in each period refer to the estimated actual change in costs among patients undergoing SG minus those among patients undergoing RYGB in that segment vs baseline (months −24 to −13), accounting for all other periods.

^c^ Relative differences in each period refer to the estimated relative difference between SG and RYGB groups vs baseline, accounting for all other periods.

^d^ *P* < .001.

^e^ *P* < .01.

^f^ *P* < .05.

### Prescriptions

**Figure 2.**
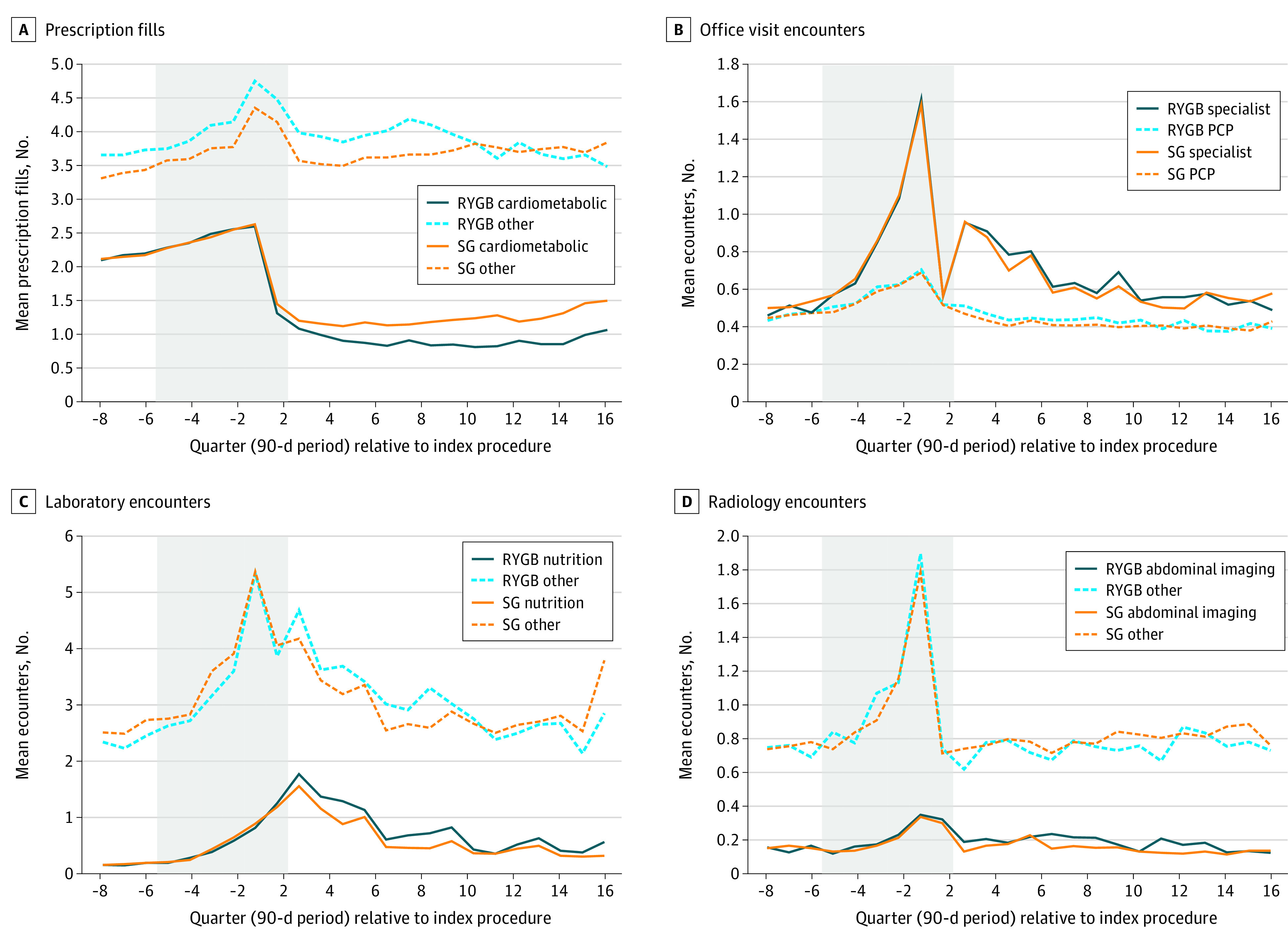
Interrupted Time Series Plots Comparing Patients Undergoing Sleeve Gastrectomy (SG) vs Roux-en-Y Gastric Bypass (RYGB) Between 2008 and 2016 Across Suboutcomes of Ambulatory Care Encounters Adjusted time series plots of outpatient encounters before and after surgery. A, Prescription fills were divided based on the American Hospital Formulary Service designation, into fills of medications for cardiometabolic disease (including type 2 diabetes, hypertension, and dyslipidemia), and all other indications (including pain, mental illness, antimicrobials, and others). B, Office visits were divided using an established algorithm based on clinician-type variables and diagnostic codes into primary care physician (PCP) visits and specialist visits. C, Laboratory encounters were grouped as either nutrition (eg, iron, copper, vitamin D) or other (eg, hematology, chemistry, microbiology, toxicology, endocrine). D, Radiology encounters were divided by body site being imaged, into abdomen or pelvis, and other body site.

Prescription costs decreased after surgery in both the SG and RYGB cohorts (Figure 1B). Significant between-procedure differences in prescription cost changes did not emerge until postoperative year 4, when estimated costs were higher for the SG cohort than the RYGB cohort (absolute difference $852.8 per patient per year [95% CI, $395.6-$1310.0 per patient per year]) (Table 2). Patients undergoing SG had more cardiometabolic prescription fills than those undergoing RYGB in all postoperative years. For example, cardiometabolic fills for patients undergoing SG were estimated to be 42.5% (95% CI, 13.7%-71.2%) higher than those for patients undergoing RYGB by year 4. (Table 3 and Figure 2A). DiD models found no difference in change for other prescription fills between procedures. Compared with cardiometabolic prescription fills, this group of other prescriptions accounted for a larger number of pharmacy encounters for both cohorts before and after surgery (Figure 2A).

**Table 3.** Results From Multivariable Difference-in-Differences Analyses Comparing Patients Undergoing SG vs RYGB Between 2008 and 2017 Across Multiple Encounter Subtypes^a^

Cost category	Postoperative year 1, SG (n = 3049) vs RYGB (n = 3251)		Postoperative year 2, SG (n = 1952) vs RYGB (n = 2173)		Postoperative year 3, SG (n = 1054) vs RYGB (n = 1151)		Postoperative year 4, SG (n = 514) vs RYGB (n = 643)	
	Absolute difference (95% CI)^b^	Relative difference, % (95% CI)^c^	Absolute difference (95% CI)^b^	Relative difference, % (95% CI)^c^	Absolute difference (95% CI)^b^	Relative difference, % (95% CI)^c^	Absolute difference (95% CI)^b^	Relative difference, % (95% CI)^c^
Prescription encounters								
Cardiometabolic prescription fills	0.7 (0.3 to 1.1)^d^	16.6 (6.3 to 26.9)^e^	1.1 (0.7 to 1.5)^d^	31.0 (16.6 to 45.5)^d^	1.5 (0.9 to 2.1)^d^	42.2 (21.5 to 62.8)^d^	1.7 (0.8 to 2.6)^d^	42.5 (13.7 to 71.2)^e^
All other prescription fills	−0.5 (−1.4 to 0.5)	−3.0 (−9.0 to 2.9)	−0.1 (−1.2 to 0.9)	−1.0 (−8.3 to 6.3)	0.4 (−1.0 to 1.9)	2.8 (−7.2 to 12.7)	1.4 (−0.5 to 3.3)	10.1 (−4.5 to 24.8)
Office visit encounters^f^								
Specialist visits	−0.2 (−0.5 to 0.0)	−7.2 (−14.3 to −0.2)^g^	−0.2 (−0.5 to 0.0)	−7.7 (−16.0 to 0.5)	−0.3 (−0.6 to 0.0)^g^	−11.3 (−21.2 to −1.4)^g^	0.1 (−0.2 to 0.3)	2.7 (−9.7 to 15.0)
PCP visits	−0.1 (−0.2 to 0.0)	−4.3 (−9.7 to 1.1)	−0.1 (−0.2 to 0.0)	−4.7 (−11.0 to 1.5)	−0.1 (−0.2 to 0.1)	−3.8 (−12.0 to 4.4	0.0 (−0.1 to 0.2)	1.4 (−8.7 to 11.6)
Laboratory testing encounters								
Nutrition	−1.2 (−2.2 to −0.2)^g^	−20.0 (−34.0 to −6.0)^e^	−0.8 (−1.5 to −0.2)^g^	−24.3 (−39.2 to −9.5)^e^	−0.5 (−1.0 to 0.0)	−20.3 (−38.3 to −2.3)^g^	−0.7 (−1.2 to −0.1)^g^	−30.4 (−48.8 to −11.9)^e^
All other	−2.5^e^ (−4.1 to −1.0)	−14.5^d^ (−22.4 to −6.7)	−2.5^e^ (−4.1 to −0.8)	−17.4^d^ (−27.0 to −7.7)	−0.7 (−2.3 to 0.9)	−5.7 (−18.7 to 7.4)	−0.3 (−3.2 to 2.6)	−2.3 (−26.3 to 21.6)
Radiology encounters								
Abdomen or pelvic imaging	−0.2 (−0.4 to 0.1)	−18.7 (−38.8 to 1.5)	−0.2 (−0.5 to 0.0)	−22.7 (−44.6 to −0.8)^g^	−0.1 (−0.3 to 0.1)	−14.4 (−44.3 to 15.5)	−0.1 (−0.4 to 0.1)	−19.7 (−51.6 to 12.1)
All other imaging	0.1 (−0.3 to 0.4)	2.7 (−10.2 to 15.6)	0.2 (−0.2 to 0.6)	6.2 (−8.5 to 20.9)	0.4 (−0.1 to 1.0)	14.1 (−6.3 to 34.6)	0.3 (−0.3 to 0.9)	9.0 (−11.5 to 29.5)

Abbreviations: PCP, primary care physician; RYGB, Roux-en-Y gastric bypass; SG, sleeve gastrectomy.

^a^ Difference-in-differences analyses using zero-inflated negative binomial models and adjusting for all matched variables were used to generate between-group estimates for change in each outcome, at each time period, relative to a preoperative baseline period spanning months −24 to −13 before the index surgical date. Year −2 was selected as the preoperative baseline for comparison because the year immediately before surgery represents a very high utilization time, owing to preoperative workup.

^b^ Absolute differences in each period refer to the estimated actual change in costs among patients undergoing SG minus those among patients undergoing RYGB in that segment vs baseline (months −24 to −13), accounting for all other periods.

^c^ Relative differences in each period refer to the estimated relative difference between SG and RYGB groups vs baseline, accounting for all other periods. In interpreting these relative differences, it should be noted that for outcomes that are relatively rare (eg, per-person use of abdominal imaging), relative difference estimates may give the appearance of greater difference between groups than the true absolute difference. Both absolute and relative differences are presented here for context and consistency with cost modeling results.

^d^ *P* < .001.

^e^ *P* < .01.

^f^ Specialist visits and PCP visits were modeled using negative binomial models (not zero-inflated).

^g^ *P* < .05.

### Office Visits

Changes in office visit costs did not differ between procedures through 4 postoperative years (Table 2 and Figure 1C). In the year before surgery, ITS plots of encounters grouped by PCP vs specialist visits revealed a clear ramp up in specialist encounters and a smaller increase in PCP encounters for both procedures (Figure 2B). Encounter-level DiD models showed a slight relative decrease in specialist visits for the SG cohort vs the RYGB cohort in years 1 (−7.2% [95% CI, −14.3% to −0.2%]) and 3 (−11.3% [95% CI, −21.2% to −1.4%]) (Table 3). Change in frequency of PCP visits did not differ between SG and RYGB cohorts during any of the 4 follow-up years.

### Laboratory

Laboratory costs increased sharply for both SG and RYGB in the immediate preoperative year, then gradually trended down near baseline levels after 4 years (Figure 1D). In DiD models, patients undergoing SG had relatively lower laboratory costs than those undergoing RYGB in postoperative years 1 (−$118.9 per patient per year [95% CI, −$220.2 to −$17.5 per patient per year]) and 2 (−$107.6 per patient per year [95% CI, −$197.2 to −$18.1 per patient per year]) (Table 2), but there was no detectable between-procedure difference in years 3 and 4. In encounter-level plots, a similar increase in frequency can be seen for both nutritional laboratory and other laboratory encounter types (Figure 2C). In encounter-level DiD models, patients undergoing SG had relative decreases in nutritional laboratory encounters compared with patients undergoing RYGB in all 4 postoperative years (Table 3). For non–nutritional laboratory encounters, we found similar relative decreases among patients undergoing SG in years 1 and 2 but no differences during years 3 and 4.

### Radiology Costs and Encounter Types

Similar to other cost categories, ambulatory radiology costs in ITS plots demonstrated a preoperative spike, but in the examined postoperative period, quickly returned to near baseline for both SG and RYGB cohorts (Figure 1E). In DiD models, changes in total radiology costs did not differ between procedure types over the 4-year post-operative period (Table 2). Encounter-level models showed that patients undergoing SG experienced similar rates of abdominal and other types of ambulatory imaging as those undergoing RYGB in all 4 postoperative years (Table 3).

### Other Ambulatory Costs

ITS plots for other ambulatory costs showed a similar pattern to other cost subtypes, with SG and RYGB cohorts experiencing a preoperative and perioperative spike in costs that returned to a similar level as year −2 over follow-up years 1 to 4 (Figure 1F). DiD models showed no significant between-procedure difference in change across the 4 postoperative years examined (Table 2).

### Sensitivity Analyses

Among patients with 4 years of continuous follow-up, rematched cohorts included 392 patients undergoing SG and 687 patients undergoing RYGB (eTable 5 in the Supplement). A comparison of this sensitivity cohort with our main analytic cohort (eTable 6 in the Supplement) revealed that these patients were similar to our main cohort on most measured factors. Sensitivity models of our 6 primary cost outcomes yielded results that were largely consistent with those in our main analysis, but with loss of statistical significance for the estimates of lower laboratory costs among the SG cohort compared with the RYGB cohort in the first 2 postoperative years (eTable 7 in the Supplement vs Table 2).

## Discussion

Contrary to our hypotheses, RYGB was not associated with lower ambulatory care costs than SG in the first 4 postoperative years. Underlying these top-line results was an association of RYGB with fewer prescription fills for cardiometabolic disease, yet relatively more health care use and costs for other ambulatory services. Our results suggest that surgery-related care and monitoring in the first few years of the postoperative period may counteract an otherwise decreased need for chronic disease care conferred by RYGB.

These findings add to emerging evidence about the global impact of modern bariatric procedures on costs and utilization and could enhance discussions about procedure choice. Despite its known cardiometabolic benefits, recent studies from our group^22^ and others^18,20^ have identified increased complication risks and high-acuity care in the first few years following RYGB compared with SG. For insurers and surgeons who are weighing these known risks of RYGB against its clinical benefits, the lack of difference in total ambulatory care costs that we observed may favor the ongoing preferential use of the slightly less costly and invasive SG. However, a closer look at our findings for several components of ambulatory costs and use supports a more optimistic outlook for RYGB in the long term.

Prior studies have identified similar reductions in pharmacy spending early after bariatric surgery,^10,11,39^ but we add to this literature by finding that RYGB patients had sustained, substantially lower use of cardiometabolic drugs than patients undergoing SG (Figure 2), aligning with clinical studies^3,4,6,8,24^ showing RYGB’s greater impact on obesity-related chronic disease. This type of analysis will need to be replicated in larger data sets with longer follow-up to determine whether RYGB does result in more durable long-term impacts on prescription costs (and, consequently, total medical costs) than SG.

Office visit cost trajectories were almost indistinguishable between SG and RYGB. Given the known differences in invasiveness and clinical impact between these procedures, this similarity suggests that patients undergoing bariatric surgery see their doctors at preprescribed, regular intervals regardless of procedure type, both for presurgical work-up and postsurgical monitoring. Supporting this idea, both groups had increases in specialist encounters around the 6-month, 1-year, and 2-year postoperative points. When we examined the type of specialist visits that were most common in these intervals (data not shown), general surgeon visits predominated. Also, as suggested by our ITS plots and other cohort studies, this increase in specialist (ie, surgeon) care may be temporary.^10,11,13^ Therefore, it is possible that as more care is provided by PCPs, decreases in office visits and associated costs would be observed.

Laboratory costs were relatively higher for the RYGB cohort than the SG cohort in the first several postoperative years, possibly because of more intensive guideline-recommended postoperative monitoring^40^ and more symptoms prompting evaluation. However, as with specialist visits, by approximately 3 years after surgery, both the SG and RYGB groups returned to near their preoperative baseline for laboratory costs as well as nutrition and other laboratory encounters. Although SG had relatively lower laboratory costs than RYGB in years 1 and 2 after surgery, this cost category was one of the least expensive ones we examined (eg, approximately $150 per member per quarter for laboratory costs vs approximately $500 per member per quarter for prescription costs after surgery), potentially explaining why no overall ambulatory cost savings were seen for SG relative to RYGB.

### Limitations

This study has limitations, including substantial cohort attrition over follow-up, as expected with commercial claims data sets, with 83% of the total sample not having a full 4 years of data. The true loss to follow-up rate (using a denominator of patients who would even be eligible for 4-year follow-up based on surgical date) is slightly lower, at 62%. Our sensitivity cohort with full 4-year follow-up, however, produced similar results for years 1 to 3 after surgery and was similar on most measurable characteristics compared with the main analytic cohort. Another limitation is that we could not identify patients who died. However, because death is quite rare following bariatric surgery (1-year mortality estimated at 0.1% for laparoscopic SG and at 0.2% for RYGB),^41^ our results should not be materially affected.

In observational studies such as this, despite matching on possible confounders, unmeasured factors, including characteristics of patients or clinicians that differed systematically between the SG and RYGB cohorts, may have influenced our findings. Additionally, our analyses are uninformative regarding the potential impact of these procedures relative to a nonsurgical control group. Although our ITS plots suggest a decrease in ambulatory cost trajectory for both procedures relative to the year −2 baseline (Figure 1A), we did not pursue comparison to a nonsurgical group because of the inability to adequately address unmeasured confounders. Another limitation is that our data include operations performed as far back as 2010; thus, results from these earlier procedures may not generalize fully to present-day surgical patients owing to improvements in surgical technique and broader changes in the care of patients with obesity over the past decade. Additionally, we were unable to determine the differential impact of SG vs RYGB on patient quality of life, as our analyses focused solely on health care costs and use.

## Conclusions

Despite its superior clinical impact on weight and weight-related comorbidities, we did not observe lower total ambulatory care costs up to 4 years after RYGB relative to SG. In fact, certain types of ambulatory care were more common following RYGB, indicating that at least in the first few years after surgery, its relative health improvements are counterbalanced by increased laboratory testing and specialist visits. Combined with prior studies showing higher early complication and acute care rates following RYGB, this study underscores why SG has emerged as the dominant procedure globally. However, remaining questions include whether, with longer-term follow-up, ambulatory spending could be lower for RYGB based on a greater or more durable benefit for cardiometabolic disease.

## Notes

Supplement. **eTable 1.** Algorithm to Identify Outpatient Episodes in Claims Data from 2008 to 2017 **eTable 2. **Results from Multivariable Difference-in-Differences Analyses Comparing Patients Undergoing SG vs RYGB Between 2008 and 2016 Across Categories of Ambulatory Care Costs and Use With a Gamma Distribution **eTable 3.** E-values and Interpretations for All Models with Statistically Significant Findings Comparing Patients Undergoing SG vs RYGB Between 2008 and 2016 **eFigure. **Flow Diagram for Cohort Selection of Patients with Index SG and RYGB Between 2008 and 2016 **eTable 4. **Attrition at Each Postoperative Year for Patients Undergoing SG or RYGB Between 2008 and 2016 **eTable 5. **Examining for Differential Attrition by Patient Characteristics: Baseline Characteristics of Patients in Main Analytic Cohort Enrolled at 1 and 4 Years After Surgery **eTable 6. **Presurgery Characteristics of the Matched Main Cohort vs the Sensitivity Cohort of Patients With Index SG and RYGB Between 2008 and 2016 **eTable 7.** Results From Multivariable Difference-in-Differences Analyses Comparing Patients Undergoing SG vs RYGB Between 2008 and 2016 Across Categories of Ambulatory Care Costs and Use Among Those With 4 Years of Follow-up **eTable 8.** Results From Multivariable Difference-in-Differences Analyses Comparing Patients Undergoing SG v. RYGB Between 2008 and 2016 Across Suboutcomes of Ambulatory Care Costs and Use Among Those With 4 Years of Follow-Up **eTable 9. **Stata Outputs for All Models **eTable 10. **Final Time Series Model After Backward Variable Selection for the Differenced Outcomes Between SG and RYBG
eReferences.
