# Supplementary material for: Comparison of Ambulatory Health Care Costs and Use Associated With Roux-en-Y Gastric Bypass vs Sleeve Gastrectomy
Source: JAMA Netw Open. 2022 May 2;5(5):e229661. doi: 10.1001/jamanetworkopen.2022.9661 (PMC9062690; doi:10.1001/jamanetworkopen.2022.9661)
Supplement: Supplement. — eTable 1. Algorithm to Identify Outpatient Episodes in Claims Data from 2008 to 2017 eTable 2. Results from Multivariable Difference-in-Differences Analyses Comparing Patients Undergoing SG vs RYGB Between 2008 and 2016 Across Categories of Ambulatory Care Costs and Use With a Gamma Distribution eTable 3. E-values and Interpretations for All Models with Statistically Significant Findings Comparing Patients Undergoing SG vs RYGB Between 2008 and 2016 eFigure. Flow Diagram for Cohort Selection of Patients with Index SG and RYGB Between 2008 and 2016 eTable 4. Attrition at Each Postoperative Year for Patients Undergoing SG or RYGB Between 2008 and 2016 eTable 5. Examining for Differential Attrition by Patient Characteristics: Baseline Characteristics of Patients in Main Analytic Cohort Enrolled at 1 and 4 Years After Surgery eTable 6. Presurgery Characteristics of the Matched Main Cohort vs the Sensitivity Cohort of Patients With Index SG and RYGB Between 2008 and 2016 eTable 7. Results From Multivariable Difference-in-Differences Analyses Comparing Patients Undergoing SG vs RYGB Between 2008 and 2016 Across Categories of Ambulatory Care Costs and Use Among Those With 4 Years of Follow-up eTable 8. Results From Multivariable Difference-in-Differences Analyses Comparing Patients Undergoing SG v. RYGB Between 2008 and 2016 Across Suboutcomes of Ambulatory Care Costs and Use Among Those With 4 Years of Follow-Up eTable 9. Stata Outputs for All Models eTable 10. Final Time Series Model After Backward Variable Selection for the Differenced Outcomes Between SG and RYBG eReferences. [file jamanetwopen-e229661-s001.pdf]

Supplemental Online Content

Lewis KH, Argetsinger S, Arterburn DE, et al. Comparison of ambulatory health care costs and use associated with Roux-en-Y gastric bypass vs sleeve gastrectomy. *JAMA Netw Open.* 2022;5(5):e229661. doi:10.1001/jamanetworkopen.2022.9661

eTable 1. Algorithm to Identify Outpatient Episodes in Claims Data from 2008 to 2017

eTable 2. Results from Multivariable Difference-in-Differences Analyses Comparing Patients Undergoing SG vs RYGB Between 2008 and 2016 Across Categories of Ambulatory Care Costs and Use With a Gamma Distribution

eTable 3. E-values and Interpretations for All Models with Statistically Significant Findings Comparing Patients Undergoing SG vs RYGB Between 2008 and 2016

eFigure. Flow Diagram for Cohort Selection of Patients with Index SG and RYGB Between 2008 and 2016

eTable 4. Attrition at Each Postoperative Year for Patients Undergoing SG or RYGB Between 2008 and 2016

eTable 5. Examining for Differential Attrition by Patient Characteristics: Baseline Characteristics of Patients in Main Analytic Cohort Enrolled at 1 and 4 Years After Surgery

eTable 6. Presurgery Characteristics of the Matched Main Cohort vs the Sensitivity Cohort of Patients With Index SG and RYGB Between 2008 and 2016

eTable 7. Results From Multivariable Difference-in-Differences Analyses Comparing Patients Undergoing SG vs RYGB Between 2008 and 2016 Across Categories of Ambulatory Care Costs and Use Among Those With 4 Years of Follow-up

eTable 8. Results From Multivariable Difference-in-Differences Analyses Comparing Patients Undergoing SG v. RYGB Between 2008 and 2016 Across Suboutcomes of Ambulatory Care Costs and Use Among Those With 4 Years of Follow-Up

eTable 9. Stata Outputs for All Models

eTable 10. Final Time Series Model After Backward Variable Selection for the Differenced Outcomes Between SG and RYBG

eReferences.

This supplemental material has been provided by the authors to give readers additional information about their work.

© 2022 Lewis KH et al. *JAMA Network Open.*

eTable 1. Algorithm to Identify Outpatient Episodes in Claims Data from 2008 to 2017	
Outpatient Episodes	The following logic was used to identify outpatient episodes in claims data[a]:<br>1. We first restricted to claims that were not flagged as Emergency Department visits, Inpatient stays or Observation stays<br>2. To define Office Visits:<br>a. Narrowed down to claims that were flagged as either an office visit or face to face visit<br>i. Office visits include claims with the following CPT codes: 99201-99205, 99211-99215, 99241-99245, 99271-99275, 99321-99322, 99331-99333, 99341-99353, 99381-99387, 99391-99397, 99401-99404, 99411-99412, 99429, 99455-99456, 99483, 99495-99496<br>ii. Face to face visits include claims with the following CPT codes: 99201-99215, 99217-99239, 99241-99255, 99281-99288, 99291-99292, 99304-99318, 99324-99337, 99339-99350, 99354-99429, 99441-99458, 99460-99463-99499<br>b. Office visits were separated into two subcategories, primary care provider (PCP) visits and specialist visits<br>c. Categorized PCP visits as those that were flagged within a PROVCAT category of any PCP, adult PCP or pediatric PCP<br>d. Categorized specialist visits as those that were flagged within a PROVCAT category of specialist or outpatient specialist<br>3. To define Laboratory visits:<br>a. Claims with CPT codes 80047-89398 were categorized as Laboratory<br>b. Pathology labs were excluded<br>c. The remaining laboratory visits were separated into two subcategories, nutrition labs (e.g. iron, copper, vitamin B12, vitamin D) and all other labs (e.g. hematology, chemistry, microbiology, toxicology, endocrine) by a clinician on the team<br>4. To define Radiology visits<br>a. Claims with CPT codes 70010-79999 were categorized as Radiology<br>b. Radiology visits were separated by body site imaged into abdomen/pelvic imaging and all other imaging based on review of each procedure code<br>5. To define Pharmacy claims<br>a. Pharmacy claims were separated into two subcategories, cardiometabolic prescription fills (including medications for T2D, hypertension, dyslipidemia) and all other prescription fill indications (including pain, mental illness, antimicrobials, etc.)<br>6. The remaining outpatient claims were categorized as Other and included claims for ambulatory procedures, durable medical equipment, vaccination, transport, facility-administered medications, and physical therapy/occupational therapy.
eTable 1: Algorithm to Identify Outpatient Episodes in Claims Data from 2008-2017<br>a. Data were housed at the Harvard Pilgrim Health Care Institute and accessed by local team members using data security and credentialing processes in accordance with the data use agreement and local regulatory guidelines.	

eTable 2. Results from Multivariable Difference-in-Differences Analyses Comparing Patients Undergoing SG vs RYGB Between 2008 and 2016 Across Categories of Ambulatory Care Costs and Use With a Gamma Distribution

	Post-Operative Year 1, SG vs. RYGB		Post-Operative Year 2, SG vs. RYGB		Post-Operative Year 3, SG vs. RYGB		Post-Operative Year 4, SG vs. RYGB		Post-Operative Year 1, SG vs. RYGB, gamma distribution		Post-Operative Year 2, SG vs. RYGB, gamma distribution		Post-Operative Year 3, SG vs. RYGB, gamma distribution		Post-Operative Year 4, SG vs. RYGB, gamma distribution	
	Absolute Difference[b] ($) (95% CI)	Relative Difference[c] (%) (95% CI)	Absolute Difference ($) (95% CI)	Relative Difference (%) (95% CI)	Absolute Difference[b] ($) (95% CI)	Relative Difference[c] (%) (95% CI)	Absolute Difference ($) (95% CI)	Relative Difference (%) (95% CI)	Absolute Difference (95% CI)	Relative Difference (%) (95% CI)	Absolute Difference (95% CI)	Relative Difference (%) (95% CI)	Absolute Difference (95% CI)	Relative Difference (%) (95% CI)	Absolute Difference (95% CI)	Relative Difference (%) (95% CI)
Total Ambulatory Care Costs	-421.9 (-1334.1, 490.4)	-5.3% (-16.1%, 5.6%)	-642.9 (-1643.4, 357.5)	-8.4% (-20.5%, 3.7%)	-138.9 (-1273.2, 995.4)	-2.0% (-17.8%, 13.9%)	382.8 (-1366.0, 2131.6)	5.1% (-19.2%, 29.5%)	-425.8 (-1347.7, 496.0)	-5.3% (-16.1%, 5.6%)	-647.9 (-1661.0, 365.2)	-8.4% (-20.6%, 3.8%)	-140.9 (-1289.3, 1007.5)	-2.0% (-17.9%, 13.9%)	383.6 (-1379.5, 2146.6)	5.1% (-19.3%, 29.4%)
Prescription Drug Costs	36.1 (-252.0, 324.3)	1.7% (-11.9%, 15.2%)	173.3 (-80.1, 426.7)	9.7% (-5.6%, 25.1%)	140.2 (-283.0, 563.3)	7.1% (-15.6%, 29.9%)	852.8[f] (395.6, 1310.0)	52.4%[e] (17.8%, 87.0%)	36.5 (-259.9, 332.9)	1.7% (-12.1%, 15.4%)	175.9 (-86.0, 437.7)	9.8% (-5.9%, 25.4%)	142.6 (-288.5, 573.7)	7.2% (-15.7%, 30.1%)	861.1[f] (392.9, 1329.3)	52.2%[e] (17.0%, 87.4%)
Office Visit Costs	-36.7 (-103.5, 30.1)	-4.1% (-11.2%, 3.1%)	-14.4 (-85.4, 56.5)	-1.7% (-10.1%, 6.6%)	-2.0 (-87.1, 83.1)	-0.3% (-11.1%, 10.6%)	-43.8 (-176.1, 88.5)	-5.3% (-20.5%, 10.0%)	-36.8 (-107.1, 33.4)	-4.1% (-11.6%, 3.4%)	-14.4 (-86.9, 58.1)	-1.7% (-10.2%, 6.8%)	-2.1 (-89.3, 85.2)	-0.3% (-11.3%, 10.8%)	-43.9 (-177.7, 89.8)	-5.3% (-20.6%, 10.1%)
Laboratory Costs	-118.9[d] (-220.2, -17.5)	-13.9%[e] (-24.2%, -3.6%)	-107.6[d] (-197.2, -18.1)	-15.9%[e] (-27.4%, -4.5%)	-106.7 (-244.5, 31.1)	-18.0% (-37.5%, 1.4%)	-4.0 (-116.7, 108.7)	-0.8% (-23.6%, 22.0%)	-118.9[d] (-222.7, -15.1)	-13.8%[d] (-24.4%, -3.3%)	-107.6[d] (-200.4, -14.9)	-15.9%[e] (-27.7%, -4.1%)	-106.9 (-246.4, 32.6)	-18.0% (-37.6%, 1.6%)	-3.9 (-117.9, 110.1)	-0.8% (-23.7%, 22.2%)
Radiology Costs	-4.6 (-97.7, 88.5)	-0.7% (-15.5%, 14.1%)	-63.6 (-189.7, 62.6)	-9.2% (-26.0%, 7.6%)	6.7 (-121.7, 135.0)	1.1% (-20.3%, 22.5%)	22.5 (-150.8, 195.7)	3.4% (-23.7%, 30.6%)	-4.7 (-100.5, 91.1)	-0.7% (-15.9%, 14.4%)	-63.9 (-190.3, 62.5)	-9.2% (-25.9%, 7.5%)	6.6 (-123.4, 136.6)	1.1% (-20.4%, 22.6%)	22.7 (-151.4, 196.8)	3.4% (-23.6%, 30.5%)
All Other Outpatient Costs	-264.7 (-945.4, 416.1)	-7.7% (-26.1%, 10.7)	-600.9 (-1432.8, 231.0)	-16.4% (-35.7%, 3.0%)	-164.6 (-1103.8, 774.6)	-5.1% (-33.2%, 22.9%)	-537.1 (-1900.9, 826.7)	-13.9% (-45.1%, 17.2%)	-264.7 (-933.7, 404.4)	-7.6% (-25.7%, 10.4%)	-600.9 (-1447.3, 245.5)	-16.3% (-35.9%, 3.4%)	-165.4 (-1104.6, 773.8)	-5.1% (-33.0%, 22.7%)	-538.9 (-1909.4, 831.6)	-13.9% (-45.1%, 17.2%)

eTable 2: Results from Multivariable Difference-in-Differences Analysesa Comparing Patients Undergoing SG vs. RYGB Between 2008-2016 Across Categories of Ambulatory Care Costs & Use with a Gamma Distribution

a. Difference-in-Differences analyses adjusting for all matched variables were used to generate between-group estimates for change in each outcome, at each time period, relative to a preoperative baseline period spanning months -24 to -13 before the index surgical date. We selected year -2 as the preoperative baseline for comparison because the year immediately before surgery represents a very high utilization time, due to pre-operative workup.

b. Absolute differences in each period refer to the estimated actual change in costs among SG patients minus that in RYGB patients in that segment versus baseline (months -24 to -13), accounting for all other periods.

c. Relative differences in each period refer to the estimated relative difference between SG and RYGB patients versus baseline, accounting for all other periods.

d. p-value <0.05

e. p-value <0.01

f. p-value <0.001

© 2022 Lewis KH et al. *JAMA Network Open.*

eTable 3. E-values and Interpretations for All Models with Statistically Significant Findings Comparing Patients Undergoing SG vs RYGB Between 2008 and 2016			
**Model**	**Cohort**	**E-value (E-value for relevant limit of confidence interval)[a]**	**Parameter Estimate from Model[b] (95% CI)**
Year 1 Laboratory Costs	Main cohort	1.54 (1.06)	0.88 (0.79 , 0.99)
Year 4 Prescription Costs	Main cohort	2.22 (1.55)	1.44 (1.14 , 1.80)
Year 3 Prescription Costs	4-year sensitivity cohort	1.87 (1.15)	1.28 (1.02, 1.59)
Year 4 Prescription Costs	4-year sensitivity cohort	2.11 (1.35)	1.38 (1.07 , 1.78)
Year 2 Encounters for cardiometabolic prescription fills	Main cohort	1.51 (1.11)	1.13 (1.01, 1.26)
Year 3 Encounters for cardiometabolic prescription fills	Main cohort	1.61 (1.14)	1.17 (1.01 , 1.34)
Year 4 Encounters for cardiometabolic prescription fills	Main cohort	1.82 (1.29)	1.26 (1.05 , 1.50)
Year 1 Other lab encounters	Main cohort	1.49 (1.17)	0.89 (0.81, 0.97)
Year 2 Other lab encounters	Main cohort	1.52 (1.11)	0.88 (0.79 , 0.99)
Year 3 Specialist encounters	Main cohort	1.50 (1.09)	0.89 (0.79 , 0.99)
Year 4 PCP encounters	4-year sensitivity cohort	1.58 (1.12)	1.15 (1.01 , 1.31)

eTable 3: E-values and Interpretations for All Models with Statistically-Significant Findings Comparing Patients Undergoing SG vs. RYGB Between 2008-2016

a. E-values were calculated using the E-Value package in R.[1–3] They can be interpreted as follows, using year 4 prescription costs as an example: For unmeasured confounding to invalidate the observed IRR of 1.4, a confounder would have to have a magnitude of effect greater than an IRR of 2.2. One of the most likely confounders, which we do match on and adjust for is ACG score (overall comorbidity measure). In our models, the largest IRR for that term was 2.16, which is slightly less than but similar than 2.2. It is unlikely there is another unmeasured confounder with a larger effect on prescription costs than overall comorbidity burden that would invalidate our results.

b. Since the e-value program requires the inputs to be RR or OR, we used incident rate ratio (IRR). IRRs are the exponentials of the corresponding regression coefficients in the Stata regression outputs.

© 2022 Lewis KH et al. *JAMA Network Open.*

eFigure. Flow Diagram for Cohort Selection of Patients with Index SG and RYGB Between 2008 and 2016

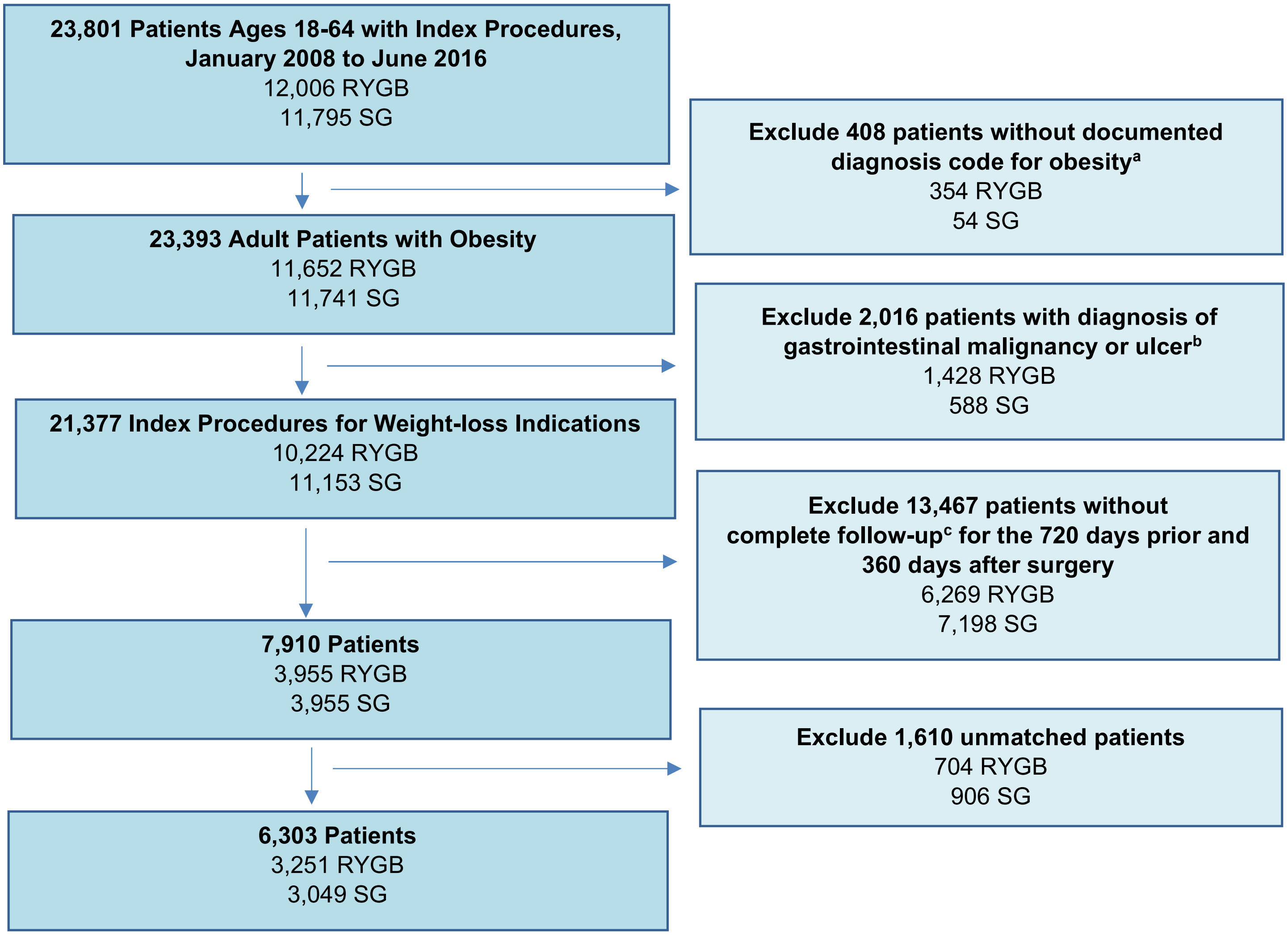

Abbreviations: RYGB, Roux-en-Y gastric bypass, SG, Sleeve gastrectomy

Legend Text: **a**. Based on the presence of either a specific BMI diagnosis > 30kg/m^2 or a nonspecific morbid obesity diagnosis in the 360 days before surgery.

b. Patients were excluded if they had a gastrointestinal malignancy diagnosis in the 720 days prior or a gastrointestinal ulcer diagnosis or procedure in the 30 days prior to their index surgery. **c.** Patients were censored when they turned 65, in the last month of our dataset (June 1, 2017), on the date of incident gastrointestinal malignancy or at insurance disenrollment (loss to follow-up).

© 2022 Lewis KH et al. *JAMA Network Open.*

eTable 4. Attrition at Each Postoperative Year for Patients Undergoing SG or RYGB Between 2008 and 2016

	**Time Since Surgery**			
	**1 year**	**2 years**	**3 years**	**4 years**
Number of patients observed by group, regardless of calendar year of surgery (numerators for all percent observed calculations)	SG 3049<br>RYGB 3251	SG 1952<br>RYGB 2173	SG 1054<br>RYGB 1151	SG 514<br>RYGB 643
Percent of full analytic cohort observed, relative to surgery date (using 3049 SG & 3251 RYGB as denominator)	SG 100%<br>RYGB 100%	SG 64%<br>RYGB 67%	SG 35%<br>RYGB 35%	SG 17%<br>RYGB 20%
Number of patients eligible for this amount of follow-up based on year of surgery	SG 3049<br>RYGB 3251	SG 2561<br>RYGB 2997	SG 1972<br>RYGB 2237	SG 1396<br>RYGB 1700
Percent of eligible patients observed (true cohort retention rate, using # eligible as denominator)	SG 100%<br>RYGB 100%	SG 76%<br>RYGB 73%	SG 53%<br>RYGB 51%	SG 37%<br>RYGB 38%

© 2022 Lewis KH et al. *JAMA Network Open.*

eTable 5. Examining for Differential Attrition by Patient Characteristics: Baseline Characteristics of Patients in Main Analytic Cohort Enrolled at 1 and 4 Years After Surgery

Variable[c]	**Enrolled at 360d After Matching[a], No. (%)**			**Enrolled at 1440d After Matching[a], No. (%)**		
	**RYGB (N=4488)**	**SG (N=4142)**	**Standardized Difference[b]**	**RYGB (N=687)**	**SG (N=392)**	**Standardized Difference[b]**
Year of Surgery			0.00			0.00
2008-2011	962 (21.4%)	888 (21.4%)		386 (56.1%)	220 (56.1%)	
2012-2014	2424 (54.0%)	2237 (54.0%)		301 (43.9%)	172 (43.9%)	
2015-2017	1102 (24.6%)	1017 (24.6%)				
Age ≥ 40 y	2985 (66.5%)	2821 (68.1%)	0.07	500 (72.8%)	293 (74.7%)	0.03
Female Sex	3449 (76.9%)	3163 (76.4%)	-0.01	535 (77.9%)	285 (72.7%)	-0.12
White Neighborhood[d], ≥75%	2436 (54.3%)	2165 (52.3%)	0.10	386 (56.1%)	217 (55.4%)	0.25
Neighborhood Poverty[e]			0.03			0.12
Less Poor (<10%)	2084 (46.5%)	1991 (48.1%)		357 (52.0%)	205 (52.2%)	
More Poor (≥10%)	2403 (53.5%)	2151 (51.9%)		330 (48.0%)	187 (47.7%)	
Region of United States			0.00			0.00
West	747 (16.6%)	689 (16.6%)		123 (17.9%)	70 (17.9%)	
South	2537 (56.5%)	2341 (56.5%)		389 (56.6%)	222 (56.6%)	
Midwest	862 (19.2%)	796 (19.2%)		138 (20.2%)	79 (20.2%)	
Northeast	342 (7.6%)	316 (7.6%)		37 (5.4%)	21 (5.4%)	
BMI Category[f]			0.00			0.00
30-39.9	612 (13.6%)	565 (13.6%)		77 (11.2%)	44 (11.2%)	
40-49.9	2579 (57.5%)	2380 (57.5%)		485 (70.7%)	277 (70.7%)	
50-59.9	614 (13.7%)	567 (13.7%)		56 (8.2%)	32 (8.2%)	
≥60	87 (1.9%)	80 (1.9%)		5 (0.8%)	3 (0.8%)	
Non-specific obesity	596 (13.3%)	550 (13.3%)		63 (9.2%)	36 (9.2%)	
ACG Score ≥3	1583 (35.3%)	1410 (34.0%)	-0.03	201 (29.3%)	115 (29.3%)	0.01
Type 2 Diabetes	1371 (30.5%)	1265 (30.5%)	0.00	182 (26.5%)	104 (26.5%)	0.00
Insulin Use	230 (5.1%)	212 (5.1%)	0.00	26 (3.8%)	15 (3.8%)	0.00

© 2022 Lewis KH et al. *JAMA Network Open.*

Hypertension	2533 (56.4%)	2405 (58.1%)	0.03	373 (54.3%)	228 (58.2%)	0.08
Cardiovascular Disease	445 (9.9%)	436 (10.5%)	0.02	64 (9.3%)	51 (13.0%)	0.12
Psychiatric Illness	1168 (26.0%)	1030 (24.9%)	-0.03	157 (22.9%)	85 (21.7%)	-0.03

eTable 5: Examining for Differential Attrition by Patient Characteristics: Baseline Characteristics of Patients in our Main Analytic Cohort Enrolled at 1 and 4 years after Surgery

Abbreviations: RYGB, Roux-en-Y gastric bypass, SG, Sleeve gastrectomy, BMI, Body Mass Index, ACG, Johns Hopkins Adjusted Clinical Groups ® System , ED, Emergency Department

a. We conducted coarsened exact matching (CEM) on BMI category, diabetes status and insulin use, total ambulatory care cost quartile during the pre-surgical year, region of the U.S., and calendar period, as well as tertile of a propensity score that included patient sex, age group, ACG score (<3 vs. ≥3), and neighborhood demographics b. Standardized differences are the difference in means between intervention and control divided by the SD of the difference in means. Lower absolute values indicate greater similarity, and values <0.2 indicate minimal differences between groups. c. Please refer to the methods section for complete descriptions of how we constructed baseline variables. d. White neighborhoods defined as census tracts where >75% of residents were Non-Hispanic White. e. Neighborhoods with more poverty were those where ≥10% of households were below the poverty line. f. Body mass index based on most recent pre-surgery diagnosis

© 2022 Lewis KH et al. *JAMA Network Open.*

eTable 6. Presurgery Characteristics of the Matched Main Cohort vs the Sensitivity Cohort of Patients With Index SG and RYGB Between 2008 and 2016

Variable[c]	RYGB, No. (%)			SG, No. (%)		
	**RYGB Main Cohort (N=3251)**	**RYGB Sensitivity Cohort (N=687)**	**Standardized Difference[b]**	**SG Main Cohort (N=3049)**	**SG Sensitivity Cohort (N=392)**	**Standardized Difference[b]**
Year of Surgery			0.53			0.83
2008-2011	2191 (67.4%)	568 (82.7%)		695 (22.8%)	220 (56.1%)	
2012-2014	881 (27.1%)	119 (17.3%)		1648 (54.1%)	172 (43.9%)	
2015-2017	179 (5.5%)	0 (0%)		706 (23.1%)	0 (0.0%)	
Age ≥ 40 y	2272 (69.9%)	470 (68.4%)	0.17	2140 (70.2%)	293 (74.7%)	0.14
Female Sex	2530 (77.8%)	534 (77.7%)	-0.02	2302 (75.5%)	285 (72.7%)	-0.07
White Neighborhood[d], ≥75%	1755 (54.0%)	375 (54.6%)	0.05	1601 (52.5%)	217 (55.4%)	0.15
Neighborhood Poverty[e]			0.12			0.12
Less Poor (<10%)	1491 (45.8%)	321 (46.7%)		1507 (49.4%)	205 (52.3%)	
More Poor (≥10%)	1760 (54.2%)	366 (53.3%)		1542 (50.6%)	187 (47.7%)	
Region of United States			0.06			0.20
West	588 (18.1%)	122 (17.8%)		489 (16.0%)	70 (17.9%)	
South	1749 (53.8%)	390 (56.8%)		1722 (56.5%)	222 (56.6%)	
Midwest	685 (21.1%)	138 (20.1%)		575 (18.9%)	79 (20.2%)	
Northeast	229 (7.0%)	37 (5.4%)		263 (8.6%)	21 (5.4%)	
BMI Category[f]			0.66			0.32
30-39.9	264 (8.1%)	46 (6.7%)		397 (13.0%)	44 (11.2%)	
40-49.9	1807 (55.6%)	520 (75.7%)		1824 (59.8%)	277 (70.7%)	
50-59.9	274 (8.4%)	40 (5.8%)		411 (13.5%)	32 (8.2%)	
≥60	31 (1.0%)	2 (0.3%)		35 (1.1%)	3 (0.8%)	
Non-specific obesity	875 (26.9%)	79 (11.5%)		382 (12.5%)	36 (9.2%)	
ACG Score ≥3	1152 (35.4%)	208 (30.3%)	0.00	1063 (34.9%)	115 (29.3%)	-0.12
Type 2 Diabetes	1267 (39.0%)	197 (28.7%)	0.00	947 (31.1%)	104 (26.5%)	-0.14
Insulin Use	274 (8.4%)	23 (3.3%)	0.00	159 (5.2%)	15 (3.8%)	-0.08

© 2022 Lewis KH et al. *JAMA Network Open.*

Hypertension	1987 (61.1%)	374 (54.4%)	0.00.	1795 (58.9%)	228 (58.2%)	-0.02
Cardiovascular Disease	408 (12.5%)	76 (11.1%)	0.17	346 (11.3%)	51 (13.0%)	0.05
Psychiatric Illness	843 (25.9%)	136 (19.8%)	0.00	738 (24.2%)	85 (21.7%)	0.18

eTable 6: Pre-Surgery Characteristics of the Matched Main Cohort vs. the Sensitivity Cohort of Patients with Index SG and RYGB between 2008 and 2016

Abbreviations: RYGB, Roux-en-Y gastric bypass, SG, Sleeve gastrectomy, BMI, Body Mass Index, ACG, Johns Hopkins Adjusted Clinical Groups ® System , ED, Emergency Department

a. We conducted coarsened exact matching (CEM) on BMI category, diabetes status and insulin use, total ambulatory care cost quartile during the pre-surgical year, region of the U.S., and calendar period, as well as tertile of a propensity score that included patient sex, age group, ACG score (<3 vs. ≥3), and neighborhood demographics. The comparison between the matched main cohort and sensitivity cohort did not use the matched weights because the coarsened exact matches were conducted separately. b. Standardized differences are the difference in means between intervention and control divided by the SD of the difference in means. Lower absolute values indicate greater similarity, and values <0.2 indicate minimal differences between groups. c. Please refer to the methods section for complete descriptions of how we constructed baseline variables. d. White neighborhoods defined as census tracts where >75% of residents were Non-Hispanic White. e. Neighborhoods with more poverty were those where ≥10% of households were below the poverty line. f. Body mass index based on most recent pre-surgery diagnosis

© 2022 Lewis KH et al. *JAMA Network Open.*

eTable 7. Results From Multivariable Difference-in-Differences Analyses Comparing Patients Undergoing SG vs RYGB Between 2008 and 2016 Across Categories of Ambulatory Care Costs and Use Among Those With 4 Years of Follow-up								
	Post-Operative Year 1, SG vs. RYGB		Post-Operative Year 2, SG vs. RYGB		Post-Operative Year 3, SG vs. RYGB		Post-Operative Year 4, SG vs. RYGB	
	Absolute Difference[b] (95% CI)	Relative Difference[c] (%) (95% CI)	Absolute Difference (95% CI)	Relative Difference (%) (95% CI)	Absolute Difference (95% CI)	Relative Difference (%) (95% CI)	Absolute Difference (95% CI)	Relative Difference (%) (95% CI)
Total Ambulatory Care Costs	1045.1 (-381.0, 2471.2)	14.6% (-7.2%, 36.4%)	705.6 (-557.3, 1968.4)	11.3% (-10.5%, 33.1%)	296.8 (-972.5, 1566.0)	4.7% (-16.3%, 25.7%)	1278.4 (-253.6, 2810.3)	18.6% (-6.4%, 43.7%)
Prescription Drug Costs	298.5 (-85.2, 682.2)	17.1% (-7.5%, 41.7%)	316.0 (-100.4, 732.4)	18.6% (-9.0%, 46.2%)	482.2[d] (87.8, 876.6)	29.6%[d] (1.0%, 58.3%)	700.2[e] (210.9, 1189.4)	42.5%[d] (6.9%, 78.0%)
Office Visit Costs	46.1 (-86.3, 178.5)	5.7% (-11.6%, 23.0%)	28.0 (-92.4, 148.3)	3.9% (-13.5%, 21.3%)	-14.7 (-130.0, 100.5)	-2.1% (-18.5%, 14.2%)	22.3 (-95.7, 140.4)	3.0% (-13.4%, 19.5%)
Laboratory Costs	-68.5 (-254.0, 117.1)	-7.9% (-28.0%, 12.1%)	-39.8 (-183.0, 103.5)	-6.5% (-28.8%, 15.8%)	-16.4 (-141.0, 108.2)	-3.3% (-27.9%, 21.3%)	47.0 (-82.3, 176.3)	10.0% (-19.2%, 39.2%)
Radiology Costs	180.7 (-12.1, 373.4)	33.3% (-9.8%, 76.5%)	92.7 (-130.1, 315.4)	15.7% (-26.3%, 57.7%)	-9.1 (-176.3, 158.1)	-1.7% (-32.9%, 29.5%)	147.1 (-38.1, 332.4)	27.8% (-13.8%, 69.4%)
All Other Outpatient Costs	246.5 (-1160.4, 1653.3)	7.0% (-35.0%, 49.0%)	152.9 (-989.2, 1295.0)	5.3% (-36.2%, 46.8%)	-333.7 (-1556.5, 889.0)	-10.5% (-45.6%, 24.7%)	-108.0 (-1572.6, 1356.5)	-2.9% (-40.7%, 35.0%)

eTable 7: Results from Multivariable Difference-in-Differences Analysesa Comparing Patients Undergoing SG vs. RYGB from 2008 to 2016 Across Categories of Ambulatory Care Costs & Use among the people with 4 years of follow-up

a. Difference-in-Differences analyses adjusting for all matched variables were used to generate between-group estimates for change in each outcome, at each time period, relative to a preoperative baseline period spanning months -24 to -13 before the index surgical date. We selected year -2 as the preoperative baseline for comparison because the year immediately before surgery represents a very high utilization time, due to pre-operative workup.

b. Absolute differences in each period refer to the estimated actual change in costs among SG patients minus that in RYGB patients in that segment versus baseline (months -24 to -13), accounting for all other periods.

c. Relative differences in each period refer to the estimated relative difference between SG and RYGB patients versus baseline, accounting for all other periods.

d. p-value <0.05

e. p-value <0.01

© 2022 Lewis KH et al. *JAMA Network Open.*

eTable 8. Results From Multivariable Difference-in-Differences Analyses Comparing Patients Undergoing SG v. RYGB Between 2008 and 2016 Across Suboutcomes of Ambulatory Care Costs and Use Among Those With 4 Years of Follow-Up								
	Post-Operative Year 1, SG vs. RYGB		Post-Operative Year 2, SG vs. RYGB		Post-Operative Year 3, SG vs. RYGB		Post-Operative Year 4, SG vs. RYGB	
	Absolute Difference[b] (95% CI)	Relative Difference[c] (%) (95% CI)	Absolute Difference (95% CI)	Relative Difference (%) (95% CI)	Absolute Difference (95% CI)	Relative Difference (%) (95% CI)	Absolute Difference (95% CI)	Relative Difference (%) (95% CI)
**PRESCRIPTION ENCOUNTERS**								
Cardiometabolic prescription fills	0.7[f] (0.3, 1.1)	16.6%[e] (6.3%, 26.9%)	1.1[f] (0.7, 1.5)	31.0%[f] (16.6%, 45.5%)	1.5[f] (0.9, 2.1)	42.2%[f] (21.5%, 62.8%)	1.7[f] (0.8, 2.6)	42.5%[e] (13.7%, 71.2%)
All other prescription fills	-0.5 (-1.4, 0.5)	-3.0% (-9.0%, 2.9%)	-0.1 (-1.2, 0.9)	-1.0% (-8.3%, 6.3%)	0.4 (-1.0, 1.9)	2.8% (-7.2%, 12.7%)	1.4 (-0.5, 3.3)	10.1% (-4.5%, 24.8%)
**OFFICE VISIT ENCOUNTERS**								
Specialist visits	0.0 (-0.6, 0.5)	-1.1% (-17.4%, 15.2%)	-0.1 (-0.6, 0.3)	-4.9% (-22.9%, 13.0%)	-0.2 (-0.6, 0.2)	-10.1% (-27.3%, 7.2%)	0.1 (-0.2, 0.5)	5.9% (-11.6%, 23.5%)
PCP visits	0.2 (-0.1, 0.4)	8.8% (-4.3%, 21.9%)	0.1 (-0.1, 0.4)	8.9% (-5.0%, 22.7%)	0.2 (-0.1, 0.4)	11.2% (-4.7%, 27.2%)	0.2 (0.0, 0.4)	15.5%[d] (0.2%, 30.8%)
**LABORATORY ENCOUNTERS**								
Nutrition labs	-1.2[d] (-2.2, -0.2)	-20.0%[e] (-34.0%, -6.0%)	-0.8[d] (-1.5, -0.2)	-24.3%[e] (-39.2%, -9.5%)	-0.5 (-1.0, 0.0)	-20.3%[d] (-38.3%, -2.3%)	-0.7[d] (-1.2, -0.1)	-30.4%[e] (-48.8%, -11.9%)
All other labs	-2.5[e] (-4.1, -1.0)	-14.5%[f] (-22.4%, -6.7%)	-2.5[e] (-4.1, -0.8)	-17.4%[f] (-27.0%, -7.7%)	-0.7 (-2.3, 0.9)	-5.7% (-18.7%, 7.4%)	-0.3 (-3.2, 2.6)	-2.3% (-26.3%, 21.6%)
**RADIOLOGY ENCOUNTERS**								
Abdomen/pelvic imaging	-0.2 (-0.4, 0.1)	-18.7% (-38.8%, 1.5%)	-0.2 (-0.5, 0.0)	-22.7%[d] (-44.6%, -0.8%)	-0.1 (-0.3, 0.1)	-14.4% (-44.3%, 15.5%)	-0.1 (-0.4, 0.1)	-19.7% (-51.6%, 12.1%)
All other imaging	0.1 (-0.3, 0.4)	2.7% (-10.2%, 15.6%)	0.2 (-0.2, 0.6)	6.2% (-8.5%, 20.9%)	0.4 (-0.1, 1.0)	14.1% (-6.3%, 34.6%)	0.3 (-0.3, 0.9)	9.0% (-11.5%, 29.5%)

eTable 8: Results from Multivariable Difference-in-Differences Analysesa Comparing Patients Undergoing SG vs. RYGB Between 2008-2016 Across Sub-Outcomes of Ambulatory Care Costs & Use Among the People With 4 Years of Follow-Up

a. Difference-in-Differences analyses adjusting for all matched variables were used to generate between-group estimates for change in each outcome, at each time period, relative to a preoperative baseline period spanning months -24 to -13 before the index surgical date. We selected year -2 as the preoperative baseline for comparison because the year immediately before surgery represents a very high utilization time, due to pre-operative workup.
b. Absolute differences in each period refer to the estimated actual change in costs among SG patients minus that in RYGB patients in that segment versus baseline (months -24 to -13), accounting for all other periods.
c. Relative differences in each period refer to the estimated relative difference between SG and RYGB patients versus baseline, accounting for all other periods.
d. p-value <0.05
e. p-value <0.01
f. p-value <0.001

<table>
<tr><td colspan="3">eTable 9. Stata Outputs for All Models[a]</td></tr>
<tr><td>Outcome</td><td>Main Cohort</td><td>Sensitivity Cohort</td></tr>
<tr><td>Total Ambulatory Care Costs</td><td>--
| Robust
tot_ambcst | Coef. Std. Err. z P>|z| [95% Conf. Interval]
-------------------------+--
tot_ambcst |
yr |
1 | .1300821 .0522103 2.49 0.013 .0277517 .2324124
2 | .1046842 .0587896 1.78 0.075 -
.0105413 .2199097
3 | .0366438 .0725058 0.51 0.613 -
.1054649 .1787526
4 | .1130782 .1034547 1.09 0.274 -
.0896893 .3158456
|
1.sg | .0351299 .0468238 0.75 0.453 -
.0566431 .1269029
|
sg#yr |
1 1 | -.0630903 .058405 -1.08 0.280 -.177562 .0513815
1 2 | -.0923825 .0675037 -1.37 0.171 -
.2246873 .0399224
1 3 | -.0308911 .0822194 -0.38 0.707 -
.1920381 .1302559
1 4 | .0271538 .1172427 0.23 0.817 -
.2026377 .2569452
|
age_group |
2 | .0868346 .0854009 1.02 0.309 -
.0805482 .2542173
3 | .156703 .0839819 1.87 0.062 -.0078985 .3213045
4 | .315266 .0858431 3.67 0.000 .1470166 .4835153
|
race |
2 | -.3271405 .0864463 -3.78 0.000 -.4965721 -
.1577089
3 | -.0635124 .0397032 -1.60 0.110 -
.1413293 .0143044
4 | -.0368569 .0681548 -0.54 0.589 -.1704378 .096724
5 | -.0889176 .1109678 -0.80 0.423 -
.3064105 .1285754
|
ed |
2 | .0009908 .0471978 0.02 0.983 -
.0915151 .0934968
3 | -.0412176 .0787256 -0.52 0.601 -.195517 .1130817
4 | -.2372242 .1080496 -2.20 0.028 -.4489975 -
.0254508</td><td>--

| Robust
tot_ambcst | Coef. Std. Err. z P>|z|
[95% Conf. Interval]
-------------------------+--
----
tot_ambcst |
yr |
1 | -.0677238 .0655575 -1.03 0.302 -
.1962142 .0607666
2 | -.194411 .0725319 -2.68 0.007 -
.3365709 -.0522511
3 | -.1760789 .0746916 -2.36 0.018 -
.3224717 -.0296861
4 | -.0872408 .079984 -1.09 0.275 -
.2440066 .069525
|
1.sg | .0552233 .0816563 0.68 0.499 -
.1048201 .2152666
|
sg#yr |
1 1 | .1358632 .0969947 1.40 0.161 -
.0542429 .3259692
1 2 | .1044227 .1005147 1.04 0.299 -
.0925825 .3014279
1 3 | .0333842 .1028376 0.32 0.745 -
.1681737 .2349421
1 4 | .167206 .1078096 1.55 0.121 -
.044097 .378509
|
age_group |
2 | .3106588 .1498167 2.07 0.038
.0170235 .6042941
3 | .4183738 .1377426 3.04 0.002
.1484032 .6883444
4 | .6154705 .1426143 4.32 0.000
.3359515 .8949894
|
race |
2 | .3230256 .2585262 1.25 0.211 -
.1836764 .8297276
3 | .0058771 .0783017 0.08 0.940 -
.1475913 .1593456
4 | .0553605 .1218815 0.45 0.650 -
.1835229 .2942439</td></tr>
</table>

© 2022 Lewis KH et al. *JAMA Network Open.*

<table>
<tr>
<td></td>
<td>|
pov |
2 | .0152685 .0553875 0.28 0.783 -.093289 .123826
3 | .013897 .0533524 0.26 0.794 -.0906717 .1184658
4 | .0780193 .0674812 1.16 0.248 -.0542414 .21028
|
1.acgge3
| .6108935 .0371825 16.43 0.000 .5380171 .6837699
1.female | .1497811 .0450562 3.32 0.001 .0614726 .2380896
1.hypertension | .2273961 .0391821 5.80 0.000 .1506006 .3041916
_cons | 8.137427 .1005821 80.90 0.000 7.94029 8.334564
-------------------------+--
inflate |
yr |
1 | -1.887072 .9063757 -2.08 0.037 -3.663536 -
.1106087
2 | .6073201 .3205366 1.89 0.058 -.0209202 1.23556
3 | 1.199078 .3175239 3.78 0.000 .5767421 1.821413
4 | 1.551541 .3264369 4.75 0.000 .9117366 2.191346
|
1.sg | .603472 .2281868 2.64 0.008 .1562342 1.05071
|
sg#yr |
1 1 | -.2027465 .9708919 -0.21 0.835 -
2.10566 1.700167
1 2 | -.3879155 .3539717 -1.10 0.273 -
1.081687 .3058563
1 3 | -.6284691 .354131 -1.77 0.076 -
1.322553 .0656149
1 4 | -.8756574 .3779266 -2.32 0.021 -1.61638 -
.1349348
|
age_group |
2 | -.2970284 .2389247 -1.24 0.214 -
.7653122 .1712553
3 | -.6345757 .2444575 -2.60 0.009 -1.113704 -
.1554477
4 | -.694009 .3759116 -1.85 0.065 -1.430782 .0427642
|
race |
2 | .3325408 .3742335 0.89 0.374 -
.4009434 1.066025
3 | .0611953 .270663 0.23 0.821 -.4692944 .591685
4 | -.1840912 .2436136 -0.76 0.450 -
.6615651 .2933827
5 | .6184974 .4217721 1.47 0.143 -
.2081608 1.445156
|
ed |
2 | -.2800716 .2134251 -1.31 0.189 -.698377 .1382339</td>
<td>5 | -.2810962 .4522778 -0.62 0.534 -
1.167544 .605352
|
ed |
2 | -.0780973 .0922551 -0.85 0.397 -
.258914 .1027193
3 | -.4125243 .1227459 -3.36 0.001 -
.6531019 -.1719467
4 | -.3874549 .1925998 -2.01 0.044 -
.7649436 -.0099661
|
pov |
2 | .0188421 .09851 0.19 0.848 -
.1742338 .2119181
3 | .2213879 .1087444 2.04 0.042
.0082529 .434523
4 | .2556868 .1273706 2.01 0.045
.0060451 .5053285
|
1.acgge3 | .6487832 .0793372 8.18 0.000
.4932852 .8042813
1.female | .291947 .0770203 3.79 0.000
.1409899 .4429041
1.hypertension | .2166843 .074549 2.91 0.004
.070571 .3627977
_cons | 7.744628 .1658379 46.70 0.000
7.419592 8.069665
-------------------------+--
----
inflate |
yr |
1 | -2.614939 .8624431 -3.03 0.002 -
4.305297 -.9245819
2 | -.6323648 .4651289 -1.36 0.174 -
1.544001 .279271
3 | .2695607 .4075011 0.66 0.508 -
.5291269 1.068248
4 | .0691787 .4136985 0.17 0.867 -
.7416554 .8800129
|
1.sg | .1177539 .4342191 0.27 0.786 -
.7332999 .9688078
|
sg#yr |
1 1 | .7559106 1.201746 0.63 0.529 -
1.599468 3.111289
1 2 | -.1134044 .6929083 -0.16 0.870 -
1.47148 1.244671
1 3 | -.5701252 .6080146 -0.94 0.348 -
1.761812 .6215616</td>
</tr>
</table>

© 2022 Lewis KH et al. *JAMA Network Open.*

```
                 3 |   -.02529   .3758617    -0.07   0.946    -.7619654    .7113853
                 4 |  .5431136   .4204263     1.29   0.196    -
.2809067    1.367134
                   |
               pov |
                 2 |  .1568016   .2188591     0.72   0.474    -
.2721544    .5857576
                 3 |  .4146625   .2540164     1.63   0.103    -
.0832005    .9125255
                 4 |  .2567472   .2567622     1.00   0.317    -
.2464975    .7599918
                   |
          1.acgge3 |  -.5785157   .2774364    -2.09   0.037    -1.122281   -.0347504
          1.female |  -.8606736   .1675068    -5.14   0.000    -1.188981   -.5323663
    1.hypertension |  -.5426591   .2207914    -2.46   0.014    -.9754023   -.1099158
             _cons |  -2.882227   .4346165    -6.63   0.000    -3.734059   -2.030394
-------------------------+----------------------------------------------------------------
          /lnalpha
|   .2142134   .0143763    14.90   0.000     .1860363    .2423904
-------------------------+----------------------------------------------------------------
             alpha |   1.238887   .0178106                      1.204466    1.274292
```

```
               1 4  |  -.1529369   .5812277    -0.26   0.792   -
1.292122    .9862486
                    |
          age_group |
                  2 |  -.7760431   .6082261    -1.28   0.202   -
1.968144    .4160581
                  3 |  -.5796573   .5877958    -0.99   0.324   -
1.731716    .5724014
                  4 |  -.5895225   .6761549    -0.87   0.383   -
1.914762    .7357168
                    |
               race |
                  2 |   -.388123   .8273418    -0.47   0.639   -
2.009683    1.233437
                  3 |  -.0379152    .311015    -0.12   0.903   -
.6474935     .571663
                  4 |   .3492366   .4033844     0.87   0.387   -
.4413822    1.139855
                  5 |   1.101736   1.118069     0.99   0.324   -
1.089639    3.293111
                    |
                 ed |
                  2 |     .64808   .4335967     1.49   0.135   -
.2017539    1.497914
                  3 |  -.0635765   .6639759    -0.10   0.924   -
1.364945    1.237792
                  4 |   1.910026   .7239774     2.64   0.008
.491056    3.328995
                    |
                pov |
                  2 |  -.3100367   .4216689    -0.74   0.462   -
1.136493    .5164192
                  3 |  -.5767908   .4289469    -1.34   0.179   -
1.417511    .2639296
                  4 |  -.5389637   .5822057    -0.93   0.355   -
1.680066    .6021385
                    |
           1.acgge3 |  -.2344092   .3537899    -0.66   0.508   -
.9278246    .4590063
           1.female |  -.9558854   .3003114    -3.18   0.001   -
1.544485   -.3672859
     1.hypertension |  -.5177437   .3231409    -1.60   0.109   -
1.151088    .1156009
              _cons |  -2.021291   .6928652    -2.92   0.004   -
3.379282   -.6633005
-------------------------+-------------------------------------------------
-------------
           /lnalpha |   .1619531   .0259081     6.25   0.000
.1111741    .2127322
```

© 2022 Lewis KH et al. *JAMA Network Open.*

<table>
<tr>
<td></td>
<td></td>
<td>-------------------------+---
----
alpha | 1.175805 .0304629
1.117589 1.237053

------------</td>
</tr>
<tr>
<td>Laboratory Costs</td>
<td>--
| Robust
lab_costs | Coef. Std. Err. z P>|z| [95% Conf. Interval]
-------------------------+--
lab_costs |
yr |
1 | .6543112 .0514338 12.72 0.000 .5535028 .7551195
2 | .510148 .0561491 9.09 0.000 .4000979 .6201982
3 | .4809451 .1080338 4.45 0.000 .2692028 .6926874
4 | .318169 .0880788 3.61 0.000 .1455377 .4908002
|
1.sg | .0844886 .052728 1.60 0.109 -.0188564 .1878335
|
sg#yr |
1 1 | -.119187 .0589063 -2.02 0.043 -.2346412 -.0037329
1 2 | -.1165968 .0677714 -1.72 0.085 -.2494263 .0162326
1 3 | -.1598809 .1179095 -1.36 0.175 -.3909792 .0712175
1 4 | .0129087 .1119906 0.12 0.908 -.2065888 .2324061
|
age_group |
2 | -.1240619 .1006061 -1.23 0.218 -.3212462 .0731225
3 | -.1730604 .1010661 -1.71 0.087 -.3711464 .0250255
4 | -.1375727 .1000431 -1.38 0.169 -.3336536 .0585082
|
race |
2 | -.126456 .0848695 -1.49 0.136 -.2927972 .0398852
3 | .0204672 .0419857 0.49 0.626 -.0618232 .1027577
4 | .0264334 .0543673 0.49 0.627 -.0801245 .1329913
5 | .0716158 .1621349 0.44 0.659 -.2461627 .3893944
|
ed |
2 | -.0022996 .0503296 -0.05 0.964 -.1009438 .0963446
3 | .0407735 .0673051 0.61 0.545 -.0911422 .1726892
4 | -.2027472 .1029343 -1.97 0.049 -.4044947 -.0009997
|
pov |
2 | .0859617 .0511911 1.68 0.093 -.014371 .1862943
3 | .0593773 .0487762 1.22 0.223 -.0362223 .1549769
4 | .0680626 .0627294 1.09 0.278 -.0548848 .1910099
|
1.acgge3 | .3573754 .0360761 9.91 0.000 .2866677 .4280832
1.female | .1927742 .0438478 4.40 0.000 .1068341 .2787142
1.hypertension | .0896938 .0363785 2.47 0.014 .0183933 .1609944
_cons | 5.788467 .1140478 50.75 0.000 5.564937 6.011996</td>
<td>--

| Robust
lab_costs | Coef. Std. Err. z P>|z| [95%
Conf. Interval]
-------------------------+---
------
lab_costs |
yr |
1 | .714406 .081109 8.81 0.000
.5554352 .8733767
2 | .4018225 .0885895 4.54 0.000
.2281902 .5754548
3 | .2961998 .0936746 3.16 0.002
.112601 .4797986
4 | .21744 .0922073 2.36 0.018
.036717 .3981629
|
1.sg | .196472 .0947578 2.07 0.038
.0107501 .3821939
|
sg#yr |
1 1 | -.0992007 .108356 -0.92 0.360 -
.3115746 .1131732
1 2 | -.0747695 .1203048 -0.62 0.534 -
.3105626 .1610237
1 3 | -.0796521 .1264009 -0.63 0.529 -
.3273934 .1680892
1 4 | .0609012 .1315611 0.46 0.643 -
.1969538 .3187561
|
age_group |
2 | .1532045 .2054688 0.75 0.456 -
.2495069 .5559159
3 | .114744 .1905635 0.60 0.547 -
.2587536 .4882416
4 | .2456324 .1898106 1.29 0.196 -
.1263895 .6176542
|
race |
2 | .4225373 .2742105 1.54 0.123 -
.1149054 .9599799
3 | .0922739 .085115 1.08 0.278 -
.0745485 .2590963</td>
</tr>
</table>

© 2022 Lewis KH et al. *JAMA Network Open.*

<table>
<tr>
<td></td>
<td>-------------------------+--
inflate |
yr |
1 | -1.146851 .178628 -6.42 0.000 -1.496956 -.7967466
2 | -.208396 .1376768 -1.51 0.130 -.4782376 .0614455
3 | .3453693 .1459005 2.37 0.018 .0594095 .6313291
4 | .4394253 .1517111 2.90 0.004 .142077 .7367736
|
1.sg | -.1692589 .1074407 -1.58 0.115 -.3798389 .0413211
|
sg#yr |
1 1 | .2096964 .1952363 1.07 0.283 -.1729596 .5923525
1 2 | .3567769 .1526478 2.34 0.019 .0575927 .655961
1 3 | .2192747 .1636082 1.34 0.180 -.1013916 .5399409
1 4 | .1422229 .1769418 0.80 0.422 -.2045765 .4890224
|
age_group |
2 | .1763324 .1401416 1.26 0.208 -.0983402 .451005
3 | .0448019 .1385184 0.32 0.746 -.2266892 .316293
4 | -.0877634 .1510705 -0.58 0.561 -.3838561 .2083294
|
race |
2 | .0061637 .1784682 0.03 0.972 -.3436276 .355955
3 | -.1659573 .0879081 -1.89 0.059 -.338254 .0063394
4 | -.0190746 .0970106 -0.20 0.844 -.2092119 .1710626
5 | -.1490092 .2585861 -0.58 0.564 -.6558286 .3578101
|
ed |
2 | .0035455 .0885094 0.04 0.968 -.1699297 .1770206
3 | .29987 .1300658 2.31 0.021 .0449457 .5547943
4 | .1540456 .2066279 0.75 0.456 -.2509376 .5590287
|
pov |
2 | .0525246 .0928111 0.57 0.571 -.1293817 .234431
3 | .1182521 .0997656 1.19 0.236 -.0772849 .313789
4 | -.0063422 .1183861 -0.05 0.957 -.2383748 .2256903
|
1.acgge3 | -.4205963 .079132 -5.32 0.000 -.5756921 -.2655005
1.female | -.6953134 .072172 -9.63 0.000 -.836768 -.5538588
1.hypertension | -.3545765 .0740219 -4.79 0.000 -.4996568 -.2094963
_cons | -.6874381 .1907888 -3.60 0.000 -1.061377 -.3134989
-------------------------+--
/lnalpha | .0997812 .016799 5.94 0.000 .0668558 .1327066
-------------------------+--
alpha | 1.104929 .0185617 1.069141 1.141915
--</td>
<td>4 | .0015828 .1230537 0.01 0.990 -
.2395981 .2427637
5 | -.5625088 .186767 -3.01 0.003 -
.9285654 -.1964523
|
ed |
2 | -.1094275 .1002343 -1.09 0.275 -
.3058831 .0870281
3 | -.4105837 .1505509 -2.73 0.006 -
.7056581 -.1155094
4 | -.2166796 .162916 -1.33 0.184 -
.5359891 .1026298
|
pov |
2 | .2127004 .0893527 2.38 0.017
.0375724 .3878285
3 | .4038998 .1089802 3.71 0.000
.1903026 .617497
4 | .4291443 .1188116 3.61 0.000
.1962778 .6620108
|
1.acgge3 | .3766673 .0651947 5.78 0.000
.2488881 .5044464
1.female | .3067246 .0750785 4.09 0.000
.1595735 .4538756
1.hypertension | .0905037 .0660813 1.37 0.171 -
.0390132 .2200207
_cons | 5.093253 .2230815 22.83 0.000
4.656021 5.530485
-------------------------+--
------
inflate |
yr |
1 | -.8811975 .212489 -4.15 0.000 -
1.297668 -.4647267
2 | -.4394802 .1991329 -2.21 0.027 -
.8297736 -.0491868
3 | .3219254 .2329782 1.38 0.167 -
.1347035 .7785543
4 | .1395711 .1957558 0.71 0.476 -
.2441033 .5232454
|
1.sg | .1007296 .1963253 0.51 0.608 -
.284061 .4855202
|
sg#yr |
1 1 | -.1264513 .3063801 -0.41 0.680 -
.7269453 .4740426
1 2 | -.0253249 .277608 -0.09 0.927 -
.5694265 .5187767</td>
</tr>
</table>

© 2022 Lewis KH et al. *JAMA Network Open.*

		1 3 | -.2830594 .2949007 -0.96 0.337 -.8610541 .2949353<br>1 4 | -.2295359 .2680038 -0.86 0.392 -.7548137 .2957418<br>| age_group | 2 | -.2858891 .2998832 -0.95 0.340 -.8736494 .3018712<br>3 | -.1010193 .2915515 -0.35 0.729 -.6724497 .4704111<br>4 | -.1890838 .313294 -0.60 0.546 -.8031288 .4249612<br>| race | 2 | -.1511814 .3090761 -0.49 0.625 -.7569595 .4545967<br>3 | -.0008067 .1468763 -0.01 0.996 -.2886789 .2870655<br>4 | .071084 .2127238 0.33 0.738 -.345847 .488015<br>5 | 1.350665 .445764 3.03 0.002 .4769836 2.224346<br>| ed | 2 | .023012 .1853796 0.12 0.901 -.3403255 .3863494<br>3 | .2584854 .2412624 1.07 0.284 -.2143801 .731351<br>4 | .4501678 .4974441 0.90 0.365 -.5248047 1.42514<br>| pov | 2 | -.2006706 .1753914 -1.14 0.253 -.5444314 .1430901<br>3 | -.1805451 .1678535 -1.08 0.282 -.5095319 .1484418<br>4 | .1935606 .2144063 0.90 0.367 -.2266681 .6137893<br>| 1.acgge3 | -.4815896 .1493939 -3.22 0.001 -.7743962 -.1887829<br>1.female | -.8324693 .1296967 -6.42 0.000 -1.08667 -.5782685<br>1.hypertension | -.243721 .1375548 -1.77 0.076 -.5133234 .0258814<br>_cons | -.6352278 .3352326 -1.89 0.058 -1.292272 .021816<br>-------------------------+--

© 2022 Lewis KH et al. *JAMA Network Open.*

<table>
<tr><td></td><td></td><td>/lnalpha | .0866247   .0358974   2.41   0.016
.0162672   .1569823
-------------------------+--
------
alpha | 1.090487   .0391456   1.0164
1.169975

-----</td></tr>
<tr><td>Office Visit Costs</td><td>--
| Robust
office_vst_costs | Coef.   Std. Err.   z   P>|z| [95% Conf. Interval]
-------------------------+--
office_vst_costs |
yr |
1 | .075219   .0340176   2.21   0.027   .0085457   .1418923
2 | .0480531   .0361069   1.33   0.183   -.0227152   .1188213
3 | .0331293   .0439789   0.75   0.451   -.0530678   .1193264
4 | .0907001   .0683672   1.33   0.185   -.0432972   .2246974
|
1.sg | .0073287   .0360242   0.20   0.839   -.0632774   .0779348
|
sg#yr |
1 1 | -.0417355   .0379619   -1.10   0.272   -.1161395   .0326685
1 2 | -.0085339   .0422667   -0.20   0.840   -.0913751   .0743074
1 3 | -.0050072   .0519745   -0.10   0.923   -.1068754   .096861
1 4 | -.0465237   .0792093   -0.59   0.557   -.2017711   .1087237
|
age_group |
2 | -.0359688   .0546167   -0.66   0.510   -.1430156   .071078
3 | -.0134491   .055215   -0.24   0.808   -.1216685   .0947703
4 | .0307841   .0539735   0.57   0.568   -.0750021   .1365703
|
race |
2 | -.1154   .0573267   -2.01   0.044   -.2277583   -.0030416
3 | .0238739   .0317576   0.75   0.452   -.0383699   .0861176
4 | -.0528301   .0446994   -1.18   0.237   -.1404393   .0347792
5 | .1921688   .1616374   1.19   0.234   -.1246347   .5089723
|
ed |
2 | -.1223388   .0338144   -3.62   0.000   -.1886138   -.0560639
3 | -.1077054   .0477258   -2.26   0.024   -.2012463   -.0141646
4 | -.2989755   .0923671   -3.24   0.001   -.4800116   -.1179393
|
pov |
2 | -.0085615   .0402488   -0.21   0.832   -.0874478   .0703248
3 | -.0148553   .0414081   -0.36   0.720   -.0960138   .0663032
4 | .0440425   .0485688   0.91   0.365   -.0511506   .1392356
|
1.acgge3 | .4346333   .0271146   16.03   0.000   .3814897   .4877769
1.female | .2120518   .0324733   6.53   0.000   .1484052   .2756983</td><td>---
-----
| Robust
office_vst_costs | Coef.   Std. Err.   z   P>|z| [95%
Conf. Interval]
-------------------------+--
------
office_vst_costs |
yr |
1 | -.0607606   .0654769   -0.93   0.353   -
.189093   .0675719
2 | -.1575532   .064687   -2.44   0.015   -
.2843375   -.030769
3 | -.171027   .0638627   -2.68   0.007   -
.2961956   -.0458584
4 | -.0911155   .0601077   -1.52   0.130   -
.2089245   .0266935
|
1.sg | -.0178674   .0652206   -0.27   0.784   -
.1456975   .1099626
|
sg#yr |
1 1 | .0667921   .081787   0.82   0.414   -
.0935075   .2270916
1 2 | .0512234   .0832348   0.62   0.538   -
.1119138   .2143607
1 3 | -.0190284   .0830361   -0.23   0.819   -
.1817761   .1437193
1 4 | .0175208   .0802448   0.22   0.827   -
.1397561   .1747976
|
age_group |
2 | .0977376   .1129564   0.87   0.387   -
.1236528   .319128
3 | .1559018   .104499   1.49   0.136   -
.0489125   .360716
4 | .2084051   .1071351   1.95   0.052   -
.0015757   .418386
|
race |
2 | .0981403   .2158659   0.45   0.649   -
.3249492   .5212298</td></tr>
</table>

© 2022 Lewis KH et al. *JAMA Network Open.*

<table>
<tr>
<td></td>
<td>1.hypertension | .1367799 .0288606 4.74 0.000 .0802143 .1933456
_cons | 6.352741 .0709954 89.48 0.000 6.213593 6.49189
-------------------------+--
inflate |
yr |
1 | -1.242991 .2900438 -4.29 0.000 -1.811466 -.6745152
2 | -.0765658 .170181 -0.45 0.653 -.4101144 .2569829
3 | .5250541 .2227645 2.36 0.018 .0884437 .9616646
4 | .5109197 .2230448 2.29 0.022 .07376 .9480795
|
1.sg | -.0096824 .1508411 -0.06 0.949 -.3053255 .2859606
|
sg#yr |
1 1 | -.0265249 .3211038 -0.08 0.934 -.6558769 .6028271
1 2 | .1295696 .1937029 0.67 0.504 -.2500811 .5092202
1 3 | -.0186387 .2430863 -0.08 0.939 -.495079 .4578016
1 4 | .0723802 .2530549 0.29 0.775 -.4235983 .5683588
|
age_group |
2 | .0581127 .1668062 0.35 0.728 -.2688215 .3850469
3 | -.2542378 .1726173 -1.47 0.141 -.5925614 .0840858
4 | -.238496 .2206506 -1.08 0.280 -.6709632 .1939713
|
race |
2 | .2561673 .2350851 1.09 0.276 -.2045911 .7169257
3 | -.0068203 .123037 -0.06 0.956 -.2479685 .2343278
4 | .1722662 .2075134 0.83 0.406 -.2344526 .578985
5 | .1930246 .318762 0.61 0.545 -.4317375 .8177867
|
ed |
2 | -.1120654 .1152337 -0.97 0.331 -.3379193 .1137884
3 | .1761718 .2360263 0.75 0.455 -.2864312 .6387749
4 | .0973318 .258735 0.38 0.707 -.4097795 .6044431
|
pov |
2 | .1318328 .1642655 0.80 0.422 -.1901218 .4537873
3 | .3337268 .163095 2.05 0.041 .0140664 .6533871
4 | .1493999 .1817465 0.82 0.411 -.2068167 .5056166
|
1.acgge3 | -.5400312 .1224451 -4.41 0.000 -.7800192 -.3000433
1.female | -.6752906 .1002319 -6.74 0.000 -.8717415 -.4788397
1.hypertension | -.3907099 .1134496 -3.44 0.001 -.613067 -.1683528
_cons | -1.680289 .2491992 -6.74 0.000 -2.168711 -1.191868
-------------------------+--
/lnalpha | -.4395678 .0171343 -25.65 0.000 -.4731504 -.4059853
-------------------------+--
alpha | .6443148 .0110399 .6230363 .66632
--</td>
<td>3 | .0618011 .0615591 1.00 0.315 -
.0588524 .1824547
4 | -.1277594 .0871183 -1.47 0.143 -
.2985081 .0429894
5 | -.2065822 .229941 -0.90 0.369 -
.6572583 .2440939
|
ed |
2 | -.1371049 .0709971 -1.93 0.053 -
.2762565 .0020468
3 | -.3566859 .0958916 -3.72 0.000 -
.5446299 -.1687418
4 | -.5391232 .1366557 -3.95 0.000 -
.8069635 -.2712829
|
pov |
2 | .0656184 .0826018 0.79 0.427 -
.0962781 .2275149
3 | .0811099 .0813158 1.00 0.319 -
.0782662 .2404859
4 | .2475778 .0954419 2.59 0.009
.0605152 .4346404
|
1.acgge3 | .4370788 .0631562 6.92 0.000
.3132949 .5608627
1.female | .3495951 .0561137 6.23 0.000
.2396143 .4595759
1.hypertension | .1225266 .0574756 2.13 0.033
.0098764 .2351768
_cons | 6.101667 .1317774 46.30 0.000
5.843388 6.359946
-------------------------+---
------
inflate |
yr |
1 | -1.40052 .3042071 -4.60 0.000 -
1.996755 -.8042848
2 | -.7259542 .2442252 -2.97 0.003 -
1.204627 -.2472816
3 | -.3423549 .2491203 -1.37 0.169 -
.8306218 .1459119
4 | -.027969 .2714447 -0.10 0.918 -
.5599909 .5040529
|
1.sg | -.0943531 .251862 -0.37 0.708 -
.5879935 .3992873
|
sg#yr |
1 1 | .2388709 .4575379 0.52 0.602 -
.6578869 1.135629</td>
</tr>
</table>

© 2022 Lewis KH et al. *JAMA Network Open.*

		1 2 | .2103389 .3709372 0.57 0.571 -<br>.5166847 .9373625<br>1 3 | .0094246 .3637818 0.03 0.979 -<br>.7035746 .7224238<br>1 4 | -.183647 .3754756 -0.49 0.625 -<br>.9195656 .5522717<br>| age_group | 2 | -.3685856 .3869402 -0.95 0.341 -<br>1.126974 .3898034<br>3 | -.4789782 .3833776 -1.25 0.212 -<br>1.230385 .2724282<br>4 | -.4778044 .4123968 -1.16 0.247 -<br>1.286087 .3304785<br>| race | 2 | .2532863 .3971284 0.64 0.524 -<br>.525071 1.031644<br>3 | -.1237677 .1893061 -0.65 0.513 -<br>.4948008 .2472655<br>4 | .1707448 .2612721 0.65 0.513 -<br>.3413391 .6828286<br>5 | 1.640979 .5146359 3.19 0.001<br>.6323112 2.649647<br>| ed | 2 | -.0302427 .2298874 -0.13 0.895 -<br>.4808137 .4203283<br>3 | .0526961 .2934373 0.18 0.857 -<br>.5224304 .6278226<br>4 | .3763782 .4484805 0.84 0.401 -<br>.5026273 1.255384<br>| pov | 2 | .1418385 .2662611 0.53 0.594 -<br>.3800237 .6637007<br>3 | .1552389 .2402878 0.65 0.518 -<br>.3157165 .6261943<br>4 | .6339783 .3061852 2.07 0.038<br>.0338664 1.23409<br>| 1.acgge3 | -.4091297 .2140094 -1.91 0.056 -<br>.8285805 .0103211<br>1.female | -.8177007 .1746598 -4.68 0.000 -<br>1.160028 -.4753738<br>1.hypertension | -.3437229 .1780099 -1.93 0.053 -<br>.6926159 .0051702<br>_cons | -1.212779 .4521288 -2.68 0.007 -<br>2.098935 -.3266227

© 2022 Lewis KH et al. *JAMA Network Open.*

		-------------------------+-- /lnalpha | -.4439185 .0326648 -13.59 0.000 -.5079403 -.3798968 -------------------------+-- alpha | .6415177 .020955 .6017337 .683932 ---
Radiology Costs	-- | Robust radio_costs | Coef. Std. Err. z P>|z| [95% Conf. Interval] -------------------------+-- radio_costs | yr | 1 | -.0983777 .0578583 -1.70 0.089 -.2117779 .0150225 2 | .1054298 .0741645 1.42 0.155 -.03993 .2507897 3 | .0726061 .0812 0.89 0.371 -.086543 .2317552 4 | .081345 .0952008 0.85 0.393 -.1052451 .2679352 | 1.sg | .067767 .0561294 1.21 0.227 -.0422447 .1777786 | sg#yr | 1 1 | -.0167109 .0714787 -0.23 0.815 -.1568067 .1233849 1 2 | -.0602862 .0881316 -0.68 0.494 -.2330211 .1124486 1 3 | -.0351873 .0976163 -0.36 0.718 -.2265117 .156137 1 4 | .0498874 .123319 0.40 0.686 -.1918134 .2915882 | age_group | 2 | .1172367 .0826326 1.42 0.156 -.0447202 .2791935 3 | -.0268034 .0794243 -0.34 0.736 -.1824722 .1288653 4 | .0853917 .0799103 1.07 0.285 -.0712297 .242013 | race | 2 | -.118746 .0965353 -1.23 0.219 -.3079518 .0704598 3 | -.0073602 .0436332 -0.17 0.866 -.0928798 .0781594 4 | -.0061193 .0644619 -0.09 0.924 -.1324623 .1202236 5 | -.2268979 .086144 -2.63 0.008 -.395737 -.0580588 | ed | 2 | .0138034 .053446 0.26 0.796 -.0909488 .1185556 3 | -.0362485 .0948081 -0.38 0.702 -.222069 .1495719 4 | -.2000782 .1555689 -1.29 0.198 -.5049876 .1048312 | pov | 2 | .0284413 .0505201 0.56 0.573 -.0705764 .1274589 3 | .0803888 .0530246 1.52 0.130 -.0235376 .1843152 4 | .0799327 .0696743 1.15 0.251 -.0566264 .2164919 |	--- | Robust radio_costs | Coef. Std. Err. z P>|z| [95% Conf. Interval] -------------------------+-- radio_costs | yr | 1 | -.1431256 .1140516 -1.25 0.210 -.3666625 .0804114 2 | -.0340264 .1302871 -0.26 0.794 -.2893846 .2213317 3 | -.1210278 .1189006 -1.02 0.309 -.3540687 .1120132 4 | -.1590116 .1201398 -1.32 0.186 -.3944812 .076458 | 1.sg | -.0542706 .1148993 -0.47 0.637 -.2794691 .1709279 | sg#yr | 1 1 | .1725097 .1572818 1.10 0.273 -.1357569 .4807763 1 2 | .1360493 .1730008 0.79 0.432 -.203026 .4751247 1 3 | -.0753803 .1537864 -0.49 0.624 -.376796 .2260354 1 4 | .1641649 .1555938 1.06 0.291 -.1407935 .4691232 | age_group | 2 | .2485602 .222039 1.12 0.263 -.1866283 .6837488 3 | .1692763 .2109729 0.80 0.422 -.244223 .5827756 4 | .2919947 .2158008 1.35 0.176 -.130967 .7149565 | race |

© 2022 Lewis KH et al. *JAMA Network Open.*

<table>
<tr>
<td></td>
<td>1.acgge3 | .342124 .0375924 9.10 0.000 .2684443 .4158038
1.female | .1476077 .0499961 2.95 0.003 .0496172 .2455982
1.hypertension | .115009 .0411999 2.79 0.005 .0342588 .1957593
_cons | 6.408663 .1026276 62.45 0.000 6.207517 6.60981
--------------------------+--
inflate |
yr |
1 | -.2488361 .0937757 -2.65 0.008 -.4326331 -.0650391
2 | .0874054 .0870921 1.00 0.316 -.0832919 .2581028
3 | .37234 .1193277 3.12 0.002 .138462 .6062179
4 | .1930033 .1295513 1.49 0.136 -.0609126 .4469192
|
1.sg | .0134136 .0820765 0.16 0.870 -.1474533 .1742806
|
sg#yr |
1 1 | -.0321957 .1072098 -0.30 0.764 -.242323 .1779317
1 2 | .1036199 .1011942 1.02 0.306 -.0947171 .3019569
1 3 | -.1242938 .1344298 -0.92 0.355 -.3877714 .1391838
1 4 | .0431533 .1524156 0.28 0.777 -.2555757 .3418824
|
age_group |
2 | .0075135 .1022497 0.07 0.941 -.1928923 .2079193
3 | -.3921033 .0994185 -3.94 0.000 -.58696 -.1972466
4 | -.5054711 .1062627 -4.76 0.000 -.7137422 -.2972
|
race |
2 | .1946108 .1762154 1.10 0.269 -.150765 .5399865
3 | .0145311 .0651468 0.22 0.823 -.1131543 .1422165
4 | .0132058 .0924777 0.14 0.886 -.1680471 .1944587
5 | .1985022 .193465 1.03 0.305 -.1806822 .5776866
|
ed |
2 | -.0286574 .0764602 -0.37 0.708 -.1785167 .1212019
3 | -.0594616 .1067218 -0.56 0.577 -.2686326 .1497093
4 | .0480921 .1758928 0.27 0.785 -.2966514 .3928357
|
pov |
2 | -.0069164 .0771146 -0.09 0.929 -.1580582 .1442254
3 | -.0290853 .0782493 -0.37 0.710 -.1824511 .1242804
4 | -.059427 .0987537 -0.60 0.547 -.2529808 .1341267
|
1.acgge3 | -.5941031 .0589617 -10.08 0.000 -.709666 -.4785402
1.female | -1.018432 .0647073 -15.74 0.000 -1.145256 -.8916077
1.hypertension | -.2151528 .0588317 -3.66 0.000 -.3304608 -.0998448
_cons | .8010272 .1344701 5.96 0.000 .5374706 1.064584
--------------------------+--
/lnalpha | .2637777 .0149002 17.70 0.000 .2345739 .2929816
--------------------------+--
alpha | 1.301839 .0193976 1.26437 1.340418
---</td>
<td>2 | .3898932 .3193424 1.22 0.222 -
.2360064 1.015793
3 | .0343078 .0877333 0.39 0.696 -
.1376463 .2062618
4 | -.0080053 .1236141 -0.06 0.948 -
.2502844 .2342738
5 | -.1662097 .2268061 -0.73 0.464 -
.6107414 .278322
|
ed |
2 | .0458487 .1025602 0.45 0.655 -
.1551657 .246863
3 | -.2387945 .1777526 -1.34 0.179 -
.5871831 .1095941
4 | -.5051115 .2210534 -2.29 0.022 -
.9383681 -.0718548
|
pov |
2 | .0243743 .123499 0.20 0.844 -
.2176794 .2664279
3 | .1946265 .1279677 1.52 0.128 -
.0561855 .4454386
4 | .3049094 .1562089 1.95 0.051 -
.0012544 .6110731
|
1.acgge3 | .5052607 .0858964 5.88 0.000
.3369068 .6736146
1.female | .2981366 .0930021 3.21 0.001
.1158559 .4804173
1.hypertension | .1327276 .0902426 1.47 0.141 -
.0441446 .3095997
_cons | 6.041564 .2599535 23.24 0.000
5.532064 6.551063
-------------------------+--
------
inflate |
yr |
1 | .0341008 .1530745 0.22 0.824 -
.2659197 .3341212
2 | .1122746 .1698401 0.66 0.509 -
.2206059 .4451552
3 | .1955811 .1622579 1.21 0.228 -
.1224385 .5136007
4 | .0638988 .1609414 0.40 0.691 -
.2515407 .3793382
|
1.sg | .1188051 .1670587 0.71 0.477 -
.2086239 .4462341
|
sg#yr |</td>
</tr>
</table>

© 2022 Lewis KH et al. *JAMA Network Open.*

		1 1 | -.4383713 .2145264 -2.04 0.041 -.8588353 -.0179072<br>1 2 | -.0381924 .2225686 -0.17 0.864 -.4744188 .398034<br>1 3 | -.1982063 .2201113 -0.90 0.368 -.6296166 .2332039<br>1 4 | -.2929149 .2230734 -1.31 0.189 -.7301307 .144301<br>| age_group | 2 | .0338561 .1876332 0.18 0.857 -.3338983 .4016104<br>3 | -.5854634 .1962059 -2.98 0.003 -.9700199 -.2009069<br>4 | -.729994 .2013716 -3.63 0.000 -1.124675 -.3353128<br>| race | 2 | .0276067 .2440437 0.11 0.910 -.4507102 .5059236<br>3 | .155929 .1192453 1.31 0.191 -.0777874 .3896455<br>4 | .0309156 .1646068 0.19 0.851 -.2917078 .353539<br>5 | .7972653 .4175088 1.91 0.056 -.0210369 1.615567<br>| ed | 2 | -.0061203 .1308988 -0.05 0.963 -.2626773 .2504367<br>3 | .2014592 .2210595 0.91 0.362 -.2318095 .6347278<br>4 | -.0066987 .407727 -0.02 0.987 -.8058289 .7924315<br>| pov | 2 | -.1208735 .1562761 -0.77 0.439 -.4271691 .1854221<br>3 | -.3216546 .1540776 -2.09 0.037 -.6236412 -.019668<br>4 | -.1566263 .2035231 -0.77 0.442 -.5555242 .2422716<br>| 1.acgge3 | -.4265006 .1204854 -3.54 0.000 -.6626477 -.1903536<br>1.female | -1.142403 .1097766 -10.41 0.000 -1.357562 -.9272452<br>1.hypertension | -.1208232 .1089897 -1.11 0.268 -.3344392 .0927928

© 2022 Lewis KH et al. *JAMA Network Open.*

<table>
<tr><td></td><td></td><td>_cons | .8198194 .2486932 3.30 0.001
.3323896 1.307249
-------------------------+--

/lnalpha | .2842487 .0278381 10.21 0.000
.2296871 .3388104
-------------------------+--
------
alpha | 1.328763 .0369902
1.258206 1.403277

-----</td></tr>
<tr><td>Prescription Drug Costs</td><td>--
| Robust
rx_costs | Coef. Std. Err. z P>|z| [95% Conf. Interval]
-------------------------+--
rx_costs |
yr |
1 | -.1705342 .0621566 -2.74 0.006 -.2923589 -.0487096
2 | -.3126355 .0623696 -5.01 0.000 -.4348777 -.1903932
3 | -.2055652 .0963439 -2.13 0.033 -.3943958 -.0167347
4 | -.3460955 .0893955 -3.87 0.000 -.5213075 -.1708834
|
1.sg | -.0256996 .054775 -0.47 0.639 -.1330566 .0816575
|
sg#yr |
1 1 | .0136664 .06894 0.20 0.843 -.1214535 .1487863
1 2 | .0929177 .0723936 1.28 0.199 -.0489712 .2348065
1 3 | .0727215 .1089194 0.67 0.504 -.1407566 .2861995
1 4 | .3622989 .1163677 3.11 0.002 .1342225 .5903754
|
age_group |
2 | .1730073 .1115103 1.55 0.121 -.0455488 .3915634
3 | .4080288 .1108722 3.68 0.000 .1907232 .6253344
4 | .643412 .1121419 5.74 0.000 .4236179 .8632061
|
race |
2 | -.540495 .138831 -3.89 0.000 -.8125988 -.2683912
3 | -.0668713 .0555468 -1.20 0.229 -.1757411 .0419984
4 | -.0861764 .1090604 -0.79 0.429 -.2999309 .127578
5 | .239019 .2177391 1.10 0.272 -.1877419 .6657798
|
ed |
2 | .001827 .0703741 0.03 0.979 -.1361038 .1397577
3 | .039085 .1313906 0.30 0.766 -.2184359 .2966059
4 | -.2839548 .1403927 -2.02 0.043 -.5591195 -.0087901
|
pov |
2 | -.0728461 .0792271 -0.92 0.358 -.2281283 .0824361
3 | -.0728087 .0786578 -0.93 0.355 -.2269752 .0813579</td><td>---
-----
| Robust
rx_costs | Coef. Std. Err. z P>|z| [95%
Conf. Interval]
-------------------------+--
------
rx_costs |
yr |
1 | -.4937288 .0873498 -5.65 0.000 -
.6649314 -.3225263
2 | -.4857936 .0923106 -5.26 0.000 -
.666719 -.3048681
3 | -.5169813 .0837744 -6.17 0.000 -
.681176 -.3527865
4 | -.5006418 .0843357 -5.94 0.000 -
.6659367 -.3353469
|
1.sg | -.0515433 .1058807 -0.49 0.626 -
.2590656 .1559791
|
sg#yr |
1 1 | .1455683 .1085746 1.34 0.180 -
.067234 .3583706
1 2 | .1513027 .1208193 1.25 0.210 -
.0854989 .3881043
1 3 | .2440906 .1148455 2.13 0.034
.0189976 .4691836
1 4 | .3239843 .129067 2.51 0.012
.0710177 .576951
|
age_group |
2 | .4035405 .2759293 1.46 0.144 -
.137271 .944352
3 | .5901417 .263868 2.24 0.025
.07297 1.107313
4 | .8408937 .2640814 3.18 0.001
.3233037 1.358484</td></tr>
</table>

<table>
<tr>
<td></td>
<td>4 | -.041682 .1000666 -0.42 0.677 -.2378089 .154445
|
1.acgge3 | .7713473 .0567981 13.58 0.000 .6600251 .8826695
1.female | .0330443 .0629273 0.53 0.600 -.0902909 .1563795
1.hypertension | .360518 .0608027 5.93 0.000 .2413469 .4796891
_cons | 6.943843 .1308055 53.09 0.000 6.687469 7.200217
-------------------------+--
inflate |
yr |
1 | -.505468 .2194586 -2.30 0.021 -.9355989 -.075337
2 | .4244897 .169056 2.51 0.012 .0931461 .7558333
3 | .5050154 .2113501 2.39 0.017 .0907767 .919254
4 | .956283 .2309828 4.14 0.000 .5035651 1.409001
|
1.sg | .0145546 .1806388 0.08 0.936 -.3394909 .3686
|
sg#yr |
1 1 | -.076541 .2480205 -0.31 0.758 -.5626522 .4095701
1 2 | -.0050212 .1921875 -0.03 0.979 -.3817018 .3716594
1 3 | .037652 .2360213 0.16 0.873 -.4249414 .5002453
1 4 | -.5833993 .2690681 -2.17 0.030 -1.110763 -.0560354
|
age_group |
2 | -.1558493 .2272273 -0.69 0.493 -.6012067 .2895081
3 | -.3797634 .2319951 -1.64 0.102 -.8344654 .0749386
4 | -.3117255 .266259 -1.17 0.242 -.8335835 .2101326
|
race |
2 | .0392341 .2924877 0.13 0.893 -.5340313 .6124995
3 | .2441256 .1513714 1.61 0.107 -.0525569 .5408081
4 | .40644 .196502 2.07 0.039 .0213032 .7915769
5 | 1.187303 .5266897 2.25 0.024 .1550105 2.219596
|
ed |
2 | .2681747 .1735859 1.54 0.122 -.0720474 .6083967
3 | .1282047 .2709352 0.47 0.636 -.4028186 .6592279
4 | .4062641 .3249384 1.25 0.211 -.2306034 1.043132
|
pov |
2 | -.3709562 .1770819 -2.09 0.036 -.7180304 -.023882
3 | -.1575044 .1847683 -0.85 0.394 -.5196437 .2046349
4 | -.3343985 .2239073 -1.49 0.135 -.7732487 .1044516
|
1.acgge3 | -.4383071 .1501053 -2.92 0.004 -.7325082 -.1441061
1.female | -.5590774 .1303571 -4.29 0.000 -.8145725 -.3035823
1.hypertension | -.5754367 .1414609 -4.07 0.000 -.852695 -.2981783
_cons | -1.530141 .328384 -4.66 0.000 -2.173762 -.8865198
-------------------------+--
/lnalpha | .7042579 .018675 37.71 0.000 .6676555 .7408602
-------------------------+--</td>
<td>|
race |
2 | -.1801266 .2980486 -0.60 0.546 -
.7642912 .4040381
3 | .0657322 .115816 0.57 0.570 -
.1612629 .2927274
4 | -.329598 .1994938 -1.65 0.098 -
.7205986 .0614027
5 | .3360065 .6071847 0.55 0.580 -
.8540537 1.526067
|
ed |
2 | -.0804313 .1373037 -0.59 0.558 -
.3495416 .1886791
3 | -.3633112 .2015585 -1.80 0.071 -
.7583587 .0317362
4 | -.4397689 .2440077 -1.80 0.072 -
.9180153 .0384774
|
pov |
2 | -.1860765 .1510164 -1.23 0.218 -
.4820633 .1099103
3 | -.1005999 .155999 -0.64 0.519 -
.4063523 .2051525
4 | .1118536 .1821879 0.61 0.539 -
.2452281 .4689354
|
1.acgge3 | .7263663 .1134985 6.40 0.000
.5039134 .9488193
1.female | .1278386 .1127148 1.13 0.257 -
.0930785 .3487556
1.hypertension | .3321164 .1110918 2.99 0.003
.1143804 .5498523
_cons | 6.942265 .2962897 23.43 0.000
6.361548 7.522982
-------------------------+--
------
inflate |
yr |
1 | -.2329745 .2469051 -0.94 0.345 -
.7168996 .2509506
2 | .4034409 .2210508 1.83 0.068 -
.0298107 .8366924
3 | .5409364 .2273057 2.38 0.017
.0954253 .9864474
4 | .5714718 .2266399 2.52 0.012
.1272657 1.015678
|
1.sg | .036134 .3753068 0.10 0.923 -
.6994539 .7717218</td>
</tr>
</table>

© 2022 Lewis KH et al. *JAMA Network Open.*

	alpha | 2.022345 .0377673 1.949661 2.097739<br>---	| sg#yr | 1 1 | -.3131016 .4182853 -0.75 0.454 -<br>1.132926 .5067226<br>1 2 | -.3035894 .3626838 -0.84 0.403 -<br>1.014437 .4072577<br>1 3 | -.2142781 .3550922 -0.60 0.546 -<br>.9102461 .4816899<br>1 4 | -.4235666 .3644806 -1.16 0.245 -<br>1.137935 .2908023<br>| age_group | 2 | -.9459465 .7899401 -1.20 0.231 -<br>2.494201 .6023077<br>3 | -.8005677 .7629902 -1.05 0.294 -<br>2.296001 .6948655<br>4 | -.083499 .7448239 -0.11 0.911 -<br>1.543327 1.376329<br>| race | 2 | -.5510348 .6360061 -0.87 0.386 -<br>1.797584 .6955142<br>3 | .5769611 .3074026 1.88 0.061 -<br>.025537 1.179459<br>4 | 1.125991 .4521116 2.49 0.013<br>.2398685 2.012113<br>5 | 1.329924 .503498 2.64 0.008<br>.3430865 2.316762<br>| ed | 2 | .6575163 .3405754 1.93 0.054 -<br>.0099993 1.325032<br>3 | .039057 .6020526 0.06 0.948 -<br>1.140944 1.219058<br>4 | 1.137008 .6888636 1.65 0.099 -<br>.2131396 2.487156<br>| pov | 2 | -1.584529 .5850361 -2.71 0.007 -<br>2.731179 -.4378797<br>3 | -.7966613 .3455789 -2.31 0.021 -<br>1.473983 -.1193391<br>4 | -.9854076 .4802259 -2.05 0.040 -<br>1.926633 -.044182<br>| 1.acgge3 | -.7094753 .4195096 -1.69 0.091 -<br>1.531699 .1127485<br>1.female | -.735657 .3091392 -2.38 0.017 -<br>1.341559 -.1297552

© 2022 Lewis KH et al. *JAMA Network Open.*

		1.hypertension | -.294141 .2574192 -1.14 0.253 -<br>.7986733 .2103913<br>_cons | -1.236753 .8514977 -1.45 0.146 -<br>2.905657 .4321523<br>-------------------------+--<br>------<br>/lnalpha | .6862978 .0436369 15.73 0.000<br>.6007712 .7718245<br>-------------------------+--<br>------<br>alpha | 1.986348 .086678 1.823525<br>2.16371<br>--<br>-----
All Other Ambulatory Costs	--<br>| Robust<br>tot_oth | Coef. Std. Err. z P>|z| [95% Conf. Interval]<br>-------------------------+--<br>tot_oth | yr | 1 | .1328835 .0840327 1.58 0.114 -.0318176 .2975846<br>2 | .3040801 .102824 2.96 0.003 .1025488 .5056114<br>3 | .2354862 .1288213 1.83 0.068 -.0169988 .4879712<br>4 | .4044952 .1602489 2.52 0.012 .0904131 .7185773<br>| 1.sg | .0045127 .0806922 0.06 0.955 -.1536411 .1626665<br>| sg#yr | 1 1 | -.0022616 .0978418 -0.02 0.982 -.1940279 .1895048<br>1 2 | -.0849259 .1181687 -0.72 0.472 -.3165322 .1466804<br>1 3 | -.0061565 .1471863 -0.04 0.967 -.2946364 .2823233<br>1 4 | -.1131812 .1825824 -0.62 0.535 -.4710361 .2446737<br>| age_group | 2 | .186165 .1309429 1.42 0.155 -.0704785 .4428084<br>3 | .163543 .1294347 1.26 0.206 -.0901444 .4172303<br>4 | .2624237 .131645 1.99 0.046 .0044041 .5204432<br>| race | 2 | -.2530714 .121571 -2.08 0.037 -.4913463 -.0147966<br>3 | -.0783434 .056648 -1.38 0.167 -.1893715 .0326846<br>4 | .0577266 .080301 0.72 0.472 -.0996605 .2151136<br>5 | -.2403823 .116447 -2.06 0.039 -.4686142 -.0121504<br>| ed | 2 | .0434898 .068762 0.63 0.527 -.0912812 .1782609<br>3 | -.0687685 .1018971 -0.67 0.500 -.2684832 .1309461<br>4 | -.1829989 .1717184 -1.07 0.287 -.5195607 .1535629<br>| pov |	--<br>-----<br>| Robust<br>tot_oth | Coef. Std. Err. z P>|z| [95%<br>Conf. Interval]<br>-------------------------+--<br>------<br>tot_oth | yr | 1 | .1155457 .1452339 0.80 0.426 -<br>.1691076 .4001989<br>2 | -.0008273 .1518377 -0.01 0.996 -<br>.2984237 .2967691<br>3 | .1115502 .1484964 0.75 0.453 -<br>.1794975 .4025978<br>4 | .2783599 .156687 1.78 0.076 -<br>.028741 .5854607<br>| 1.sg | .2494307 .1595343 1.56 0.118 -<br>.0632507 .5621122<br>| sg#yr | 1 1 | .0874313 .2003444 0.44 0.663 -<br>.3052364 .4800991<br>1 2 | .02706 .1993163 0.14 0.892 -<br>.3635929 .4177128<br>1 3 | -.132759 .2030408 -0.65 0.513 -<br>.5307116 .2651936<br>1 4 | -.1080049 .1992867 -0.54 0.588 -<br>.4985995 .2825898<br>| age_group | 2 | .4147111 .1920691 2.16 0.031<br>.0382626 .7911597<br>3 | .3955258 .1881079 2.10 0.035<br>.0268411 .7642104

© 2022 Lewis KH et al. *JAMA Network Open.*

<table>
<tr>
<td></td>
<td>2 | .0236295 .0724805 0.33 0.744 -.1184296 .1656887
3 | .0624261 .0722896 0.86 0.388 -.0792589 .2041111
4 | .1364181 .0985228 1.38 0.166 -.056683 .3295193
|
1.acgge3 | .490848 .0502402 9.77 0.000 .392379 .5893171
1.female | .0455648 .0670255 0.68 0.497 -.0858027 .1769324
1.hypertension | .1067603 .0545868 1.96 0.050 -.0002278 .2137484
_cons | 7.549077 .1571847 48.03 0.000 7.241001 7.857154
-------------------------+--
inflate |
yr |
1 | -.7946722 .1380337 -5.76 0.000 -1.065213 -.5241311
2 | -.0692403 .1217796 -0.57 0.570 -.307924 .1694434
3 | .2481916 .1430012 1.74 0.083 -.0320856 .5284689
4 | .17785 .1563114 1.14 0.255 -.1285148 .4842148
|
1.sg | -.2667815 .1059207 -2.52 0.012 -.4743823 -.0591807
|
sg#yr |
1 1 | .510844 .1544994 3.31 0.001 .2080308 .8136571
1 2 | .4834847 .1372256 3.52 0.000 .2145273 .752442
1 3 | .2578368 .1610375 1.60 0.109 -.0577909 .5734644
1 4 | .2138936 .1829196 1.17 0.242 -.1446223 .5724095
|
age_group |
2 | .0827092 .1255493 0.66 0.510 -.1633629 .3287813
3 | -.0779847 .1273912 -0.61 0.540 -.327667 .1716975
4 | -.3774365 .1518192 -2.49 0.013 -.6749968 -.0798763
|
race |
2 | .4558346 .1910848 2.39 0.017 .0813153 .830354
3 | .0721746 .0791757 0.91 0.362 -.083007 .2273562
4 | .2173199 .1366573 1.59 0.112 -.0505234 .4851633
5 | .0889787 .2367156 0.38 0.707 -.3749753 .5529328
|
ed |
2 | -.0502296 .0873826 -0.57 0.565 -.2214962 .1210371
3 | .0622954 .1502005 0.41 0.678 -.2320921 .3566829
4 | .0610839 .1836957 0.33 0.739 -.2989531 .4211209
|
pov |
2 | -.1155741 .1053722 -1.10 0.273 -.3220999 .0909517
3 | .0359534 .1097731 0.33 0.743 -.1791979 .2511048
4 | -.0860325 .1317081 -0.65 0.514 -.3441756 .1721105
|
1.acgge3 | -.6834582 .0803583 -8.51 0.000 -.8409575 -.5259588
1.female | -.2924878 .079299 -3.69 0.000 -.447911 -.1370646
1.hypertension | -.3126818 .075481 -4.14 0.000 -.4606218 -.1647419
_cons | -.5107319 .1745034 -2.93 0.003 -.8527523 -.1687116
-------------------------+--</td>
<td>4 | .5850426 .1842453 3.18 0.001
.2239285 .9461566
|
race |
2 | .6164908 .3893454 1.58 0.113 -
.1466122 1.379594
3 | -.0701187 .0932957 -0.75 0.452 -
.2529749 .1127374
4 | .3304392 .1741067 1.90 0.058 -
.0108036 .671682
5 | -.4569426 .2951031 -1.55 0.122 -
1.035334 .1214488
|
ed |
2 | .0644383 .1117553 0.58 0.564 -
.1545981 .2834747
3 | -.230622 .1683089 -1.37 0.171 -
.5605013 .0992574
4 | -.1405434 .2705404 -0.52 0.603 -
.6707928 .3897059
|
pov |
2 | .0606471 .1294198 0.47 0.639 -
.1930111 .3143052
3 | .3158251 .1410157 2.24 0.025
.0394394 .5922108
4 | .2365522 .1730163 1.37 0.172 -
.1025536 .575658
|
1.acgge3 | .5281509 .1005026 5.26 0.000
.3311695 .7251323
1.female | .2783929 .0974074 2.86 0.004
.087478 .4693078
1.hypertension | .1872584 .0979316 1.91 0.056 -
.004684 .3792008
_cons | 6.753022 .2394812 28.20 0.000
6.283647 7.222396
-------------------------+--
------
inflate |
yr |
1 | -.5833281 .211463 -2.76 0.006 -
.9977879 -.1688683
2 | .0773123 .1925423 0.40 0.688 -
.3000636 .4546882
3 | .1109324 .2135605 0.52 0.603 -
.3076385 .5295034
4 | .0954047 .184659 0.52 0.605 -
.2665203 .4573297
|</td>
</tr>
</table>

© 2022 Lewis KH et al. *JAMA Network Open.*

	/lnalpha | .9271828 .016106 57.57 0.000 .8956156 .95875<br>--------------------------+---<br>alpha | 2.527379 .040706 2.448843 2.608434<br>---	1.sg | .1035984 .2013131 0.51 0.607 -<br>.2909679 .4981648<br>| sg#yr | 1 1 | .2150613 .2853722 0.75 0.451 -<br>.3442578 .7743805<br>1 2 | -.1463518 .2584262 -0.57 0.571 -<br>.6528578 .3601542<br>1 3 | -.1295229 .282584 -0.46 0.647 -<br>.6833774 .4243316<br>1 4 | -.5096681 .2686145 -1.90 0.058 -<br>1.036143 .0168067<br>| age_group | 2 | -.1155044 .2356348 -0.49 0.624 -<br>.5773401 .3463313<br>3 | -.5258784 .2299602 -2.29 0.022 -<br>.9765921 -.0751648<br>4 | -.8844385 .2428625 -3.64 0.000 -<br>1.36044 -.4084368<br>| race | 2 | -.1402002 .3365302 -0.42 0.677 -<br>.7997872 .5193868<br>3 | -.0383517 .151313 -0.25 0.800 -<br>.3349197 .2582162<br>4 | -.0506869 .1906497 -0.27 0.790 -<br>.4243534 .3229797<br>5 | .8388189 .5803709 1.45 0.148 -<br>.2986873 1.976325<br>| ed | 2 | .2796787 .2057625 1.36 0.174 -<br>.1236084 .6829657<br>3 | .7161561 .263601 2.72 0.007<br>.1995076 1.232805<br>4 | .9910046 .3820389 2.59 0.009<br>.2422221 1.739787<br>| pov | 2 | .0834436 .1935719 0.43 0.666 -<br>.2959504 .4628375<br>3 | -.2429764 .2043386 -1.19 0.234 -<br>.6434727 .1575199<br>4 | -.2008229 .2613901 -0.77 0.442 -<br>.7131381 .3114923<br>| 1.acgge3 | -.7665635 .1631335 -4.70 0.000 -<br>1.086299 -.4468278

© 2022 Lewis KH et al. *JAMA Network Open.*

		1.female | -.2287813 .1436528 -1.59 0.111 -<br>.5103357 .0527731<br>1.hypertension | -.1552332 .1354429 -1.15 0.252 -<br>.4206964 .11023<br>_cons | -.520766 .309474 -1.68 0.092 -<br>1.127324 .0857919<br>-------------------------+--<br>------<br>/lnalpha | .9070918 .0327283 27.72 0.000<br>.8429456 .971238<br>-------------------------+--<br>------<br>alpha | 2.477108 .0810715 2.3232<br>2.641212<br>--<br>-----
Cardiometabolic prescription fills	--<br>| Robust<br>total_cardioenc_rx | Coef. Std. Err. z P>|z| [95% Conf. Interval]<br>-------------------------+--<br>total_cardioenc_rx | yr | 1 | -.5720415 .0419706 -13.63 0.000 -.6543024 -.4897806<br>2 | -.4362218 .0485649 -8.98 0.000 -.5314073 -.3410363<br>3 | -.387446 .0591197 -6.55 0.000 -.5033186 -.2715734<br>4 | -.337037 .0731765 -4.61 0.000 -.4804604 -.1936137<br>| 1.sg | -.0297384 .0421592 -0.71 0.481 -.1123688 .052892<br>| sg#yr | 1 1 | .0960093 .0494932 1.94 0.052 -.0009957 .1930143<br>1 2 | .122338 .057152 2.14 0.032 .0103221 .234354<br>1 3 | .1548836 .0709748 2.18 0.029 .0157756 .2939916<br>1 4 | .2286882 .0903698 2.53 0.011 .0515666 .4058098<br>| age_group | 2 | .3201345 .1429466 2.24 0.025 .0399642 .6003048<br>3 | .4724653 .1403093 3.37 0.001 .1974642 .7474664<br>4 | .7803743 .1396227 5.59 0.000 .5067189 1.05403<br>| race | 2 | .024822 .0880396 0.28 0.778 -.1477325 .1973765<br>3 | -.0288005 .0426199 -0.68 0.499 -.112334 .0547329<br>4 | -.0587859 .0810716 -0.73 0.468 -.2176833 .1001115<br>5 | -.1376413 .1305167 -1.05 0.292 -.3934493 .1181668<br>| ed | 2 | .0098426 .0519815 0.19 0.850 -.0920393 .1117246<br>3 | -.1169256 .1012429 -1.15 0.248 -.315358 .0815067<br>4 | -.2434724 .1333183 -1.83 0.068 -.5047714 .0178266	--<br>-----<br>| Robust<br>total_cardioenc_rx | Coef. Std. Err. z P>|z| [95%<br>Conf. Interval]<br>-------------------------+--<br>------<br>total_cardioenc_rx | yr | 1 | -.6443914 .0935982 -6.88 0.000 -<br>.8278405 -.4609422<br>2 | -.4000552 .0866706 -4.62 0.000 -<br>.5699265 -.2301839<br>3 | -.3114064 .0893663 -3.48 0.000 -<br>.4865611 -.1362518<br>4 | -.2999216 .0937911 -3.20 0.001 -<br>.4837489 -.1160944<br>| 1.sg | -.1230045 .0945459 -1.30 0.193 -<br>.308311 .062302<br>| sg#yr | 1 1 | .0999689 .1216048 0.82 0.411 -<br>.1383722 .33831<br>1 2 | .0274302 .1232089 0.22 0.824 -<br>.2140547 .2689151<br>1 3 | .0296841 .1182737 0.25 0.802 -<br>.2021281 .2614963<br>1 4 | .1128129 .1188975 0.95 0.343 -<br>.1202219 .3458477<br>| age_group | 2 | .5970131 .291255 2.05 0.040<br>.0261639 1.167862

© 2022 Lewis KH et al. *JAMA Network Open.*

	| pov | 2 | .0549902 .0566082 0.97 0.331 -.0559598 .1659402<br>3 | .0331416 .055898 0.59 0.553 -.0764166 .1426997<br>4 | .1532537 .0760078 2.02 0.044 .0042811 .3022262<br>| 1.acgge3 | .2600808 .0396892 6.55 0.000 .1822915 .3378701<br>1.female | -.3384918 .0425557 -7.95 0.000 -.4218994 -.2550843<br>1.hypertension | .5980588 .0495661 12.07 0.000 .500911 .6952066<br>_cons | 1.638308 .1535311 10.67 0.000 1.337392 1.939223<br>-------------------------+--<br>inflate | yr | 1 | .5117348 .095386 5.36 0.000 .3247817 .6986878<br>2 | 1.281706 .1017956 12.59 0.000 1.08219 1.481222<br>3 | 1.402362 .1251598 11.20 0.000 1.157053 1.647671<br>4 | 1.226908 .15835 7.75 0.000 .9165477 1.537269<br>| 1.sg | .0030626 .0933248 0.03 0.974 -.1798506 .1859758<br>| sg#yr | 1 1 | -.2053912 .1098418 -1.87 0.062 -.4206772 .0098948<br>1 2 | -.372234 .1180537 -3.15 0.002 -.6036151 -.140853<br>1 3 | -.4796711 .1443621 -3.32 0.001 -.7626156 -.1967267<br>1 4 | -.32 .1845401 -1.73 0.083 -.6816919 .041692<br>| age_group | 2 | -.589852 .1595063 -3.70 0.000 -.9024786 -.2772253<br>3 | -.9845534 .1537557 -6.40 0.000 -1.285909 -.6831977<br>4 | -1.575131 .1573887 -10.01 0.000 -1.883607 -1.266655<br>| race | 2 | -.6271661 .1886085 -3.33 0.001 -.9968319 -.2575002<br>3 | -.1333518 .079537 -1.68 0.094 -.2892414 .0225379<br>4 | .1425678 .1270091 1.12 0.262 -.1063654 .3915011<br>5 | -.2450821 .3042939 -0.81 0.421 -.8414871 .3513229<br>| ed | 2 | .0996593 .1003917 0.99 0.321 -.0971048 .2964234<br>3 | -.1213508 .1839534 -0.66 0.509 -.4818928 .2391911<br>4 | -.2469367 .2955117 -0.84 0.403 -.826129 .3322556<br>| pov | 2 | -.0703439 .1017404 -0.69 0.489 -.2697515 .1290636<br>3 | -.0029116 .0985452 -0.03 0.976 -.1960565 .1902334<br>4 | .0157262 .1311195 0.12 0.905 -.2412633 .2727156<br>| 1.acgge3 | -.4084441 .0747841 -5.46 0.000 -.5550182 -.26187<br>1.female | -.0258383 .0800088 -0.32 0.747 -.1826527 .1309762<br>1.hypertension | -1.785618 .0758354 -23.55 0.000 -1.934252 -1.636983	3 | .916039 .281783 3.25 0.001<br>.3637544 1.468324<br>4 | 1.116115 .2872483 3.89 0.000<br>.553119 1.679112<br>| race | 2 | -.2609244 .1694229 -1.54 0.124 -<br>.5929872 .0711385<br>3 | .0800232 .1147431 0.70 0.486 -<br>.1448692 .3049156<br>4 | -.1930889 .1767492 -1.09 0.275 -<br>.5395109 .1533332<br>5 | -.3820864 .1879374 -2.03 0.042 -<br>.7504369 -.0137359<br>| ed | 2 | -.108296 .1315978 -0.82 0.411 -<br>.3662229 .1496309<br>3 | -.1976596 .1974261 -1.00 0.317 -<br>.5846078 .1892885<br>4 | -.3880482 .3257707 -1.19 0.234 -<br>1.026547 .2504506<br>| pov | 2 | -.0821264 .1449207 -0.57 0.571 -<br>.3661657 .2019128<br>3 | -.0472302 .1508796 -0.31 0.754 -<br>.3429489 .2484885<br>4 | .1817492 .1644998 1.10 0.269 -<br>.1406644 .5041629<br>| 1.acgge3 | .2816635 .1011446 2.78 0.005<br>.0834237 .4799032<br>1.female | -.3979345 .0946471 -4.20 0.000 -<br>.5834395 -.2124296<br>1.hypertension | .4170242 .1423944 2.93 0.003<br>.1379364 .696112<br>_cons | 1.623636 .2890898 5.62 0.000<br>1.057031 2.190242<br>-------------------------+--<br>------<br>inflate | yr | 1 | .4542349 .143809 3.16 0.002<br>.1723745 .7360953<br>2 | 1.194873 .1606064 7.44 0.000<br>.8800903 1.509656<br>3 | 1.273658 .1642109 7.76 0.000<br>.9518109 1.595506

© 2022 Lewis KH et al. *JAMA Network Open.*

	_cons | 1.739811 .1917604 9.07 0.000 1.363967 2.115654<br>-------------------------+--<br>/lnalpha | -.091185 .0363267 -2.51 0.012 -.1623841 -.0199859<br>-------------------------+--<br>alpha | .9128488 .0331608 .8501146 .9802125<br>--	4 | 1.040244 .17517 5.94 0.000 .6969171 1.383571<br>| 1.sg | .0612325 .1889616 0.32 0.746 -.3091254 .4315904<br>| sg#yr | 1 1 | -.2230885 .2003395 -1.11 0.265 -.6157466 .1695696<br>1 2 | -.5055741 .2205843 -2.29 0.022 -.9379115 -.0732367<br>1 3 | -.5475963 .2210639 -2.48 0.013 -.9808735 -.114319<br>1 4 | -.4590875 .2306157 -1.99 0.047 -.9110859 -.0070891<br>| age_group | 2 | -.2454799 .4150694 -0.59 0.554 -1.059001 .5680411<br>3 | -.8084577 .4006357 -2.02 0.044 -1.593689 -.0232262<br>4 | -1.414036 .4183352 -3.38 0.001 -2.233958 -.594114<br>| race | 2 | -1.037323 .5142837 -2.02 0.044 -2.045301 -.029346<br>3 | -.1054047 .1755418 -0.60 0.548 -.4494603 .2386509<br>4 | .2907818 .258995 1.12 0.262 -.2168391 .7984027<br>5 | -.442834 .5874728 -0.75 0.451 -1.594259 .7085915<br>| ed | 2 | .0723159 .2242589 0.32 0.747 -.3672234 .5118553<br>3 | .1004796 .3282276 0.31 0.760 -.5428346 .7437938<br>4 | -.5136785 .4360541 -1.18 0.239 -1.368329 .3409719<br>| pov | 2 | -.2240782 .2338416 -0.96 0.338 -.6823992 .2342429<br>3 | -.4974739 .2584438 -1.92 0.054 -1.004014 .0090667<br>4 | -.3696045 .3084531 -1.20 0.231 -.9741615 .2349524<br>|

© 2022 Lewis KH et al. *JAMA Network Open.*

		1.acgge3 | -.4299097 .1753777 -2.45 0.014 -<br>.7736437 -.0861757<br>1.female | .0441929 .1793049 0.25 0.805 -<br>.3072382 .3956241<br>1.hypertension | -1.368524 .1711194 -8.00 0.000 -<br>1.703912 -1.033136<br>_cons | 1.513165 .4924823 3.07 0.002<br>.547917 2.478412<br>-------------------------+---<br>------<br>/lnalpha | -.0701975 .0827267 -0.85 0.396 -<br>.2323388 .0919438<br>-------------------------+---<br>------<br>alpha | .9322097 .0771186<br>.7926775 1.096303<br>--<br>-----
All other prescription fills	--<br>| Robust<br>total_othenc_rx | Coef. Std. Err. z P>|z| [95% Conf. Interval]<br>-------------------------+--<br>total_othenc_rx | yr | 1 | .0816724 .0314238 2.60 0.009 .0200829 .1432619<br>2 | .121962 .0345389 3.53 0.000 .054267 .189657<br>3 | .1401407 .0412091 3.40 0.001 .0593723 .2209091<br>4 | .1400031 .0547375 2.56 0.011 .0327196 .2472867<br>| 1.sg | -.0778051 .0391274 -1.99 0.047 -.1544934 -.0011168<br>| sg#yr | 1 1 | -.0401884 .0352773 -1.14 0.255 -.1093306 .0289538<br>1 2 | -.0049813 .0396672 -0.13 0.900 -.0827275 .072765<br>1 3 | .0506953 .049527 1.02 0.306 -.0463759 .1477664<br>1 4 | .0458935 .0673021 0.68 0.495 -.0860163 .1778032<br>| age_group | 2 | .1035895 .0810987 1.28 0.201 -.0553612 .2625401<br>3 | .1481989 .0732503 2.02 0.043 .0046309 .2917669<br>4 | .1583915 .0763847 2.07 0.038 .0086802 .3081028<br>| race | 2 | -.3539275 .1153996 -3.07 0.002 -.5801067 -.1277484<br>3 | -.0635612 .0414597 -1.53 0.125 -.1448207 .0176984<br>4 | -.1992081 .0849923 -2.34 0.019 -.3657899 -.0326264<br>5 | -.0267711 .1326683 -0.20 0.840 -.2867962 .2332541<br>| ed | 2 | -.0300408 .0538614 -0.56 0.577 -.1356073 .0755256	--<br>-----<br>| Robust<br>total_othenc_rx | Coef. Std. Err. z P>|z| [95%<br>Conf. Interval]<br>-------------------------+---<br>------<br>total_othenc_rx | yr | 1 | -.0629567 .0439729 -1.43 0.152 -<br>.149142 .0232285<br>2 | -.0248186 .0436222 -0.57 0.569 -<br>.1103166 .0606793<br>3 | .0756922 .0517872 1.46 0.144 -<br>.0258088 .1771933<br>4 | .1019901 .0480474 2.12 0.034<br>.0078189 .1961614<br>| 1.sg | -.1277545 .0765343 -1.67 0.095 -<br>.277759 .02225<br>| sg#yr | 1 1 | .1083861 .0589159 1.84 0.066 -<br>.007087 .2238591<br>1 2 | .1076931 .0626598 1.72 0.086 -<br>.0151179 .2305041<br>1 3 | .0482505 .0784301 0.62 0.538 -<br>.1054696 .2019707<br>1 4 | .0778062 .0663707 1.17 0.241 -<br>.052278 .2078904<br>| age_group |

© 2022 Lewis KH et al. *JAMA Network Open.*

```
           3  |  -.0405149   .0679456    -0.60   0.551    -.1736858     .092656
           4  |  -.0689004   .1747817    -0.39   0.693    -.4114662    .2736654
              |
         pov  |
           2  |   .0409036   .0520795     0.79   0.432    -.0611704    .1429777
           3  |   .0828496   .0489584     1.69   0.091    -.0131072    .1788064
           4  |   .1090633   .0625149     1.74   0.081    -.0134636    .2315902
              |
    1.acgge3  |   .5049659   .0397473    12.70   0.000     .4270626    .5828692
    1.female  |   .3433663   .0478769     7.17   0.000     .2495293    .4372032
1.hypertension |  .2150522   .0406627     5.29   0.000     .1353548    .2947497
       _cons  |   1.996967   .1001747    19.93   0.000     1.800629    2.193306
-------------------------+----------------------------------------------------------------
inflate                  |
          yr  |
           1  |  -.6459935     .37902    -1.70   0.088    -1.388859     .096872
           2  |   .5553892   .2031619     2.73   0.006     .1571991    .9535793
           3  |   .7046318    .286339     2.46   0.014     .1434177    1.265846
           4  |    1.15882   .2924896     3.96   0.000     .5855506    1.732089
              |
        1.sg  |  -.1303325   .2734287    -0.48   0.634    -.6662428    .4055779
              |
       sg#yr  |
         1 1  |  -.6305943   .5234051    -1.20   0.228     -1.65645    .3952609
         1 2  |   .1106097   .2435814     0.45   0.650     -.366801    .5880203
         1 3  |   .2865241   .3217175     0.89   0.373    -.3440307    .9170788
         1 4  |   -.335936    .340351    -0.99   0.324    -1.003012    .3311398
              |
   age_group  |
           2  |  -.0040118   .3193076    -0.01   0.990    -.6298431    .6218196
           3  |  -.2162888    .323651    -0.67   0.504    -.8506331    .4180555
           4  |  -.0963203     .37258    -0.26   0.796    -.8265637    .6339231
              |
        race  |
           2  |   .1365004   .3951741     0.35   0.730    -.6380266    .9110274
           3  |   .3295779   .1956461     1.68   0.092    -.0538814    .7130371
           4  |   .3733242   .2677824     1.39   0.163    -.1515197    .8981682
           5  |   1.190716   .6018388     1.98   0.048     .0111332    2.370298
              |
          ed  |
           2  |   .1778447   .2198933     0.81   0.419    -.2531382    .6088276
           3  |   .2303847   .3179152     0.72   0.469    -.3927177    .8534871
           4  |   .3597152   .3953009     0.91   0.363    -.4150604    1.134491
              |
         pov  |
           2  |  -.5717947   .2230399    -2.56   0.010    -1.008945   -.1346445
           3  |  -.2972768   .2267087    -1.31   0.190    -.7416178    .1470641
           4  |  -.4899107   .2859465    -1.71   0.087    -1.050355    .0705341
              |
    1.acgge3  |  -.4807394   .1861319    -2.58   0.010    -.8455512   -.1159276
```

```
           2  |   .2509628   .1969726     1.27   0.203    -
.1350964    .6370221
           3  |   .4218705   .1873022     2.25   0.024
.0547648    .7889761
           4  |   .6093695   .1910221     3.19   0.001
.2349729    .983766
              |
        race  |
           2  |   .0503097   .3991488     0.13   0.900    -
.7320075    .8326269
           3  |    .010314   .0840216     0.12   0.902    -
.1543653    .1749934
           4  |  -.3176051       .164    -1.94   0.053    -
.6390393    .003829
           5  |  -.0790887    .281361    -0.28   0.779    -
.630546    .4723687
              |
          ed  |
           2  |  -.0388467   .1106601    -0.35   0.726    -
.2557365    .1780432
           3  |  -.1325711   .1608311    -0.82   0.410    -
.4477942    .1826519
           4  |  -.2801603   .1630374    -1.72   0.086    -
.5997078    .0393872
              |
         pov  |
           2  |   .1692036   .1033566     1.64   0.102    -
.0333716    .3717788
           3  |   .0959691   .1010239     0.95   0.342    -
.1020341    .2939724
           4  |   .2291922   .1373393     1.67   0.095    -
.039988    .4983723
              |
    1.acgge3  |   .4529592   .0872169     5.19   0.000
.2820172    .6239012
    1.female  |   .4045715   .0953607     4.24   0.000
.2176679    .5914751
1.hypertension |   .072492   .0798054     0.91   0.364    -
.0839238    .2289078
       _cons  |   1.722628   .2089193     8.25   0.000
1.313154    2.132102
-------------------------+----------------------------------------------------------
------
inflate                  |
          yr  |
           1  |  -.1690039   .5805288    -0.29   0.771    -
1.306819    .9688116
           2  |   .4939856   .4454221     1.11   0.267    -
.3790257    1.366997
```

© 2022 Lewis KH et al. *JAMA Network Open.*

	1.female | -.7181844 .1548145 -4.64 0.000 -1.021615 -.4147536<br>1.hypertension | -.1248541 .183845 -0.68 0.497 -.4851836 .2354754<br>_cons | -1.807443 .4493793 -4.02 0.000 -2.688211 -.9266761<br>--------------------------+--<br>/lnalpha | -.0235702 .0281456 -0.84 0.402 -.0787346 .0315941<br>--------------------------+--<br>alpha | .9767054 .02749 .9242852 1.032099<br>---	3 | .5232502 .6128819 0.85 0.393 -<br>.6779763 1.724477<br>4 | .7984331 .5445071 1.47 0.143 -<br>.2687812 1.865647<br>| 1.sg | -.1209306 .7979716 -0.15 0.880 -<br>1.684926 1.443065<br>| sg#yr | 1 1 | -.2529622 .7496304 -0.34 0.736 -<br>1.722211 1.216286<br>1 2 | -.1614765 .6226769 -0.26 0.795 -<br>1.381901 1.058948<br>1 3 | .3261083 .8520071 0.38 0.702 -<br>1.343795 1.996012<br>1 4 | -.2134196 .727812 -0.29 0.769 -<br>1.639905 1.213066<br>| age_group | 2 | -1.18882 2.083057 -0.57 0.568 -<br>5.271537 2.893896<br>3 | -.9656559 1.837425 -0.53 0.599 -<br>4.566942 2.635631<br>4 | .1714152 1.682551 0.10 0.919 -<br>3.126324 3.469154<br>| race | 2 | .0141788 1.140098 0.01 0.990 -<br>2.220372 2.248729<br>3 | 1.004072 .8958505 1.12 0.262 -<br>.7517624 2.759907<br>4 | 1.305387 1.520773 0.86 0.391 -<br>1.675274 4.286048<br>5 | 2.132026 1.00422 2.12 0.034<br>.1637912 4.100261<br>| ed | 2 | .5865318 .9233091 0.64 0.525 -<br>1.223121 2.396184<br>3 | -.9330656 3.985114 -0.23 0.815 -<br>8.743746 6.877615<br>4 | 1.244529 1.578866 0.79 0.431 -<br>1.849992 4.33905<br>| pov | 2 | -2.960204 2.73054 -1.08 0.278 -<br>8.311964 2.391557<br>3 | -.9508897 .6088897 -1.56 0.118 -<br>2.144292 .2425121

© 2022 Lewis KH et al. *JAMA Network Open.*

		4 | -1.214739 1.574917 -0.77 0.441 -4.301519 1.87204<br>| 1.acgge3 | -1.298763 .8384965 -1.55 0.121 -2.942186 .3446602<br>1.female | -1.028256 .5041389 -2.04 0.041 -2.01635 -.0401624<br>1.hypertension | .098918 .6453535 0.15 0.878 -1.165952 1.363788<br>_cons | -1.605724 1.746445 -0.92 0.358 -5.028694 1.817245<br>-------------------------+---<br>/lnalpha | .0401148 .0958243 0.42 0.675 -.1476973 .2279269<br>-------------------------+---<br>alpha | 1.04093 .0997464 .8626922 1.255994<br>---
Specialist visits	--<br>| Semirobust<br>total_specenc | Coef. Std. Err. z P>|z| [95% Conf. Interval]<br>-------------------------+--<br>yr | 1 | .4747236 .0342981 13.84 0.000 .4075004 .5419467<br>2 | .3001423 .0406639 7.38 0.000 .2204424 .3798421<br>3 | .1849295 .0497954 3.71 0.000 .0873324 .2825266<br>4 | .0827283 .0509706 1.62 0.105 -.0171722 .1826288<br>| 1.sg | .0420778 .0423614 0.99 0.321 -.0409491 .1251047<br>| sg#yr | 1 1 | -.0751488 .0386073 -1.95 0.052 -.1508177 .0005201<br>1 2 | -.0806045 .0457376 -1.76 0.078 -.1702485 .0090394<br>1 3 | -.1195849 .056954 -2.10 0.036 -.2312127 -.0079572<br>1 4 | .0263774 .0614777 0.43 0.668 -.0941168 .1468715<br>| age_group | 2 | .0320761 .0649368 0.49 0.621 -.0951977 .1593498<br>3 | .0723751 .0633852 1.14 0.254 -.0518576 .1966079<br>4 | .281371 .0630714 4.46 0.000 .1577533 .4049887<br>| race | 2 | -.0536032 .0770626 -0.70 0.487 -.2046431 .0974367<br>3 | .0590915 .0359728 1.64 0.100 -.0114139 .1295969<br>4 | -.107693 .060655 -1.78 0.076 -.2265745 .0111886<br>5 | -.0802409 .1272344 -0.63 0.528 -.3296158 .1691339<br>|	---<br>| Semirobust<br>total_specenc | Coef. Std. Err. z P>|z| [95% Conf. Interval]<br>-------------------------+---<br>yr | 1 | .404372 .0677432 5.97 0.000 .2715978 .5371461<br>2 | .1752236 .0820223 2.14 0.033 .0144628 .3359844<br>3 | .054375 .0776542 0.70 0.484 -.0978244 .2065745<br>4 | .0236829 .0618048 0.38 0.702 -.0974523 .144818<br>| 1.sg | .0527602 .0809265 0.65 0.514 -.1058528 .2113732<br>| sg#yr | 1 1 | -.0112595 .084247 -0.13 0.894 -.1763805 .1538616<br>1 2 | -.050567 .0964601 -0.52 0.600 -.2396254 .1384914<br>1 3 | -.1060874 .0979784 -1.08 0.279 -.2981215 .0859467<br>1 4 | .057712 .084474 0.68 0.494 -.1078539 .223278

© 2022 Lewis KH et al. *JAMA Network Open.*

<table>
<tr>
<td></td>
<td>ed |
2 | -.0477002 .041193 -1.16 0.247 -.128437 .0330367
3 | -.1399146 .0654185 -2.14 0.032 -.2681325 -.0116967
4 | -.3482297 .1106357 -3.15 0.002 -.5650717 -.1313878
|
pov |
2 | -.0393148 .0455691 -0.86 0.388 -.1286286 .049999
3 | -.0876918 .0459792 -1.91 0.056 -.1778094 .0024259
4 | -.0381741 .057561 -0.66 0.507 -.1509915 .0746433
|
1.female | .3024765 .0374656 8.07 0.000 .2290452 .3759078
1.hypertension | .183493 .0332848 5.51 0.000 .118256 .2487299
_cons | .2788069 .0804927 3.46 0.001 .1210441 .4365697
--</td>
<td>|
age_group |
2 | .265891 .1371718 1.94 0.053 -
.0029608 .5347427
3 | .3272 .134248 2.44 0.015
.0640787 .5903212
4 | .5049018 .1301832 3.88 0.000
.2497473 .7600563
|
race |
2 | .1159701 .2868761 0.40 0.686 -
.4462968 .678237
3 | -.0245539 .0710196 -0.35 0.730 -
.1637497 .114642
4 | -.124337 .1163771 -1.07 0.285 -
.352432 .103758
5 | -.3069899 .3798076 -0.81 0.419 -
1.051399 .4374193
|
ed |
2 | .0218082 .0906747 0.24 0.810 -
.1559109 .1995274
3 | -.4321947 .163942 -2.64 0.008 -
.7535152 -.1108743
4 | -.5233302 .2288133 -2.29 0.022 -
.971796 -.0748644
|
pov |
2 | .0307141 .092245 0.33 0.739 -
.1500828 .2115111
3 | .0788816 .093785 0.84 0.400 -
.1049336 .2626969
4 | .2012108 .1342111 1.50 0.134 -
.0618382 .4642597
|
1.female | .42261 .0794896 5.32 0.000
.2668133 .5784067
1.hypertension | .1675953 .0675449 2.48 0.013
.0352098 .2999808
_cons | -.1317668 .1900968 -0.69 0.488 -
.5043497 .2408161

-----</td>
</tr>
<tr>
<td>PCP visits</td>
<td>--
| Semirobust
total_pcpenc | Coef. Std. Err. z P>|z| [95% Conf. Interval]
-------------+--
yr |
1 | .0236439 .0250813 0.94 0.346 -.0255145 .0728023
2 | -.0559703 .0288469 -1.94 0.052 -.1125091 .0005685</td>
<td>--

| Semirobust
total_pcpenc | Coef. Std. Err. z P>|z| [95%
Conf. Interval]
-------------+--
------</td>
</tr>
</table>

© 2022 Lewis KH et al. *JAMA Network Open.*

	3 | -.1078016 .0372197 -2.90 0.004 -.180751 -.0348522<br>4 | -.1599189 .0421797 -3.79 0.000 -.2425895 -.0772482<br>| 1.sg | -.0210292 .0260818 -0.81 0.420 -.0721485 .0300901<br>| sg#yr | 1 1 | -.0439559 .0287814 -1.53 0.127 -.1003664 .0124547<br>1 2 | -.0482026 .0334416 -1.44 0.149 -.1137469 .0173416<br>1 3 | -.039133 .0435136 -0.90 0.368 -.1244181 .046152<br>1 4 | .0141943 .0511154 0.28 0.781 -.0859901 .1143788<br>| age_group | 2 | .0326058 .057069 0.57 0.568 -.0792474 .144459<br>3 | .1419268 .0550556 2.58 0.010 .0340198 .2498337<br>4 | .2218921 .0567502 3.91 0.000 .1106637 .3331205<br>| race | 2 | -.0554974 .0498604 -1.11 0.266 -.153222 .0422273<br>3 | .0245764 .0245758 1.00 0.317 -.0235912 .0727441<br>4 | -.0210216 .0466633 -0.45 0.652 -.11248 .0704367<br>5 | .1697627 .1086749 1.56 0.118 -.0432362 .3827616<br>| ed | 2 | -.0009229 .0278153 -0.03 0.974 -.0554399 .0535941<br>3 | .0965087 .0486818 1.98 0.047 .001094 .1919233<br>4 | -.0153658 .0709186 -0.22 0.828 -.1543637 .1236321<br>| pov | 2 | .0160223 .0339499 0.47 0.637 -.0505184 .0825629<br>3 | -.0213043 .0341208 -0.62 0.532 -.0881798 .0455712<br>4 | .0035015 .0400793 0.09 0.930 -.0750525 .0820555<br>| 1.female | .1193839 .0265603 4.49 0.000 .0673266 .1714412<br>1.hypertension | .2037003 .0244305 8.34 0.000 .1558175 .2515832<br>_cons | .2750765 .0682769 4.03 0.000 .1412562 .4088969<br>--	yr | 1 | .0071017 .0468613 0.15 0.880 -.0847447 .0989482<br>2 | -.088046 .0489705 -1.80 0.072 -.1840265 .0079345<br>3 | -.1733697 .0552584 -3.14 0.002 -.2816742 -.0650653<br>4 | -.1913548 .0488025 -3.92 0.000 -.287006 -.0957037<br>| 1.sg | -.0782914 .0514809 -1.52 0.128 -.1791922 .0226093<br>| sg#yr | 1 1 | .0843866 .0614736 1.37 0.170 -.0360994 .2048726<br>1 2 | .0849148 .0648557 1.31 0.190 -.0422 .2120296<br>1 3 | .1065939 .0731983 1.46 0.145 -.0368721 .25006<br>1 4 | .1439202 .0675368 2.13 0.033 .0115504 .2762899<br>| age_group | 2 | -.1273936 .10738 -1.19 0.235 -.3378544 .0830673<br>3 | .0080311 .103251 0.08 0.938 -.1943371 .2103993<br>4 | .069285 .1051952 0.66 0.510 -.1368937 .2754638<br>| race | 2 | -.0477748 .1181047 -0.40 0.686 -.2792558 .1837062<br>3 | .0705181 .0416673 1.69 0.091 -.0111484 .1521846<br>4 | -.0237542 .0782404 -0.30 0.761 -.1771026 .1295942<br>5 | -.4322815 .3115299 -1.39 0.165 -1.042869 .178306<br>| ed | 2 | .0254795 .0571006 0.45 0.655 -.0864356 .1373947<br>3 | .1988378 .0927584 2.14 0.032 .0170347 .3806408<br>4 | -.1434955 .1316783 -1.09 0.276 -.4015801 .1145891<br>| pov |

© 2022 Lewis KH et al. *JAMA Network Open.*

<table>
<tr>
<td></td>
<td></td>
<td>2 | .0882115 .0543297 1.62 0.104 -
.0182726 .1946957
3 | .0024154 .0542078 0.04 0.964 -
.1038299 .1086607
4 | .0597654 .0718439 0.83 0.405 -
.081046 .2005768
|
1.female | .1389992 .0437157 3.18 0.001
.0533179 .2246805
1.hypertension | .145581 .0406572 3.58 0.000
.0658944 .2252676
_cons | .3808013 .1155205 3.30 0.001
.1543852 .6072174

-----</td>
</tr>
<tr>
<td>Nutrition labs</td>
<td>--
| Robust
total_nutrienc_lab | Coef. Std. Err. z P>|z| [95% Conf. Interval]
-------------------------+--
total_nutrienc_lab |
yr |
1 | 1.558986 .083748 18.62 0.000 1.394843 1.723129
2 | 1.308094 .0901874 14.50 0.000 1.13133 1.484858
3 | 1.110304 .0948291 11.71 0.000 .9244419 1.296165
4 | 1.024766 .1099883 9.32 0.000 .8091928 1.240339
|
1.sg | -.0502992 .0908934 -0.55 0.580 -.2284469 .1278485
|
sg#yr |
1 1 | -.0669328 .0962888 -0.70 0.487 -.2556554 .1217897
1 2 | -.0678712 .1047814 -0.65 0.517 -.2732391 .1374966
1 3 | -.0481238 .1150993 -0.42 0.676 -.2737143 .1774667
1 4 | -.1781222 .1361091 -1.31 0.191 -.4448912 .0886468
|
age_group |
2 | .0794643 .0765446 1.04 0.299 -.0705604 .2294889
3 | .0519786 .0727534 0.71 0.475 -.0906155 .1945726
4 | .132348 .0742127 1.78 0.075 -.0131062 .2778021
|
race |
2 | .050271 .0638478 0.79 0.431 -.0748683 .1754103
3 | .0801825 .0365544 2.19 0.028 .0085373 .1518278
4 | .0856064 .0541536 1.58 0.114 -.0205327 .1917456
5 | .093392 .1349514 0.69 0.489 -.1711078 .3578919
|
ed |
2 | .0025906 .0459127 0.06 0.955 -.0873967 .0925779
3 | .0077152 .0725253 0.11 0.915 -.1344318 .1498622
4 | .0853736 .1753606 0.49 0.626 -.2583268 .4290741
|</td>
<td>--

| Robust
total_nutrienc_lab | Coef. Std. Err. z P>|z| [95%
Conf. Interval]
-------------------------+--
------
total_nutrienc_lab |
yr |
1 | 1.659241 .1755207 9.45 0.000
1.315226 2.003255
2 | 1.35931 .18876 7.20 0.000
.9893475 1.729273
3 | 1.071193 .1938353 5.53 0.000
.6912827 1.451103
4 | .825339 .1826911 4.52 0.000
.4672709 1.183407
|
1.sg | .2124667 .2241863 0.95 0.343 -
.2269303 .6518638
|
sg#yr |
1 1 | -.2670844 .222487 -1.20 0.230 -
.7031509 .1689821
1 2 | -.3364203 .2380799 -1.41 0.158 -
.8030484 .1302078
1 3 | -.3261039 .2512318 -1.30 0.194 -
.8185091 .1663013
1 4 | -.2702081 .2424069 -1.11 0.265 -
.7453169 .2049007
|
age_group |
2 | .3682101 .1493957 2.46 0.014
.0753999 .6610203</td>
</tr>
</table>

<table>
<tr>
<td></td>
<td>pov |
2 | -.1037688 .0434813 -2.39 0.017 -.1889906 -.018547
3 | -.1457813 .0455297 -3.20 0.001 -.2350179 -.0565447
4 | -.2037179 .0600133 -3.39 0.001 -.3213418 -.0860941
|
1.acgge3 | .0759314 .0317645 2.39 0.017 .0136741 .1381887
1.female | .0586966 .0398909 1.47 0.141 -.0194881 .1368813
1.hypertension | -.0163561 .0339385 -0.48 0.630 -.0828744 .0501622
_cons | .4134045 .1158367 3.57 0.000 .1863688 .6404403
-------------------------+--
inflate |
yr |
1 | -1.408799 .1296055 -10.87 0.000 -1.662821 -1.154777
2 | -.552669 .128959 -4.29 0.000 -.8054239 -.2999141
3 | -.1362191 .1297559 -1.05 0.294 -.3905359 .1180978
4 | -.1972887 .1537895 -1.28 0.200 -.4987107 .1041332
|
1.sg | -.1690702 .117337 -1.44 0.150 -.3990465 .0609061
|
sg#yr |
1 1 | .3971119 .146259 2.72 0.007 .1104495 .6837743
1 2 | .4215019 .1461365 2.88 0.004 .1350797 .7079241
1 3 | .3243195 .1520102 2.13 0.033 .026385 .6222539
1 4 | .3370033 .1826201 1.85 0.065 -.0209255 .6949321
|
age_group |
2 | -.1259268 .1238594 -1.02 0.309 -.3686868 .1168332
3 | -.3661917 .1231283 -2.97 0.003 -.6075188 -.1248646
4 | -.5981442 .1278377 -4.68 0.000 -.8487015 -.3475869
|
race |
2 | -.3310927 .1524536 -2.17 0.030 -.6298963 -.0322891
3 | -.1954777 .0700473 -2.79 0.005 -.3327678 -.0581876
4 | -.2971164 .1144427 -2.60 0.009 -.5214199 -.0728129
5 | -.6789325 .2910994 -2.33 0.020 -1.249477 -.1083881
|
ed |
2 | .0664279 .0826169 0.80 0.421 -.0954983 .2283541
3 | .0542389 .1348819 0.40 0.688 -.2101248 .3186025
4 | -.0955406 .2306601 -0.41 0.679 -.5476261 .3565449
|
pov |
2 | -.0297892 .082918 -0.36 0.719 -.1923054 .1327271
3 | .1004982 .0845356 1.19 0.235 -.0651885 .2661849
4 | .1621719 .1117329 1.45 0.147 -.0568206 .3811643
|
1.acgge3 | -.1236903 .0629131 -1.97 0.049 -.2469978 -.0003829
1.female | -.355032 .0704307 -5.04 0.000 -.4930737 -.2169903
1.hypertension | -.3003677 .0653406 -4.60 0.000 -.4284329 -.1723025
_cons | 1.267307 .1774344 7.14 0.000 .9195419 1.615072</td>
<td>3 | .4607888 .1470458 3.13 0.002
.1725842 .7489933
4 | .371292 .1595863 2.33 0.020
.0585087 .6840754
|
race |
2 | .0346369 .1468461 0.24 0.814 -
.2531763 .32245
3 | .0197914 .073384 0.27 0.787 -
.1240387 .1636214
4 | -.0047092 .0982271 -0.05 0.962 -
.1972309 .1878124
5 | -.6454797 .1813517 -3.56 0.000 -
1.000922 -.2900369
|
ed |
2 | .0776897 .0780958 0.99 0.320 -
.0753753 .2307547
3 | -.0030923 .101879 -0.03 0.976 -
.2027715 .1965869
4 | .1914559 .1514169 1.26 0.206 -
.1053159 .4882276
|
pov |
2 | .1367627 .0818237 1.67 0.095 -
.0236088 .2971343
3 | .1254812 .0802326 1.56 0.118 -
.0317717 .2827341
4 | -.0763559 .1128977 -0.68 0.499 -
.2976313 .1449196
|
1.acgge3 | .0479154 .063974 0.75 0.454 -
.0774713 .173302
1.female | .1648368 .0836351 1.97 0.049
.0009151 .3287585
1.hypertension | -.0765798 .067812 -1.13 0.259 -
.2094889 .0563293
_cons | -.2547659 .2298271 -1.11 0.268 -
.7052188 .195687
-------------------------+--
------
inflate |
yr |
1 | -1.339702 .2498884 -5.36 0.000 -
1.829475 -.84993
2 | -.5994264 .2480218 -2.42 0.016 -
1.08554 -.1133125
3 | -.3120255 .2428538 -1.28 0.199 -
.7880101 .1639592</td>
</tr>
</table>

© 2022 Lewis KH et al. *JAMA Network Open.*

	--------------------------+--<br>/lnalpha | -.4475073 .040339 -11.09 0.000 -.5265702 -.3684444<br>--------------------------+--<br>alpha | .6392196 .0257855 .5906272 .6918097<br>---	4 | -.6580842 .2509002 -2.62 0.009 -<br>1.14984 -.166329<br>| 1.sg | .0663003 .2678744 0.25 0.805 -<br>.4587239 .5913244<br>| sg#yr | 1 1 | -.0450823 .3081804 -0.15 0.884 -<br>.6491048 .5589403<br>1 2 | -.117627 .3124938 -0.38 0.707 -<br>.7301035 .4948495<br>1 3 | -.1912329 .3060321 -0.62 0.532 -<br>.7910448 .408579<br>1 4 | .110722 .3165309 0.35 0.726 -<br>.5096671 .7311111<br>| age_group | 2 | .2374371 .2958753 0.80 0.422 -<br>.3424678 .8173421<br>3 | -.0088575 .2837447 -0.03 0.975 -<br>.5649869 .5472719<br>4 | -.1341311 .3013019 -0.45 0.656 -<br>.724672 .4564098<br>| race | 2 | -.2094821 .2552834 -0.82 0.412 -<br>.7098284 .2908642<br>3 | -.2334425 .1506818 -1.55 0.121 -<br>.5287735 .0618885<br>4 | -.2594366 .2032634 -1.28 0.202 -<br>.6578254 .1389523<br>5 | .4630683 .6262756 0.74 0.460 -<br>.7644093 1.690546<br>| ed | 2 | .3505841 .180673 1.94 0.052 -<br>.0035284 .7046966<br>3 | .1573981 .278725 0.56 0.572 -<br>.3888928 .703689<br>4 | .2536932 .4709136 0.54 0.590 -<br>.6692805 1.176667<br>| pov | 2 | .0654676 .1775278 0.37 0.712 -<br>.2824804 .4134157<br>3 | -.055088 .1813672 -0.30 0.761 -<br>.4105612 .3003852<br>4 | -.0893918 .2379396 -0.38 0.707 -<br>.5557449 .3769613<br>|

© 2022 Lewis KH et al. *JAMA Network Open.*

<table>
<tr>
<td></td>
<td></td>
<td>1.acgge3 | -.0906855 .1424166 -0.64 0.524 -
.3698168 .1884458
1.female | -.3492924 .1417856 -2.46 0.014 -
.6271871 -.0713978
1.hypertension | -.2966151 .1280606 -2.32 0.021 -
.5476093 -.0456209
_cons | .9722473 .390313 2.49 0.013
.2072479 1.737247
-------------------------+--

/lnalpha | -.6238793 .07877 -7.92 0.000 -
.7782657 -.469493
-------------------------+--
------
alpha | .5358616 .0422098
.4592017 .6253193

-----</td>
</tr>
<tr>
<td>All other labs</td>
<td>--
| Robust
total_otherenc_lab | Coef. Std. Err. z P>|z| [95% Conf. Interval]
-------------------------+--
total_otherenc_lab |
yr |
1 | .4095288 .0402961 10.16 0.000 .3305498 .4885077
2 | .2969019 .0487969 6.08 0.000 .2012618 .392542
3 | .2377304 .0549999 4.32 0.000 .1299327 .3455281
4 | .2785865 .0846523 3.29 0.001 .1126711 .4445019
|
1.sg | .0756122 .0427929 1.77 0.077 -.0082603 .1594847
|
sg#yr |
1 1 | -.116014 .0475617 -2.44 0.015 -.2092333 -.0227947
1 2 | -.1239741 .057903 -2.14 0.032 -.2374618 -.0104863
1 3 | -.0379108 .067455 -0.56 0.574 -.1701202 .0942986
1 4 | -.014871 .1252323 -0.12 0.905 -.2603219 .2305798
|
age_group |
2 | -.092179 .0793509 -1.16 0.245 -.2477038 .0633458
3 | -.1084636 .0783361 -1.38 0.166 -.2619995 .0450723
4 | -.0298906 .0789958 -0.38 0.705 -.1847195 .1249383
|
race |
2 | .1034896 .071985 1.44 0.151 -.0375984 .2445776
3 | .0819628 .0327499 2.50 0.012 .0177742 .1461514
4 | .196272 .0521308 3.76 0.000 .0940976 .2984465
5 | .3539617 .2267067 1.56 0.118 -.0903752 .7982987
|
ed |
2 | -.01683 .0427045 -0.39 0.694 -.1005293 .0668692</td>
<td>---

| Robust
total_otherenc_lab | Coef. Std. Err. z P>|z| [95%
Conf. Interval]
-------------------------+--
------
total_otherenc_lab |
yr |
1 | .4148735 .068661 6.04 0.000
.2803004 .5494466
2 | .1937466 .0985285 1.97 0.049
.0006343 .386859
3 | .2463891 .1040872 2.37 0.018
.042382 .4503962
4 | .1916761 .0797418 2.40 0.016
.035385 .3479671
|
1.sg | .1993808 .0899473 2.22 0.027
.0230873 .3756744
|
sg#yr |
1 1 | -.1346567 .0965533 -1.39 0.163 -
.3238978 .0545843
1 2 | -.1361159 .1290177 -1.06 0.291 -
.388986 .1167541
1 3 | -.179441 .1321911 -1.36 0.175 -
.4385308 .0796487
1 4 | .072428 .1664146 0.44 0.663 -
.2537386 .3985946
|
age_group |</td>
</tr>
</table>

© 2022 Lewis KH et al. *JAMA Network Open.*

<table>
<tr>
<td></td>
<td>3 | .0852573 .0769677 1.11 0.268 -.0655966 .2361111
4 | .0919016 .1093253 0.84 0.401 -.122372 .3061751
|
pov |
2 | -.0166834 .0407475 -0.41 0.682 -.0965471 .0631803
3 | -.006826 .0442941 -0.15 0.878 -.0936409 .0799888
4 | -.0026761 .0559641 -0.05 0.962 -.1123637 .1070115
|
1.acgge3 | .3152259 .030675 10.28 0.000 .255104 .3753477
1.female | .1023298 .0378866 2.70 0.007 .0280735 .1765861
1.hypertension | .0970635 .0321485 3.02 0.003 .0340535 .1600735
_cons | 2.210431 .1025049 21.56 0.000 2.009525 2.411337
-------------------------+--
inflate |
yr |
1 | -.8127573 .1724625 -4.71 0.000 -1.150778 -.474737
2 | -.0587618 .1461335 -0.40 0.688 -.3451782 .2276546
3 | .5186038 .1545397 3.36 0.001 .2157116 .821496
4 | .5921699 .1574928 3.76 0.000 .2834897 .9008501
|
1.sg | -.1045245 .1202254 -0.87 0.385 -.3401619 .131113
|
sg#yr |
1 1 | .3311346 .1891891 1.75 0.080 -.0396692 .7019383
1 2 | .3606118 .1623481 2.22 0.026 .0424154 .6788083
1 3 | .1146825 .1739913 0.66 0.510 -.2263342 .4556991
1 4 | .0705629 .1851037 0.38 0.703 -.2922337 .4333595
|
age_group |
2 | .1770541 .1416391 1.25 0.211 -.1005533 .4546616
3 | .0236412 .1369956 0.17 0.863 -.2448652 .2921476
4 | -.0954665 .1510257 -0.63 0.527 -.3914715 .2005385
|
race |
2 | -.1160258 .1921369 -0.60 0.546 -.4926073 .2605557
3 | -.2830627 .0902978 -3.13 0.002 -.4600432 -.1060823
4 | -.1732908 .1129073 -1.53 0.125 -.394585 .0480034
5 | -.0728366 .2578563 -0.28 0.778 -.5782256 .4325524
|
ed |
2 | -.019511 .0960871 -0.20 0.839 -.2078383 .1688162
3 | .1086972 .1300727 0.84 0.403 -.1462405 .363635
4 | -.1023472 .2404819 -0.43 0.670 -.573683 .3689886
|
pov |
2 | .1130161 .1022621 1.11 0.269 -.0874139 .3134461
3 | .2762149 .1061044 2.60 0.009 .068254 .4841758
4 | .2867408 .13384 2.14 0.032 .0244192 .5490625
|
1.acgge3 | -.2947716 .0807919 -3.65 0.000 -.4531209 -.1364223</td>
<td>2 | .1544303 .1494999 1.03 0.302 -
.1385842 .4474447
3 | .2143755 .1393518 1.54 0.124 -
.0587491 .4875001
4 | .2904294 .1394425 2.08 0.037
.017127 .5637317
|
race |
2 | .508912 .2763723 1.84 0.066 -
.0327677 1.050592
3 | .1883734 .0814178 2.31 0.021
.0287975 .3479492
4 | .2364006 .090529 2.61 0.009
.0589671 .4138341
5 | -.4806937 .1914276 -2.51 0.012 -
.8558848 -.1055026
|
ed |
2 | -.0317971 .1014498 -0.31 0.754 -
.2306352 .1670409
3 | -.1400096 .1338865 -1.05 0.296 -
.4024223 .1224032
4 | -.0397755 .1780381 -0.22 0.823 -
.3887239 .3091728
|
pov |
2 | -.0271729 .0847938 -0.32 0.749 -
.1933657 .1390199
3 | .1093834 .0971961 1.13 0.260 -
.0811175 .2998843
4 | .238814 .1132159 2.11 0.035
.016915 .460713
|
1.acgge3 | .35416 .0687331 5.15 0.000
.2194456 .4888744
1.female | .1138966 .078837 1.44 0.149 -
.0406212 .2684144
1.hypertension | .0238164 .0679617 0.35 0.726 -
.1093861 .1570189
_cons | 1.728831 .1795555 9.63 0.000
1.376909 2.080753
-------------------------+--
------
inflate |
yr |
1 | -.8552347 .3071156 -2.78 0.005 -
1.45717 -.2532992
2 | .1052147 .2241729 0.47 0.639 -
.3341562 .5445855</td>
</tr>
</table>

© 2022 Lewis KH et al. *JAMA Network Open.*

<table>
<tr>
<td></td>
<td>1.female | -.7334659 .0780074 -9.40 0.000 -.8863576 -.5805742
1.hypertension | -.3629896 .076991 -4.71 0.000 -.5138891 -.21209
_cons | -.6969637 .1940775 -3.59 0.000 -1.077349 -.3165788
-------------------------+--
/lnalpha | -.160591 .0287876 -5.58 0.000 -.2170137 -.1041682
-------------------------+--
alpha | .8516404 .0245167 .804919 .9010737
--</td>
<td>3 | .680505 .2774006 2.45 0.014
.1368099 1.2242
4 | .506038 .243409 2.08 0.038
.0289651 .9831109
|
1.sg | .2269095 .2725865 0.83 0.405 -
.3073502 .7611692
|
sg#yr |
1 1 | .0939872 .3927211 0.24 0.811 -
.6757321 .8637065
1 2 | -.5154767 .3292015 -1.57 0.117 -
1.1607 .1297463
1 3 | -.5366236 .3471808 -1.55 0.122 -
1.217085 .1438383
1 4 | -.4792877 .322889 -1.48 0.138 -
1.112139 .1535632
|
age_group |
2 | -.0642524 .4220514 -0.15 0.879 -
.8914579 .7629532
3 | .212655 .4202494 0.51 0.613 -
.6110188 1.036329
4 | .1865211 .4308455 0.43 0.665 -
.6579206 1.030963
|
race |
2 | .0563709 .3347615 0.17 0.866 -
.5997496 .7124913
3 | -.2108587 .1986695 -1.06 0.289 -
.6002438 .1785264
4 | .0311918 .2726795 0.11 0.909 -
.5032501 .5656337
5 | 1.346312 .743062 1.81 0.070 -
.1100633 2.802686
|
ed |
2 | .0507851 .2473453 0.21 0.837 -
.4340029 .5355731
3 | .0444191 .2814848 0.16 0.875 -
.507281 .5961191
4 | .2478551 .5905997 0.42 0.675 -
.9096991 1.405409
|
pov |
2 | .0399157 .2542523 0.16 0.875 -
.4584097 .538241
3 | .2269408 .2120195 1.07 0.284 -
.1886097 .6424913</td>
</tr>
</table>

© 2022 Lewis KH et al. *JAMA Network Open.*

<table>
<tr><td></td><td></td><td>4 | .5665763 .2635237 2.15 0.032
.0500794 1.083073
|
1.acgge3 | -.213463 .1852939 -1.15 0.249 -
.5766323 .1497063
1.female | -.9189754 .1699088 -5.41 0.000 -
1.251991 -.5859602
1.hypertension | -.2604579 .1694766 -1.54 0.124 -
.592626 .0717102
_cons | -1.270162 .4594238 -2.76 0.006 -
2.170616 -.3697079
-------------------------+--

/lnalpha | -.0644311 .0725506 -0.89 0.374 -
.2066277 .0777656
-------------------------+--
------
alpha | .9376007 .0680235
.8133224 1.080869

----</td></tr>
<tr><td>Abdomen/pelvic imaging</td><td>--
| Robust
total_abdomen_rad | Coef. Std. Err. z P>|z| [95% Conf. Interval]
-------------------------+---
total_abdomen_rad |
yr |
1 | -.1955557 .1482376 -1.32 0.187 -.4860961 .0949848
2 | .2791447 .1522461 1.83 0.067 -.0192522 .5775416
3 | .1033478 .1971611 0.52 0.600 -.2830808 .4897764
4 | .0938826 .2018527 0.47 0.642 -.3017414 .4895067
|
1.sg | -.0289865 .145065 -0.20 0.842 -.3133087 .2553358
|
sg#yr |
1 1 | -.1371555 .1757271 -0.78 0.435 -.4815744 .2072633
1 2 | -.0445835 .1869958 -0.24 0.812 -.4110885 .3219215
1 3 | .234846 .2407993 0.98 0.329 -.237112 .706804
1 4 | -.1935142 .2505014 -0.77 0.440 -.684488 .2974596
|
age_group |
2 | .0021282 .1160366 0.02 0.985 -.2252993 .2295557
3 | -.4763485 .1198021 -3.98 0.000 -.7111563 -.2415408
4 | -.7347591 .1263717 -5.81 0.000 -.9824432 -.487075
|
race |
2 | -.1924545 .1554725 -1.24 0.216 -.4971751 .1122661
3 | .0109841 .0672122 0.16 0.870 -.1207494 .1427177
4 | -.040195 .1035075 -0.39 0.698 -.2430661 .162676
5 | -.067088 .1703378 -0.39 0.694 -.400944 .2667681</td><td>--
-----
| Robust
total_abdomen_rad | Coef. Std. Err. z P>|z|
[95% Conf. Interval]
-------------------------+--
------
total_abdomen_rad |
yr |
1 | -.3770861 .3668985 -1.03 0.304 -
1.096194 .3420218
2 | -.2942656 .3634861 -0.81 0.418 -
1.006685 .4181541
3 | -.5569384 .3787335 -1.47 0.141 -
1.299242 .1853656
4 | -.6270322 .4036666 -1.55 0.120 -
1.418204 .1641397
|
1.sg | -.4301078 .3844958 -1.12 0.263 -
1.183706 .3234901
|
sg#yr |
1 1 | .354811 .4707111 0.75 0.451 -
.5677658 1.277388
1 2 | .3804747 .4742564 0.80 0.422 -
.5490508 1.31
1 3 | .3934004 .4714757 0.83 0.404 -
.530675 1.317476</td></tr>
</table>

© 2022 Lewis KH et al. *JAMA Network Open.*

<table>
<tr>
<td></td>
<td>|
ed |
2 | .0630809 .0796354 0.79 0.428 -.0930016 .2191633
3 | -.0376883 .1582435 -0.24 0.812 -.3478399 .2724633
4 | -.1233699 .2072678 -0.60 0.552 -.5296073 .2828674
|
pov |
2 | .0138857 .0849084 0.16 0.870 -.1525318 .1803032
3 | -.01074 .085744 -0.13 0.900 -.1787952 .1573151
4 | .0156542 .1210685 0.13 0.897 -.2216358 .2529442
|
1.acgge3 | .2297703 .0715577 3.21 0.001 .0895198 .3700209
1.female | .4384301 .0988957 4.43 0.000 .2445981 .632262
1.hypertension | -.020365 .0676494 -0.30 0.763 -.1529554 .1122254
_cons | .1002357 .2154385 0.47 0.642 -.322016 .5224874
-------------------------+--
inflate |
yr |
1 | -2.661696 .9726456 -2.74 0.006 -4.568046 -.7553455
2 | -.3019903 .2100696 -1.44 0.151 -.7137191 .1097386
3 | -.0620065 .2502977 -0.25 0.804 -.552581 .428568
4 | .0496139 .2537707 0.20 0.845 -.4477677 .5469954
|
1.sg | -.2634581 .1849179 -1.42 0.154 -.6258904 .0989743
|
sg#yr |
1 1 | -.9328259 1.941494 -0.48 0.631 -4.738084 2.872432
1 2 | .4513306 .2500427 1.81 0.071 -.0387441 .9414052
1 3 | .7435599 .2932645 2.54 0.011 .1687721 1.318348
1 4 | .0624302 .3318897 0.19 0.851 -.5880616 .712922
|
age_group |
2 | .3024211 .2025958 1.49 0.136 -.0946592 .6995015
3 | .4722787 .2061769 2.29 0.022 .0681794 .876378
4 | .7401905 .2233259 3.31 0.001 .3024798 1.177901
|
race |
2 | .1116444 .2579813 0.43 0.665 -.3939897 .6172785
3 | .1808536 .1160929 1.56 0.119 -.0466842 .4083915
4 | -.1386314 .2082358 -0.67 0.506 -.546766 .2695032
5 | .1748848 .3224224 0.54 0.588 -.4570516 .8068211
|
ed |
2 | .1842661 .1358511 1.36 0.175 -.0819972 .4505294
3 | .0114366 .2499639 0.05 0.964 -.4784836 .5013569
4 | .2939916 .3763352 0.78 0.435 -.4436119 1.031595
|
pov |
2 | -.0676816 .1494998 -0.45 0.651 -.3606958 .2253325
3 | -.1026687 .1493343 -0.69 0.492 -.3953585 .190021</td>
<td>1 4 | .2054494 .5077698 0.40 0.686 -
.7897611 1.20066
|
age_group |
2 | .557008 .3495337 1.59 0.111 -
.1280655 1.242082
3 | -.3375811 .3567197 -0.95 0.344 -
1.036739 .3615767
4 | -.3837474 .354562 -1.08 0.279 -
1.078676 .3111813
|
race |
2 | .6753875 .3454626 1.96 0.051 -
.0017067 1.352482
3 | -.0276354 .1689963 -0.16 0.870 -
.3588621 .3035912
4 | .2257692 .2283513 0.99 0.323 -
.2217911 .6733295
5 | -.2729298 .7962789 -0.34 0.732 -
1.833608 1.287748
|
ed |
2 | -.0895842 .1904083 -0.47 0.638 -
.4627776 .2836091
3 | .0442398 .3550601 0.12 0.901 -
.6516653 .7401449
4 | -.3848275 .399338 -0.96 0.335 -
1.167516 .3978606
|
pov |
2 | -.0961186 .2626646 -0.37 0.714 -
.6109317 .4186946
3 | -.0925978 .1839168 -0.50 0.615 -
.4530681 .2678726
4 | .0459773 .2559347 0.18 0.857 -
.4556455 .5476001
|
1.acgge3 | .1131923 .166953 0.68 0.498 -
.2140296 .4404142
1.female | .5478602 .2461612 2.23 0.026
.0653932 1.030327
1.hypertension | -.1355869 .1810495 -0.75 0.454 -
.4904374 .2192635
_cons | .1451835 .4478017 0.32 0.746 -
.7324917 1.022859
-------------------------+--
------
inflate |
yr |</td>
</tr>
</table>

© 2022 Lewis KH et al. *JAMA Network Open.*

<table>
<tr>
<td></td>
<td>4 | -.2108849 .2208695 -0.95 0.340 -.6437811 .2220114
|
1.acgge3 | -.4641795 .1285757 -3.61 0.000 -.7161832 -.2121758
1.female | -.8243302 .145736 -5.66 0.000 -1.109968 -.5386928
1.hypertension | -.3119441 .1177155 -2.65 0.008 -.5426622 -.081226
_cons | .7209514 .3388766 2.13 0.033 .0567654 1.385137
-------------------------+--
/lnalpha | .8754131 .09037 9.69 0.000 .6982913 1.052535
-------------------------+--
alpha | 2.399867 .2168759 2.010315 2.864904
--</td>
<td>1 | -1.460028 .7154003 -2.04 0.041 -
2.862187 -.0578694
2 | -.1933092 .4957911 -0.39 0.697 -
1.165042 .7784234
3 | -.3782036 .5882908 -0.64 0.520 -
1.531232 .7748252
4 | -.2806862 .587517 -0.48 0.633 -
1.432198 .8708259
|
1.sg | -.189997 .6041236 -0.31 0.753 -
1.374058 .9940635
|
sg#yr |
1 1 | -.9475196 2.316719 -0.41 0.683 -
5.488205 3.593166
1 2 | .1330086 .7938184 0.17 0.867 -
1.422847 1.688864
1 3 | .6734185 .8118058 0.83 0.407 -
.9176917 2.264529
1 4 | -1.216738 1.317379 -0.92 0.356 -
3.798753 1.365277
|
age_group |
2 | 1.027238 .9856948 1.04 0.297 -
.9046883 2.959164
3 | .1812561 1.098274 0.17 0.869 -
1.971322 2.333834
4 | .94006 1.031286 0.91 0.362 -
1.081224 2.961344
|
race |
2 | .472357 .7147267 0.66 0.509 -
.9284817 1.873196
3 | -.2528133 .4685397 -0.54 0.589 -
1.171134 .6655077
4 | .5083793 .3971742 1.28 0.201 -
.2700678 1.286826
5 | 1.564577 1.274361 1.23 0.220 -
.9331244 4.062278
|
ed |
2 | .1731931 .4248122 0.41 0.683 -
.6594235 1.00581
3 | 1.247486 .646371 1.93 0.054 -
.019378 2.51435
4 | -.2446681 1.014291 -0.24 0.809 -
2.232642 1.743306
|
pov |</td>
</tr>
</table>

© 2022 Lewis KH et al. *JAMA Network Open.*

		2 | -.3886233 .5700626 -0.68 0.495 -<br>1.505925 .7286788<br>3 | -1.128957 .531611 -2.12 0.034 -<br>2.170895 -.0870183<br>4 | -.5109999 .6143795 -0.83 0.406 -<br>1.715162 .6931618<br>| 1.acgge3 | -.6081891 .4441937 -1.37 0.171 -<br>1.478793 .2624144<br>1.female | -.3411348 .6038288 -0.56 0.572 -<br>1.524618 .8423479<br>1.hypertension | -.2855956 .4094547 -0.70 0.485 -<br>1.088112 .5169208<br>_cons | .125618 1.150809 0.11 0.913 -<br>2.129925 2.381161<br>-------------------------+--<br>------<br>/lnalpha | 1.029773 .1608771 6.40 0.000<br>.7144595 1.345086<br>-------------------------+--<br>------<br>alpha | 2.80043 .4505251 2.043082<br>3.838517<br>--<br>-----
All other imaging	--<br>| Robust<br>total_other_rad | Coef. Std. Err. z P>|z| [95% Conf. Interval]<br>-------------------------+--<br>total_other_rad | yr | 1 | -.0379002 .0622005 -0.61 0.542 -.1598109 .0840106<br>2 | .0398072 .0663583 0.60 0.549 -.0902527 .169867<br>3 | .1324769 .0762029 1.74 0.082 -.0168781 .2818319<br>4 | .1828783 .0767969 2.38 0.017 .032359 .3333975<br>| 1.sg | .0040713 .0532781 0.08 0.939 -.100352 .1084945<br>| sg#yr | 1 1 | .0194712 .071259 0.27 0.785 -.1201938 .1591362<br>1 2 | .1051079 .0752471 1.40 0.162 -.0423736 .2525894<br>1 3 | .11319 .0935499 1.21 0.226 -.0701644 .2965444<br>1 4 | .1096833 .0966688 1.13 0.257 -.0797841 .2991506<br>| age_group | 2 | .3760606 .0913967 4.11 0.000 .1969263 .5551949<br>3 | .5220962 .0828653 6.30 0.000 .3596831 .6845093<br>4 | .7297252 .0849387 8.59 0.000 .5632483 .8962021<br>| race |	--<br>-----<br>| Robust<br>total_other_rad | Coef. Std. Err. z P>|z| [95%<br>Conf. Interval]<br>-------------------------+--<br>------<br>total_other_rad | yr | 1 | -.0425677 .0859381 -0.50 0.620 -<br>.2110033 .1258679<br>2 | -.0413477 .0939453 -0.44 0.660 -<br>.2254771 .1427816<br>3 | .0043744 .0798881 0.05 0.956 -<br>.1522035 .1609522<br>4 | .087672 .0914481 0.96 0.338 -<br>.091563 .266907<br>| 1.sg | .0271132 .09187 0.30 0.768 -<br>.1529487 .2071751<br>| sg#yr | 1 1 | .0069314 .1235316 0.06 0.955 -<br>.2351861 .249049

© 2022 Lewis KH et al. *JAMA Network Open.*

<table>
<tr>
<td></td>
<td>2 | -.0793666 .0872523 -0.91 0.363 -.250378 .0916448
3 | -.0753587 .0393573 -1.91 0.056 -.1524976 .0017802
4 | -.1118873 .0577348 -1.94 0.053 -.2250454 .0012708
5 | -.1790106 .1093398 -1.64 0.102 -.3933126 .0352914
|
ed |
2 | .0149535 .0507005 0.29 0.768 -.0844176 .1143247
3 | -.0710783 .0608643 -1.17 0.243 -.1903701 .0482135
4 | -.030884 .1395554 -0.22 0.825 -.3044076 .2426396
|
pov |
2 | .0827759 .048277 1.71 0.086 -.0118453 .1773971
3 | .1429467 .0470154 3.04 0.002 .0507983 .2350951
4 | .1655348 .0605408 2.73 0.006 .0468769 .2841926
|
1.acgge3 | .3178294 .0337396 9.42 0.000 .251701 .3839577
1.female | .4084991 .0497948 8.20 0.000 .3109032 .5060951
1.hypertension | .1209183 .0361493 3.34 0.001 .0500671 .1917696
_cons | .2434356 .1137372 2.14 0.032 .0205147 .4663566
--------------------------+--
inflate |
yr |
1 | -.0732541 .1469905 -0.50 0.618 -.3613502 .214842
2 | .2456098 .1324116 1.85 0.064 -.013912 .5051317
3 | .5382672 .1654236 3.25 0.001 .2140428 .8624915
4 | .396837 .1839154 2.16 0.031 .0363695 .7573045
|
1.sg | .0169837 .1250122 0.14 0.892 -.2280357 .2620031
|
sg#yr |
1 1 | -.0318984 .1668513 -0.19 0.848 -.3589208 .2951241
1 2 | .1564648 .1526964 1.02 0.306 -.1428146 .4557442
1 3 | -.0649259 .1876706 -0.35 0.729 -.4327534 .3029016
1 4 | .0744849 .2122038 0.35 0.726 -.3414269 .4903968
|
age_group |
2 | .1510374 .1520753 0.99 0.321 -.1470247 .4490994
3 | -.4830356 .148784 -3.25 0.001 -.7746469 -.1914243
4 | -.6764059 .1582875 -4.27 0.000 -.9866438 -.3661681
|
race |
2 | .0750496 .2284712 0.33 0.743 -.3727458 .522845
3 | -.0420216 .0877622 -0.48 0.632 -.2140324 .1299892
4 | .0069411 .1244268 0.06 0.956 -.236931 .2508133
5 | .1996168 .2727378 0.73 0.464 -.3349394 .734173
|
ed |
2 | -.0380572 .1112456 -0.34 0.732 -.2560947 .1799802
3 | -.0173422 .1484819 -0.12 0.907 -.3083614 .2736769
4 | .0435651 .2265942 0.19 0.848 -.4005514 .4876815</td>
<td>1 2 | .0118572 .1257575 0.09 0.925 -
.2346229 .2583373
1 3 | .0378931 .119397 0.32 0.751 -
.1961207 .2719069
1 4 | .0247047 .1266488 0.20 0.845 -
.2235223 .2729317
|
age_group |
2 | .6737685 .237 2.84 0.004 .209257
1.13828
3 | .9024551 .239862 3.76 0.000
.4323341 1.372576
4 | 1.100449 .2385826 4.61 0.000
.6328353 1.568062
|
race |
2 | .2827378 .2569843 1.10 0.271 -
.2209421 .7864178
3 | -.0234579 .0687502 -0.34 0.733 -
.1582059 .1112902
4 | -.023985 .1032952 -0.23 0.816 -
.2264398 .1784698
5 | -.2219829 .3027519 -0.73 0.463 -
.8153657 .3713999
|
ed |
2 | .0257687 .0873655 0.29 0.768 -
.1454645 .1970018
3 | -.0821178 .0991588 -0.83 0.408 -
.2764656 .11223
4 | -.384681 .1771849 -2.17 0.030 -
.7319569 -.037405
|
pov |
2 | .0004689 .0896229 0.01 0.996 -
.1751888 .1761266
3 | .167358 .0897601 1.86 0.062 -
.0085686 .3432847
4 | .2772439 .1097998 2.52 0.012
.0620402 .4924476
|
1.acgge3 | .4105169 .0650012 6.32 0.000
.283117 .5379168
1.female | .5195116 .0788069 6.59 0.000
.365053 .6739702
1.hypertension | .0825699 .0654193 1.26 0.207 -
.0456495 .2107893
_cons | -.2616713 .2670422 -0.98 0.327 -
.7850643 .2617218</td>
</tr>
</table>

© 2022 Lewis KH et al. *JAMA Network Open.*

	| pov | 2 | .0240829 .1023607 0.24 0.814 -.1765404 .2247063<br>3 | .0004172 .1031103 0.00 0.997 -.2016753 .2025097<br>4 | -.0144732 .1356775 -0.11 0.915 -.2803963 .2514499<br>| 1.acgge3 | -.5744171 .0787924 -7.29 0.000 -.7288472 -.4199869<br>1.female | -1.010101 .0879681 -11.48 0.000 -1.182515 -.8376864<br>1.hypertension | -.2450277 .0786258 -3.12 0.002 -.3991315 -.0909239<br>_cons | .4584943 .1965485 2.33 0.020 .0732664 .8437223<br>-------------------------+--<br>/lnalpha | -.2616188 .0513702 -5.09 0.000 -.3623026 -.160935<br>-------------------------+--<br>alpha | .7698044 .039545 .6960717 .8513474<br>--	-------------------------+--<br>------<br>inflate | yr | 1 | .0495726 .2355439 0.21 0.833 -<br>.412085 .5112301<br>2 | .109554 .2362517 0.46 0.643 -<br>.3534908 .5725989<br>3 | .252766 .235063 1.08 0.282 -<br>.207949 .713481<br>4 | .0635375 .2574814 0.25 0.805 -<br>.4411167 .5681917<br>| 1.sg | .1004787 .2579158 0.39 0.697 -<br>.4050269 .6059843<br>| sg#yr | 1 1 | -.3976554 .3361245 -1.18 0.237 -<br>1.056447 .2611365<br>1 2 | -.0863065 .3217016 -0.27 0.788 -<br>.7168301 .544217<br>1 3 | -.2370975 .3278396 -0.72 0.470 -<br>.8796513 .4054563<br>1 4 | -.2523936 .3415005 -0.74 0.460 -<br>.9217222 .416935<br>| age_group | 2 | .8721561 .6170966 1.41 0.158 -<br>.3373311 2.081643<br>3 | -.158068 .6323591 -0.25 0.803 -<br>1.397469 1.081333<br>4 | -.3161012 .6342747 -0.50 0.618 -<br>1.559257 .9270543<br>| race | 2 | .1306915 .3634339 0.36 0.719 -<br>.5816258 .8430088<br>3 | .1176487 .1831661 0.64 0.521 -<br>.2413502 .4766475<br>4 | -.0550087 .2556491 -0.22 0.830 -<br>.5560718 .4460544<br>5 | .9091766 .4211653 2.16 0.031<br>.0837077 1.734645<br>| ed | 2 | .0346757 .2029396 0.17 0.864 -<br>.3630786 .43243<br>3 | .1384436 .323627 0.43 0.669 -<br>.4958536 .7727408

© 2022 Lewis KH et al. *JAMA Network Open.*

		4 | -.3354283 .6525974 -0.51 0.607 -1.614496 .9436391<br>| pov | 2 | -.2746414 .2329163 -1.18 0.238 -.731149 .1818662<br>3 | -.4142622 .2385675 -1.74 0.082 -.8818459 .0533214<br>4 | -.1818734 .3037588 -0.60 0.549 -.7772296 .4134828<br>| 1.acgge3 | -.3597876 .1653958 -2.18 0.030 -.6839574 -.0356178<br>1.female | -1.22646 .1526042 -8.04 0.000 -1.525559 -.9273612<br>1.hypertension | -.1687529 .1666045 -1.01 0.311 -.4952918 .1577859<br>_cons | .1450575 .6495473 0.22 0.823 -1.128032 1.418147<br>-------------------------+--<br>/lnalpha | -.4301555 .0808482 -5.32 0.000 -.5886151 -.271696<br>-------------------------+--<br>alpha | .6504079 .0525843 .5550955 .7620859<br>--

eTable 9: STATA outputs for all models
a. The zero-inflated negative binomial model regression outputs were extracted directly from the STATA 17 log files. For each outcome, the coefficients for both the inflated part and main part of the model were provided. Negative binomial models were used for PCP and specialist visits due to convergence issues

© 2022 Lewis KH et al. *JAMA Network Open.*

**eTable 10.** Final Time Series Model After Backward Variable Selection for the Differenced Outcomes Between SG and RYBG[a]					
**Outcome Measure**	**Variable**	**Estimate**	**P-value**	**Lower limit of 95% CI**	**Upper limit of 95% CI**
Total Ambulatory Costs	Intercept	-1.186254284	0.959460108	-69.59506368	67.22255511
	trend	*Eliminated*			
Laboratory Costs	Intercept	8.53756081	0.005261583	4.826914892	12.24820673
	trend	*Eliminated*			
Office Visit Costs	Intercept	1.484189963	0.728178844	-10.89341035	13.86179028
	trend	*Eliminated*			
Radiology Costs	Intercept	7.577394754	0.514911946	-25.17494862	40.32973813
	trend	*Eliminated*			
Prescription Costs	Intercept	-36.49429201	0.058274095	-75.37761809	2.389034073
	trend	*Eliminated*			
All Other outpatient Costs	Intercept	17.7088922	0.719825331	-125.2809275	160.6987119
	trend	*Eliminated*			

eTable 10: Final Time Series Model After Backward Variable Selection for the Differenced Outcomes between SG and RYGB – Checking for Parallel Trends
a. We used backward variable selection with a cutoff of p-value at 0.05 for the time series models. All models started with both intercept and trend(i.e. quarter). Intercept was kept in the final model regardless of the p-value. As shown in the output, the trend term was eliminated from all models, providing evidence of parallel trend for all outcomes.

© 2022 Lewis KH et al. *JAMA Network Open.*

eReferences.

1. Smith LH, VanderWeele TJ. Bounding Bias Due to Selection. *Epidemiology*. 2019;30(4):509-516. doi:10.1097/EDE.0000000000001032

2. Mathur MB, VanderWeele TJ. Sensitivity Analysis for Unmeasured Confounding in Meta-Analyses. *J Am Stat Assoc*. 2020;115(529):163-172. doi:10.1080/01621459.2018.1529598

3. VanderWeele TJ, Ding P. Sensitivity Analysis in Observational Research: Introducing the E-Value. *Ann Intern Med*. 2017;167(4):268-274. doi:10.7326/M16-2607

© 2022 Lewis KH et al. *JAMA Network Open.*
